# Comparative Analysis of DNA Word Abundances in Four Yeast Genomes Using a Novel Statistical Background Model

**DOI:** 10.1371/journal.pone.0058038

**Published:** 2013-03-05

**Authors:** Ramkumar Hariharan, Reji Simon, M. Radhakrishna Pillai, Todd D. Taylor

**Affiliations:** 1 Cancer Research Program, Rajiv Gandhi Center for Biotechnology, Thiruvananthapuram, Kerala, India; 2 Laboratory for MetaSystems Research, RIKEN Quantitative Biology Center, Tsurumi-ku, Yokohama, Kanagawa, Japan; Institut Jacques Monod, France

## Abstract

Previous studies have shown that the identification and analysis of both abundant and rare k-mers or “DNA words of length k” in genomic sequences using suitable statistical background models can reveal biologically significant sequence elements. Other studies have investigated the uni/multimodal distribution of k-mer abundances or “k-mer spectra” in different DNA sequences. However, the existing background models are affected to varying extents by compositional bias. Moreover, the distribution of k-mer abundances in the context of related genomes has not been studied previously. Here, we present a novel statistical background model for calculating k-mer enrichment in DNA sequences based on the average of the frequencies of the two (k-1) mers for each k-mer. Comparison of our null model with the commonly used ones, including Markov models of different orders and the single mismatch model, shows that our method is more robust to compositional AT-rich bias and detects many additional, repeat-poor over-abundant k-mers that are biologically meaningful. Analysis of overrepresented genomic k-mers (4≤k≤16) from four yeast species using this model showed that the fraction of overrepresented DNA words falls linearly as k increases; however, a significant number of overabundant k-mers exists at higher values of k. Finally, comparative analysis of k-mer abundance scores across four yeast species revealed a mixture of unimodal and multimodal spectra for the various genomic sub-regions analyzed.

## Introduction

The availability of completely sequenced genomes has made possible empirical, as opposed to the earlier theoretical, studies of the distributions of “DNA words” or “k-mers of length k” in genomic DNA sequences [1]–[5]. Apart from a few recent studies [4], [5], the vast majority of investigations in this area have attempted to analyze over- or underrepresented k-mers in different genomic regions. While a few of these studies have attempted to identify and catalog the set of missing elements (dubbed “nullomers”) in genomes [6]–[8] others have focused on detecting over-represented k-mers in select genomic regions for the identification of functional elements [9]–[15].

The identification of over- and underrepresented k-mers in a DNA sequence typically involves the following steps [16]: (a) choosing the genomic region (e.g., gene upstream regions) to be analyzed, (b) using a suitable counting method (e.g., overlapping k-mers may or may not be counted), (c) selecting an appropriate statistical background or null model for predicting expected k-mer frequencies, (d) using appropriate statistics to score the observed k-mer frequency against the expected background frequency (e.g. binomial probabilities, fold enrichment scores and Z-scores). Different background models have been proposed for calculating k-mer distributions in random sequences. While initial, theoretical studies supported the use of a Markov model of order zero (Bernoulli model) or one [1], [2], subsequent probabilistic models, which test empirical word counts in different whole genomes, recommend the use of Markov models of orders close to k/2 as optimal null models [16]. Additionally, Hampson et al. reported a novel and efficient statistical background model based on single mismatches. However, it has been noted that the existing background models have varying degrees of AT-rich compositional bias, i. e., the list of over-represented k-mers identified by each model is likely to contain significantly more AT-rich elements if the input genomic sequences are AT-rich, and vice versa.

Explorations of k-mer frequency distributions (or “k-mer spectra”) for genomic regions in different species have allowed us to take new perspectives on the complexity of genomes and to find associations between k-mer spectral modality and GC content, as well as those between CpG suppression and modality [3], [4]. These studies have reported unimodal genomic k-mer spectra for the vast majority of analyzed species, with the striking exception of tetrapod animal genomes where the k-mer distributions are typically multimodal [3].

It is noteworthy that comparative analysis of k-mer enrichment for a set of related species, which is likely to yield more insights into the nature of these distributions, has not been reported to date.

Here, we present a new statistical background model based on the average frequencies of the corresponding two (k-1) mers for each k-mer (e.g., the two corresponding 6-mers of the 7-mer ‘TAGTGTA’ are ‘TAGTGT’ and ‘AGTGTA’). We show that calculating over-representation using this model identifies many additional over-abundant k-mers not detected by other existing models. Moreover, our method is less prone to AT-rich compositional bias. Since the list of top over-represented k-mers predicted by our model substantially overlaps with that of other optimal models, we also suggest that our new background model, tested here on the yeast genome, can be used to detect meaningful, over-represented k-mers in any genome.

The idea of extending or removing nucleotides from either end of a nucleotide sequence has been used before to identify “minimal absent words” in a sequence [17]. Minimal absent words are defined as absent words with the following property: words that are found in then sequence by removing the left- or right-most characters. However, in this manuscript we are not exploring minimal absent words or absent words.

In addition, we explore the landscape of overrepresented genomic k-mers in the budding yeast *Saccharomyces cerevisiae* (*S.cerevisiae*) for 4≤k≤16 and find that the fraction of overrepresented genomic k-mers decreases rapidly as the value of k increases beyond nine. However, even at higher values of k, a small but significant number of overrepresented k-mers exists, hinting at the existence of even longer, perfectly conserved (intra-species) elements relevant to regulatory function or structure.

More importantly, we use our novel background model to carry out a comparative study of k-mer enrichment in four related yeast species. We show that the distribution of k-mer fold enrichment scores derived from almost all genomic regions analyzed (e.g., whole genome, regions 1 kb up- and downstream of open reading frames (ORFs), compared across different combinations of species show unimodal or multimodal distributions. This is in sharp contrast to the unimodal genomic k-mer spectra for the *S. cerevisiae* genome reported earlier [4]. We suggest that the multimodal distributions in such comparisons may result from the existence of different classes of functional or structural elements that are not only conserved across species but also show similar ranges of fold enrichment scores.

## Materials and Methods

The complete genomes of *S.cerevisiae*, *S.bayanus*, *S.paradoxus* and *S.mikatae*, along with the feature tables for *S.cerevisiae* were downloaded from the Saccharomyces Genome Database (SGD) (http://www.yeastgenome.org/ March 2009). For *S.bayanus*, *S.paradoxus* and *S.mikatae*, the contig files from the Washington University assembly which also incorporates the MIT assemblies were chosen. We also downloaded the sequence files containing the regions 1 kb up- and downstream of all ORFs, and the ‘not-annotated’ intergenic sequences for all four yeast species.

### Counting identical k-mer occurrences

We wrote a Perl script to count the number of non-overlapping occurrences of each observed k-mer in each of the genomes. Counts for each k-mer from the forward, as well as the reverse, strand were aggregated and considered for all calculations.

### Calculating the enrichment score and Z-score using a novel statistical background model

To determine the fold enrichment score of a k-mer, we first calculated the expected number of occurrences of the k-mer using a novel null model.

Let ‘
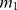
’ and ‘
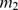
’ represent the counts of the two different (k-1) mers (or subwords of length k-1) corresponding to a given k-mer. Let the total number of (k-1) mers (from a genomic region of length 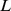) be represented by ‘
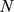
’.

The frequency of each (k-1) mer, ‘
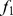
’ and ‘
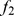
’ are given by:
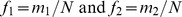
Expected number of occurrences of the k-mer (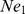), based on 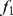:
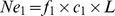
Expected number of occurrences of the k-mer (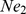), based on 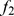:
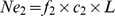
where 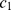 and 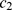 are the frequencies of the mononucleotides that, along with the (k-1) mer, constitute the full k-mer.

Fold enrichment scores 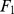 and 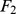 are given by:
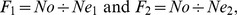
where ‘
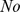
’ is the observed number of occurrences of a k-mer in the genome.

Finally, the fold enrichment score for the k-mer is calculated as the average of the two fold enrichment scores, based on its two corresponding (k-1) mers.

Fold enrichment score = 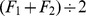


And the Z-score is given by:
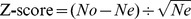
where ‘
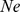
’ is the average expected count for the k-mer.

### Comparing our statistical background model against other existing null models

We compared lists of top 20 k-mers, ranked by fold enrichment and by Z-score using our novel background model with those obtained by applying the existing background models. We first compiled the regions 500 bp upstream of all *S.cerevisiae* ORFs and then applied three different methods to calculate over-representation of 8-mers: our “average (k-1) mer” or the Ak-1method, the C0/C1 method described previously by Hampson et al., and Markov models based-relative abundance scores (both Z-score and binomial probabilities) for orders 3 to 6. (In this manuscript, Markov models of varying orders are referred to by the following notation: MM followed by a number corresponding to their order and ending with either ‘Z’ or ‘B’ based on whether Z-score or binomial statistics was used for ranking the k-mers. Thus, MM3B refers to a Markov model of order 3 and a ranking based on binomial probabilities). The C0/C1 method of calculating over-representation was implemented as described earlier [16] based on the single mismatch model. To get Z-scores and binomial probabilities for 8-mer relative abundances in the input dataset, based on Markov models, we used the ‘oligo-analysis’ feature of the online RSAT tool (http://rsat.ulb.ac.be/, August 2011) with default settings except for the following parameters: oligomer length was set to 8, and the background model was estimated from the input sequence and was specified for each run (varied from 3 to 6). For each order, 8-mer rankings were then ordered based on Z-scores or on binomial probabilities [13]. The list of top 20 8-mer sequences from our average (k-1) mer method was then compared with that from both the C0/C1 method and the Markov model methods.

To test whether our method can identify k-mers artificially over-represented in a large number of sequences, we used a protocol that was previously described [16]. Briefly, we generated 6,000 random sequences, each 500 bases long, and introduced the 8-mer ‘ATGCCGTA’, making sure that the random strings had the same GC content as that of *S.cerevisiae*. We then applied our method to calculate and rank 8-mers in this dataset by their fold enrichment and Z-score values.

### Identifying transcription factor binding motifs in lists of k-mers

We investigated what fraction of each list of top twenty k-mers (7<k<11) represent matches to known transcription factor binding motifs by searching for each 8-mer in the list of known TF binding sites contained in Yeastract. Both ‘Yeastract binding site inside of the inserted motif’ as well as ‘Inserted motif inside Yeastract binding site’ hits were considered [18].

### Comparing lists of ranked k-mers across different yeast species

We generated lists of k-mers for each of the above-mentioned sequence datasets and ranked them by decreasing fold enrichment score by applying our average (k-1) method. For each yeast species, the rank lists of k-mers were further divided into bins of k-mers using the following protocol: we first calculated the statistical range of fold enrichment scores for each list. Next, we divided the k-mer list into 8 bins of k-mers, all defined by equal intervals of the fold enrichment score. Independently, we also applied the C0/C1 method to obtain a similar number of bins for the same datasets (for comparison and to test whether the results are robust to methodology differences). Comparisons were then carried out by checking how many k-mers were identical between each corresponding bin of k-mers across the different yeast species in all possible combinations that included *S.cerevisiae*, which was used as the reference in each such comparison. Since corresponding bins from two or more species can contain a different number of k-mers, we first determined the number of k-mers in the *S.cerevisiae* bin. Then, we enumerated *S.cerevisiae* k-mers that were also found in the corresponding bins from other species. The number of such identical k-mers was then divided by the total number of k-mers in the *S.cerevisiae* bin, and the result was expressed as a percentage.

## Results

### A new statistical background model for calculating k-mer enrichment and its comparison with existing models

Because the detection of overrepresented k-mers with commonly used background models are biased toward AT-richness and repeat-rich motifs, we developed a novel statistical background model to calculate k-mer fold enrichment scores. To accomplish this, we carried out the following steps: (a) we first determined the number of occurrences of the two (k-1) mers corresponding to each k-mer, (b) we computed the expected frequencies of each k-mer by multiplying the (k-1) mer frequencies with that of the remaining nucleotide that makes up the k-mer, (c) the fold enrichment scores, F_1_ and F_2_, (based on each (k-1) mer), were then calculated as the ratio of the observed number of occurrences of a k-mer to its expected number of occurrences, (d) the average of the two fold enrichment scores was taken to obtain the fold enrichment score for each k-mer and, (e) finally, the Z-score was calculated on the fold enrichment score (see methods).

We then compared our background model with some of the existing, commonly used ones. For comparison purposes, we selected the two most optimal models: the C0/C1 method [16] and Markov models of different orders [13]. We used a dataset comprised of the regions 500 bp upstream of all *S.cerevisiae* ORFs. Fold enrichment scores for 8-mers in this dataset were calculated using (a) our method (the Ak-1 method), (b) the C0/C1 method and, (c) Markov models of orders 3, 4, 5 and 6 (see methods). The scored k-mers were then ranked by decreasing fold enrichment score (for the average (k-1) mer method and the C0/C1 method) and by decreasing Z-scores or binomial probabilities (for the Markov model based results).

The list of top 20 8-mers identified by the Ak-1 method was then compared against that from each of the different models (Table 1). A more extensive comparison involving the top 500 8-mers obtained from the various methods is also presented (Table S1). Interestingly, the top two 8-mers identified by our method and the C0/C1 protocol (column b) were identical and, in addition, there were five 8-mers common to both lists. The top 20 8-mers obtained by applying a statistical background model based on the MM4Z method (column c) or the MM4B method (column d), shared six 8-mers with the top 20 calculated by our method. While we found six 8-mers common to the top 20 list from our method and the MM3Z method (column e), only four 8-mers were shared between our method and the MM3B method (column f).

**Table 1:** Comparison of the top 20 8-mers calculated using six different methods.

No	Ak-1 method (a)	C0/C1 (b)	MM4 Z-score (c)	MM4 Binomial (d)	MM3 Z-score (e)	MM3 Binomial (f)
1	CCTCGAGG	CCTCGAGG ^1^	GCGATGAG ^2^	TAATATTA	*TATATATA*	*TATATATA*
2	GCGATGAG	GCGATGAG ^2^	TAATATTA	GCGATGAG ^2^	GCGATGAG ^2^	*ATATATAT*
3	**CCCAGCGC**	TAGCCGCC ^14^	CCTCGAGG ^1^	*AAAAGAAA*	*ATATATAT*	GCGATGAG ^2^
4	**CCGAGTGG**	CTCGAGGA^8^	CGGTGTTA	CGGTGTTA	CCTCGAGG ^1^	*TTTTTTTC*
5	GGAAGCTG	CGGTGTTA	GTTACCCG	CCTCGAGG ^1^	GTTACCCG	*GAAAAAAA*
6	TCCTCGAG	TCCTCGAG ^6^	GGAAGCTG ^5^	GGAAGCTG ^5^	CTCGAGGA ^8^	*AAAAGAAA*
7	*CGCGTCGC*	TACGGTGT	CTCGAGGA ^8^	GTTACCCG	CCGGGTAA	CCTCGAGG ^1^
8	CTCGAGGA	GGCGGCTA	CGGGTAAC	CTCGAGGA ^8^	CTCATCGC	CTCGAGGA ^8^
9	GATGAGCT	CCGGGTAA	CCGGGTAA	CTAGTATA	CGGGTAAC	GTTACCCG
10	**GATGACGC**	GGAAGCTG ^5^	CTAGTATA	*TTTCTTTT*	TCCTCGAG ^6^	CTCATCGC
11	ATACGGTG	GTTACCCG	ACTTCTAG	ACTTCTAG	TTACCCGG	*TTTCTTTT*
12	***GCGCGCGC***	ATACGGTG ^11^	CTCATCGC	CTCATCGC	CGATGAGC	*CATATATA*
13	**GCGCCCGC**	TTACCCGG	TCCTCGAG ^6^	CCGGGTAA	*TTTTTTTC*	CCGGGTAA
14	TAGCCGCC	*TTTTTTTC*	GATTCCTA	CGGGTAAC	*GAAAAAAA*	ATATGTAT
15	GCGACGCG	TCCGGGTA	*AAAAGAAA*	TGATAATG	GATGAGCT ^9^	ATACATAT
16	**CACGTGAC**	CGGGTAAC	GAAGCTGA	GAAGCTGA	GGAAGCTG ^5^	TCCTCGAG ^6^
17	GGATTCCT	CGATGAGC	GGATTCCT ^17^	TCCTCGAG ^6^	ATATGTAT	TATACATA
18	**GCCCCCGG**	*GAAAAAAA*	TACGGTGT	AGGAGAAC	ATTACCCG	CGGGTAAC
19	**GGCGCGTC**	CTCATCGC	TTACCCGG	GATTCCTA	CGGTGTTA	TTACCCGG
20	**ACGCAAGG**	ATTACCCG	AGGAGAAC	GGATTCCT ^17^	*AAAAGAAA*	TATGTATA
Avg GC%	73.1	56.8	50.0	46.2	45.0	34.3

8-mers derived from the region 500 bases upstream of all yeast ORFs were ranked by their relative abundance as calculated by several background models. The top 20 8-mers ranked by decreasing fold enrichment scores from (a) our novel background model, the “Ak-1 method” were compared to those from the previously described C0/C1 method (b), from Markov models of order 4 sorted by Z-scores (c), from Markov models of order 4 sorted by Binomial probabilities (d), from Markov models of order 3 ranked by Z-scores (e) and, Markov models of order 3 ranked by binomial probabilities (f). The list of 20 8-mers derived by applying our method was chosen as the reference against which the other lists were compared. 8-mers in each list that are identical to the ones in the reference list are marked with a superscript number which shows its rank in the reference column (a), while repeat-rich elements are shown italicized. The nine 8-mers identified uniquely by our method are highlighted in bold (a). The average GC content of each list is shown in the bottom row of the table.

We also noted the number of 8-mers comprised of low complexity DNA and found that our method and the MM4 method identified the least number (one each) of such k-mers in the top 20 lists. In order to quantitatively assess compositional bias of the various methods, we calculated the average GC content of the top 20 sets. Given that the input sequence dataset is AT-rich, with an average GC content of 38.2%, our method identified the most GC-rich strings with an average GC content of 73.1% for the top 20 8-mers. The MM3 method identified the fewest GC-rich sequences with an average GC content of 34.3%.

Comparative analysis of the top 20 lists for their biological relevance was carried out. We tested to see how many of the 8-mers in each list match known transcription factor recognition motifs, a comprehensive collection of which can be found in Yeastract [18] (Table S2). The list of twenty 8-mers from the Ak-1 method represents 17 TF binding motifs, whereas that from the C0/C1 approach contains only 13 TF binding sites. Yeastract motif matches for MM4Z, MM4B, MM3Z and MM3B were progressively lower, i.e., 12, 10, 9 and 6, respectively. Similar comparative analyses between the Ak-1 method and the C0/C1 approach was carried out for k = 9 and k = 10. In both cases, our method detected more TF binding sites (13 and 10, respectively) than the C0/C1 method (9 and 5, respectively) (Tables S3, S4).

To test whether our method can detect artificially enriched k-mers in a set of random sequences, we carried out a simulation previously described by Hampson et al. (see methods). Indeed, our method identified the ‘ATGCCGTA’ 8-mer (which was inserted into 6,000 500 bp long randomly generated sequences) as the top k-mer when the top 10 list was built based on decreasing Z-scores. Additionally, when we made a similar top 10 list based on decreasing fold enrichment scores, ‘ATGCCGTA’ was ranked ninth (Table 2).

**Table 2:** Top ten 8-mers derived from the set of 6000 randomly generated 500 bp sequences, each having the 8-mer insert ‘ATGCCGTA’.

Sl. No	8-mers C0/C1 (a)	8-mers average (k-1) mer method ranked by fold enrichment (b)	8-mers average (k-1) mer method ranked by Z-score (c)
1	ATGCCGTA*	CGGGGGGC	ATGCCGTA*
2	TGCCGTAA	CGGGAGAC	TATATAAT
3	AATGCCGT	CGATGCCG	TAATATAT
4	TATGCCGT	ACCCCCCC	ATATATAA
5	TGCCGTAT	CCCCCCCT	ATTATATA
6	GCCGTATA	GCCGTAGC	AATATATA
7	TAATGCCG	GCACCCCC	TTATATAT
8	ATATGCCG	CGGGTGGC	ATATATTA
9	GCCGTAAT	ATGCCGTA*	TATATATT
10	TGCCGTAG	GCACCCTC	ATATTATA

Three different sets of top ten 8-mers are shown, with the artificially inserted 8-mer marked with an asterisk (*). The first set of top ten 8-mers was obtained after applying the previously published C0/C1 method (a), whereas the other two sets were calculated using our avg (k-1) mer method and ranking the resulting 8-mers based on either their fold enrichment scores (b) or, their Z-scores (c).

### Overrepresented sequence elements in the yeast genome

We first sought to create a list of k-mers that are statistically overrepresented in the *S.cerevisiae* genome. To do this, we applied both our Ak-1 method and the C0/C1 single mismatch model to evaluate over-representation of k-mers with 4≤k≤16 (see Methods). We also identified k-mers that occurred two or more times in the entire genome.

**Figure 1:**
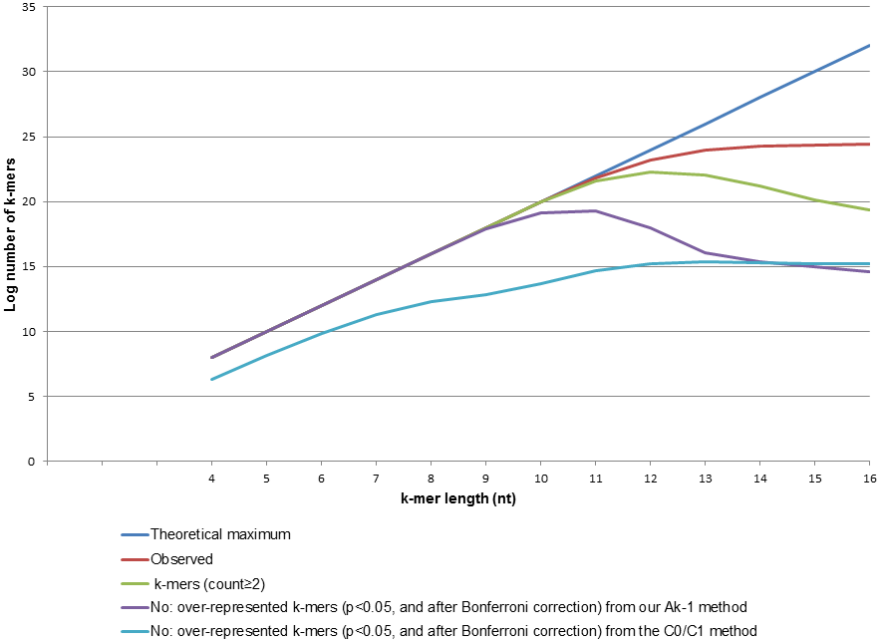
Plot of log_2_ transformed number of different k-mers found in *S. cerevisiae* genome versus k value. The red line shows the numbers of different k-mers found in the yeast genome, the green line that of k-mers found two or more times. The purple line depicts the numbers of k-mers found overrepresented in the genome by applying our Ak-1method, while the pale blue line shows a similar count arrived at by applying the C0/C1 method. All values have been plotted alongside the theoretical maximum possible number of k-mer permutations (dark blue line) for each k- value.

All theoretically possible sequence permutations were found in the ∼12 Mb yeast genome up to and including k = 8 and the numbers for ‘theoretical maximum’ and ‘observed’ deviate markedly thereafter (Figure 1). The number of over-represented k-mers for each value of k was calculated by both methods using a p-value cut-off of 0.05 after applying multiple testing correction (Bonferroni correction). Interestingly, the number of overrepresented genomic k-mers calculated by our average (k-1) method differs markedly from that of the C0/C1 method, up to k = 14, when an approximate convergence is reached (purple and pale blue lines in Figure 1). This could represent a coincident crossover point between the two lines beyond which the lines subsequently diverge again. There is some evidence for this divergence at k = 15 and k = 16, where the lines can be seen as disparate ones. Because it was not a primary goal of this study, we did not seek to identify overrepresented k- mers beyond k = 16. Up to k = 16, our Ak-1 method seems to be more sensitive in that it detects more overrepresented k-mers than the C0/C1 method.

We also determined the percentage of the number of observed k-mers (separately for each value of k where 4≤k≤16) in the whole genomes of four related yeast species (Figure S1) that can be marked as “overrepresented” by our Ak-1 method. As the value of k increases from 9 to 12, the fraction of overrepresented k-mers in the genome falls linearly.

### Comparative analysis of the distribution of fold enrichment scores of genomic k-mers across four related yeast species

How many k-mers in the genomic regions of *S.cerevisiae* have similar abundance scores across related species and what is the distribution of such k-mers? To address this question, we carried out comparative analysis between four closely related yeast species: *Saccharomyces cerevisiae (S.cerevisiae), Saccharomyces paradoxus (S.paradoxus), Saccharomyces bayanus (S.bayanus) and Saccharomyces mikatae (S.mikatae)*, all of which are members of the Saccharomyces *sensu stricto* group. We first calculated fold enrichment scores (for 7≤k≤9) using both our Ak-1 method and the C0/C1 method for k-mers from four genomic regions (i.e., whole genome, regions 1 kb upstream of ORFs, 1 kb downstream of ORFs and unannotated intergenic regions) from all four yeast species. It has been shown before that analyzing k-mers within this range of k values gives meaningful results that can be extrapolated for other values of k [16]. However, since the overrepresentation values are sensitive to the methodology used to derive the fold enrichment scores, comparative analysis across different values of k becomes meaningful only in the context of the same method. Next, for each value of k, the k-mers were ranked by decreasing fold enrichment scores. We calculated the statistical range of k-mer fold enrichment scores for each region from each species. We then divided this range into eight bins, each one defined by an equal interval of the fold enrichment score. Each bin was then populated by k-mers whose fold enrichment scores fell within the interval of fold enrichment scores flanking the bin (see methods). *S.cerevisiae* was used as the reference against which the comparisons were carried out. For each bin, the percentage of *S.cerevisiae* k-mers that have identical sequence partners in the corresponding bins from the other species being compared was calculated and plotted.

Plots of the distribution of k-mer fold enrichment scores from the comparative analyses revealed several interesting trends:

#### Comparative analysis of k-mers derived from the whole genome

**Figure 2:**
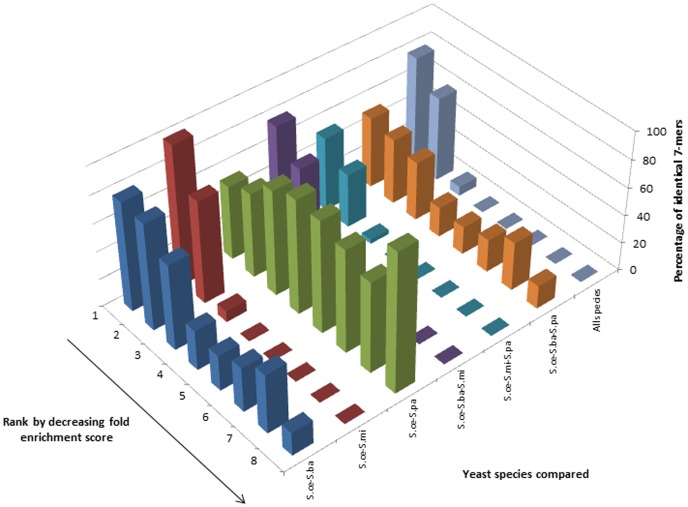
Comparative analysis of genomic 7-mers across four related yeast species. Distributions of the percentage of 7-mers in each bin from *S.cerevisiae* which have identical k-mers in the corresponding bins from other, related yeast species. The 7-mers for each species were derived from the complete genome and the fold enrichment scores were calculated based on the Ak-1 method.

Especially for 7-mers, the distributions of the comparative analysis across species are best described by a positively skewed distribution except for comparisons involving *S.paradoxus* and *S.bayanus* (Figure 2 and additional Figures S2, S6, S10, S14 and S18). The distributions of the percentage of *S.cerevisiae* k-mers with sequence-identical counterparts in *S.bayanus* and *S.paradoxus* are strikingly multimodal. The nature of the distributions are largely unaffected by the type of method used to calculate fold enrichment scores.

Multimodal k-mer distributions hint at the existence of distinct classes of k-mers in the genome whose fold enrichment scores are confined to certain score intervals. They may be associated with specific functional elements wherein the functionality affects or is affected by the relative abundance of such “instances” in the genome. Positively skewed distributions reveal that many more over-represented k-mers share the same range of fold enrichment values across species compared with underrepresented k-mers.

#### Comparative analysis of k-mers derived from regions 1 kb up- and downstream of ORFs

**Figure 3:**
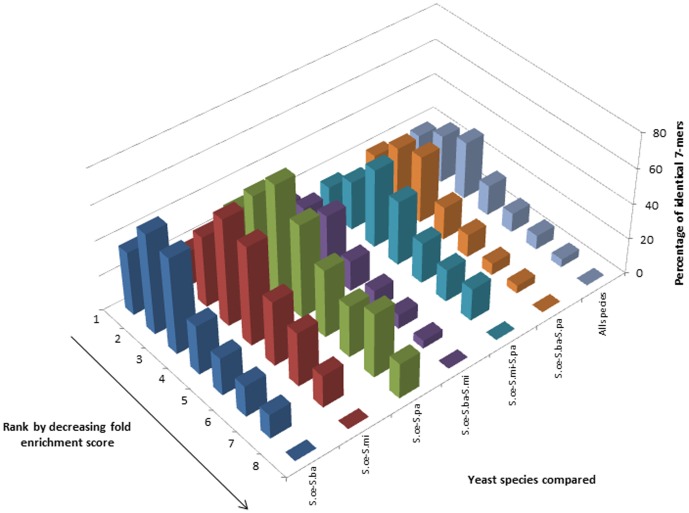
Comparative analysis of 7-mers across four related yeast species. Distributions of the percentage of 7-mers in each bin from *S.cerevisiae* which have identical k-mers in the corresponding bins from other, related yeast species. The 7-mers for each species were derived from the regions 1 kb upstream of annotated ORFs and the fold enrichment scores were calculated based on the Ak-1 method.

Comparative analysis of 7-mer fold enrichment scores for the region 1 kb upstream of all ORFs using our Ak-1 method yielded right skewed distributions for all comparisons except for the one involving *S.paradoxus*, which was bimodal. However, the modality of these distributions was not in concordance with the same analysis carried out using the C0/C1 method (Figure 3 and additional Figure S3). All comparisons for the 8-mers exhibited multimodality with both methods (Figures S7 and S11). Inconsistent results were obtained for similar analyses of 9-mers (Figures S15 and S19).

**Figure 4:**
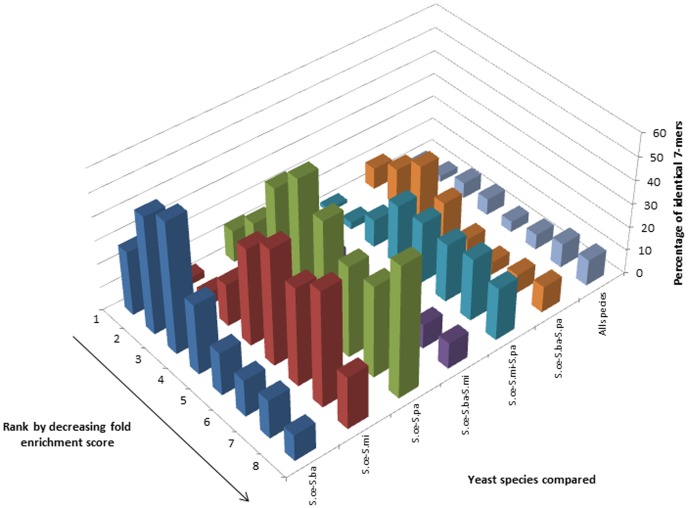
Comparative analysis of genomic 7-mers across four related yeast species. Distributions of the percentage of 7-mers in each bin from *S.cerevisiae* which have identical k-mers in the corresponding bins from other, related yeast species. The 7-mers for each species were derived from the regions 1 kb downstream of annotated ORFs and the fold enrichment scores were calculated based on the Ak-1 method.

Distributions for 7-mers derived from the region 1 kb downstream of all ORFs were typically multimodal, were robust to the fold enrichment calculation method used and similar modalities were observed for the distributions built based on 8-mers (Figure 4 and additional Figures S4, S8 and S12). No similar trends for the distributions of the 9-mers were observed (Figures S16 and S20).

Multimodal distributions may imply that there exist distinct classes of regulatory element-associated k-mers whose relative abundances in the ORF up- and downstream regions are under relatively high constraint. The list of over-represented k-mers calculated by the C0/C1 method contains many more k-mers with repeat-rich sequences than our method. This may be the reason why some calculations give different results when analyzed using the two different methods.

#### Comparative analysis of k-mers derived from unannotated intergenic regions

**Figure 5:**
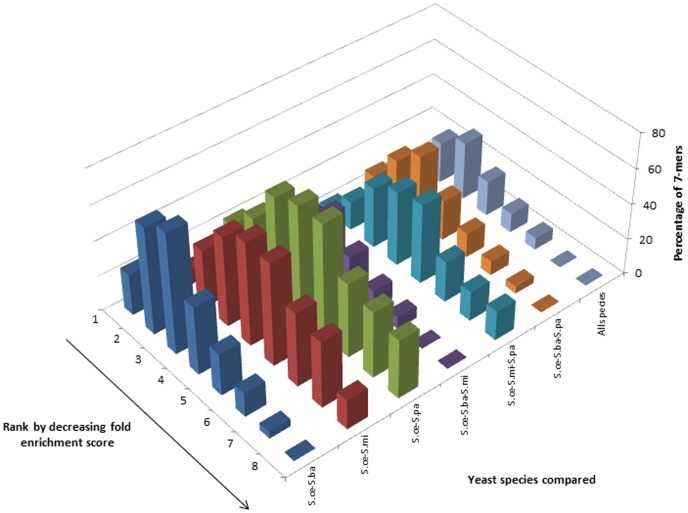
Comparative analysis of genomic 7-mers across four related yeast species. Distributions of the percentage of 7-mers in each bin from *S.cerevisiae* which have identical k-mers in the corresponding bins from other, related yeast species. The 7-mers for each species were derived from the unannotated intergenic regions and the fold enrichment scores were calculated based on the Ak-1 method.

Comparative analysis of k-mers derived from the unannotated intergenic regions of the genome using our Ak-1 method consistently showed a normal distribution that was slightly skewed to the right for all three values of k tested (Figure 5 and additional Figures S9 and S17). However, for an identical comparative analysis of 9-mers with the C0/C1 method (Figure S21) we observed multimodal and not normal distributions (Figures S5 and S13).

While the difference in modality observed using the two different methods may be explained as stated above, broad unimodal distributions could capture the longer range of fold enrichments scores that need be maintained for sequence elements derived from the intergenic regions. Since the C0/C1 method captures more repetitive DNA sequences, and also because repetitive sequences are associated with distinct genomic features with distinct copy numbers (e.g., k-mers from duplicated genes, k-mers from transposons), the multimodal distributions may arise from the distinct ranges of fold enrichment values typically associated with each of these classes.

#### Overrepresented k-mers preserved across yeast species

We also checked whether some of the k-mers detected as overrepresented in our analysis, and which represent known TF-binding motifs, were conserved in the three other yeast species, *S.bayanus, S.paradoxus* and *S.mikatae*. Interestingly, nine out of the top 50 overrepresented k-mers in *S.cerevisiae*, compiled after collapsing the top 10 k-mers (7<k<13), were also overrepresented in the genomes of the other three yeast species (Table S5).

## Discussion

In this study, our primary goals were to put forth a novel statistical background model to calculate k-mer enrichment and then to apply this model to the genomes of a set of related yeast species to gain new biological insights.

A suitable statistical background model to calculate “expected frequencies” of k-mers is essential for detecting over- or underrepresented DNA words [16]. A number of such null models exist against which over- and underrepresentation of k-mers can be assessed. Most notably, Markov models of different orders (l<k, where ‘l’ represents the order of the Markov model) have been used as background models in several studies [10], [11], [19]. Hampson et al. introduced another background model, dubbed the C0/C1 method, which uses frequencies of all the single base mismatches of a k-mer to arrive at an estimated count for that k-mer [16]. In that paper, the authors compared their method against Markov models of varying orders and concluded that the C0/C1 model and Markov models of orders in the vicinity of k/2 (l = k/2) can serve as optimal models to identify over- and underrepresented k-mers. However, as noted in the paper itself, and as is the case with Markov models, it suffers from some amount of AT-rich compositional bias.

Our novel background model, i.e., the Ak-1 method, introduced here can serve as a useful null model against which k-mers can be assessed for their fold enrichment. Comparison of the top 20 k-mers arising from the average (k-1) mer method to the ones from the C0/C1 method and the Markov models shows that there is a significant amount of redundancy between them in their ranking of k-mers. Thus, some of the overrepresented k-mers detected by existing ‘optimal’ background models were also identified to be top ranking by our method. However, our method identifies additional over-represented k-mers not detected by any of the other methods. For example (Table 1), there are 10 k-mers in the top 20 list from the Ak-1 method that are not found in the top 20 lists from any of the other models. Also, our method compares favorably alongside the Markov model order 4, Z-score sorted protocol in reporting the least number of repeat-rich elements among the top ranking k-mers.

The same comparisons show that the Ak-1 method detects more GC-rich DNA strings as compared to the other methods. While a potential drawback in our method might be that it identifies too many such GC-rich strings, it nevertheless completely eliminates the AT-rich bias given that the genomic sequence from which the k-mers were derived had an AT content of up to 61.2%. Thus, our method may be the optimal one to use in scenarios that require the identification of repeat-poor, GC-rich strings that are over-represented in AT-rich input sequences and vice versa.

Our method outperforms existing over-representation detection methods in identifying known biologically relevant k-mers. This is supported by the comparative analysis of top 20 8-mers derived from the six different methods. Notably, four of the nine 8-mers uniquely detected by the Ak-1 method represent eight TF binding motifs, some of which are overlapping. Moreover, our method includes more TF binding motifs in the enrichment score based top 20 rank list as compared to other methods.

We also report for the first time the distribution of k-mer frequencies and of the fraction of over-represented k-mers across a biological meaningful range of values of k (4≤k≤16). Two important observations should be highlighted: (a) the fraction of genomic k-mers that are statistically overrepresented in all four yeast species analyzed decreases almost exponentially for k values between 9 and 12. Interestingly, a small, but significant, number of k-mers remain (up to k = 16) over-represented irrespective of the method used for fold enrichment score calculation. Since our method captures fewer low complexity DNA containing strings, we hypothesize that a majority of these may represent longer elements that are functionally important. While some of these larger elements may have arisen due to recent chromosomal duplications, it seems likely that at least a subset of such elements serve some regulatory or structural function. (b) The overrepresentation calculation from our Ak-1 method identified almost all of the observed genomic k-mers as overrepresented up to and including k = 9. This artifact could be due to the fact that the over-representation calculation for any given k-mer by our method is based on the number of occurrences of its corresponding (k-1) mers. And also because the “space” of novel k-mers explored for each increment of k increases approximately by a factor of 10, up to and including k = 9, when the theoretical and observed k-mer counts begin to markedly deviate.

Thus, while our method can be used to rank k-mers based on enrichment for all values of k, we note a potential shortcoming of the same: our average (k-1) mer method may not be the optimal one to detect over-represented k-mers until a value of k is reached where the observed number of k-mers begins to diverge from the theoretically calculated ones.

Both theoretical and empirical distributions of k-mer frequencies or k-mer spectra in genomic sequences have been previously studied. In these studies, the authors have plotted the number of k-mers (for a given value of k) against the frequencies of their occurrence in the genomic region to draw the k-mer spectra. Csuros et al. analyzed the k-mer spectra from sixty diverse genomes and suggested that a Double Pareto-lognormal distribution (DPLN) best captures the multimodal spectra and also accounts for the heavy right tail of the graph associated with over-abundant k-mers [4]. In a subsequent independent study, Chor et al. analyzed k-mer spectra for more than 100 genomes and reported unimodal genomic k-mer spectra for most species. The exceptions were the tetrapods, including all mammals, whose genomes and most genomic sub-regions exhibited multimodal k-mer spectra. They also underscored the suitability of low order (l<k/2) Markov models in recapitulating the multimodal spectra and heavy tails of such distributions, refuting the DPLN model [3]. The 8-mer genomic spectra reported for *S.cerevisiae* by Chor et al. is typically unimodal with the mode at a relatively low k-mer copy number, at around 200.

In order to glean more insights into the nature of k-mer spectra, we decided to analyze comparative k-mer spectra to learn more about the modality of the resulting distributions. Specifically, we addressed the question “how many k-mers in the genomic regions of *S.cerevisiae* exhibit similar fold enrichment scores across related yeast species and what is the modality of the distribution arising from the comparative analysis?”

Interestingly, the analysis of 8-mers derived from the whole genome of the four yeast species showed a positively skewed normal distribution for some combinations of species. However, for comparisons involving only *S.paradoxus* and *S.bayanus*, the resulting distributions were multimodal. This implies that there does not seem to be additional evolutionary selection pressure at both the high and low ends of the fold enrichment score ranked k-mers, at least at the genome scale level.

We were unable to observe any consistent trends across neighboring values of k or across the fold enrichment calculation method used for k-mers derived from either the regions 1 kb up- and downstream of all ORFs or for the intergenic regions. However, the multimodal distributions observed in some of these graphs suggests the existence of distinct classes of k-mers (reflecting diverse functional or structural elements), whose relative enrichment in the genome is regulated by evolution to some extent. We suspect that major changes in the relative abundances of these classes of k-mers may have deleterious effects for the organism.

## Conclusions

We have presented a novel statistical background model, the Ak-1 method, that can be used for the optimal identification of repeat-poor, GC-rich strings that are over-represented in AT-rich input sequences and vice versa. Also, the Ak-1 method includes more biologically relevant k-mers in the enrichment score based top ranking lists as compared to existing methods. Our analysis of overrepresented genomic k-mers (4≤k≤16) from four closely related yeast species revealed that the fraction of overrepresented DNA words decreases linearly as k increases, and this is most strikingly observed as the DNA word length goes from k = 9 to k = 12. We also found that a significant number of overabundant k-mers exists at higher values of k (k = 16). Additionally, the comparative analysis of k-mer fold enrichment scores revealed a mixture of unimodal and multimodal spectra for the different genomic sub-regions analyzed. This contrasts sharply with the unimodal k-mer spectra observed for only the *S.cerevisiae* genome [3].

We believe that similar comparative analyses of DNA word frequencies between closely related vertebrate species will shed several new insights into k-mer evolution in human. Currently, we are in the process of extrapolating our analysis to include several other groups of related species such as human and mouse. Since our Ak-1 method can be used to identify a significant number of over-represented sequence motifs missed by other methods, it should prove an invaluable addition to the current catalog of motif-identifying algorithms.

## Supporting Information

Figure S1 **The percentage of overrepresented genomic k-mers (4≤k≤16) in each of the four related yeast species.** Fold enrichment scores and Z-scores for calculating overrepresentation statistics were determined using the Ak-1 method. For each value of k, the appropriate Z-score cut-off for p<0.05 was determined after applying the Bonferroni correction for multiple testing.
(PPTX)

Figure S2 **Distributions of the percentage of identical 7-mers between each bin from** ***S.cerevisiae*** **and the corresponding bins from other, related yeast species.** The 7-mers for each species were derived from the complete genome and the fold enrichment scores were calculated based on the C0/C1 method.
(PPTX)

Figure S3 **Distributions of the percentage of identical 7-mers between each bin from** ***S.cerevisiae*** **and the corresponding bins from other, related yeast species.** The 7-mers for each species were derived from the regions 1 kb upstream of annotated ORFs and the fold enrichment scores were calculated based on the C0/C1 method.
(PPTX)

Figure S4 **Distributions of the percentage of identical 7-mers between each bin from** ***S.cerevisiae*** **and the corresponding bins from other, related yeast species.** The 7-mers for each species were derived from the regions 1 kb downstream of annotated ORFs and the fold enrichment scores were calculated based on the C0/C1 method.
(PPTX)

Figure S5 **Distributions of the percentage of identical 7-mers between each bin from** ***S.cerevisiae*** **and the corresponding bins from other, related yeast species.** The 7-mers for each species were derived from the unannotated intergenic regions and the fold enrichment scores were calculated based on the C0/C1 method.
(PPTX)

Figure S6 **Distributions of the percentage of identical 8-mers between each bin from** ***S.cerevisiae*** **and the corresponding bins from other, related yeast species.** The 8-mers for each species were derived from the complete genome and the fold enrichment scores were calculated based on the Ak-1 method
(PPTX)

Figure S7 **Distributions of the percentage of identical 8-mers between each bin from** ***S.cerevisiae*** **and the corresponding bins from other, related yeast species.** The 8-mers for each species were derived from the regions 1 kb upstream of annotated ORFs and the fold enrichment scores were calculated based on the Ak-1 method.
(PPTX)

Figure S8 **Distributions of the percentage of identical 8-mers between each bin from** ***S.cerevisiae*** **and the corresponding bins from other, related yeast species.** The 8-mers for each species were derived from the regions 1 kb downstream of annotated ORFs and the fold enrichment scores were calculated based on the Ak-1 method.
(PPTX)

Figure S9 **Distributions of the percentage of identical 8-mers between each bin from** ***S.cerevisiae*** **and the corresponding bins from other, related yeast species.** The 8-mers for each species were derived from the unannotated intergenic regions and the fold enrichment scores were calculated based on the Ak-1 method.
(PPTX)

Figure S10 **Distributions of the percentage of identical 8-mers between each bin from** ***S.cerevisiae*** **and the corresponding bins from other, related yeast species.** The 8-mers for each species were derived from the complete genome and the fold enrichment scores were calculated based on the C0/C1 method.
(PPTX)

Figure S11 **Distributions of the percentage of identical 8-mers between each bin from** ***S.cerevisiae*** **and the corresponding bins from other, related yeast species.** The 8-mers for each species were derived from the regions 1 kb upstream of annotated ORFs and the fold enrichment scores were calculated based on the C0/C1 method.
(PPTX)

Figure S12 **Distributions of the percentage of identical 8-mers between each bin from** ***S.cerevisiae*** **and the corresponding bins from other, related yeast species.** The 8-mers for each species were derived from the regions 1 kb downstream of annotated ORFs and the fold enrichment scores were calculated based on the C0/C1 method.
(PPTX)

Figure S13 **Distributions of the percentage of identical 8-mers between each bin from** ***S.cerevisiae*** **and the corresponding bins from other, related yeast species.** The 8-mers for each species were derived from the unannotated intergenic regions and the fold enrichment scores were calculated based on the C0/C1 method.
(PPTX)

Figure S14 **Distributions of the percentage of identical 9-mers between each bin from** ***S.cerevisiae*** **and the corresponding bins from other, related yeast species.** The 9-mers for each species were derived from the complete genome and the fold enrichment scores were calculated based on the Ak-1 method.
(PPTX)

Figure S15 **Distributions of the percentage of identical 9-mers between each bin from** ***S.cerevisiae*** **and the corresponding bins from other, related yeast species.** The 9-mers for each species were derived from the regions 1 kb upstream of annotated ORFs and the fold enrichment scores were calculated based on the Ak-1 method.
(PPTX)

Figure S16 **Distributions of the percentage of identical 9-mers between each bin from** ***S.cerevisiae*** **and the corresponding bins from other, related yeast species.** The 9-mers for each species were derived from the regions 1 kb downstream of annotated ORFs and the fold enrichment scores were calculated based on the Ak-1 method.
(PPTX)

Figure S17 **Distributions of the percentage of identical 9-mers between each bin from** ***S.cerevisiae*** **and the corresponding bins from other, related yeast species.** The 9-mers for each species were derived from the unannotated intergenic regions and the fold enrichment scores were calculated based on the Ak-1 method.
(PPTX)

Figure S18 **Distributions of the percentage of identical 9-mers between each bin from** ***S.cerevisiae*** **and the corresponding bins from other, related yeast species.** The 9-mers for each species were derived from the complete genome and the fold enrichment scores were calculated based on the C0/C1 method.
(PPTX)

Figure S19 **Distributions of the percentage of identical 9-mers between each bin from** ***S.cerevisiae*** **and the corresponding bins from other, related yeast species.** The 9-mers for each species were derived from the regions 1 kb upstream of annotated ORFs and the fold enrichment scores were calculated based on the C0/C1 method.
(PPTX)

Figure S20 **Distributions of the percentage of identical 9-mers between each bin from** ***S.cerevisiae*** **and the corresponding bins from other, related yeast species.** The 9-mers for each species were derived from the regions 1 kb downstream of annotated ORFs and the fold enrichment scores were calculated based on the C0/C1 method.
(PPTX)

Figure S21 **Distributions of the percentage of identical 9-mers between each bin from** ***S.cerevisiae*** **and the corresponding bins from other, related yeast species.** The 9-mers for each species were derived from the unannotated intergenic regions and the fold enrichment scores were calculated based on the C0/C1 method.
(PPTX)

Table S1 **Comparison of the top 500 8-mers calculated using six different methods.** 8-mers derived from the region 500 bases upstream of all yeast ORFs were ranked by their relative abundance as calculated by several background models. The top 20 8-mers ranked by decreasing fold enrichment scores from (a) our novel background model, the “Ak-1 method” were compared to those from the previously described C0/C1 method (b), from Markov models of order 4 sorted by Z-scores (c), from Markov models of order 4 sorted by Binomial probabilities (d), from Markov models of order 3 ranked by Z-scores (e) and, Markov models of order 3 ranked by binomial probabilities (f). The list of 500 8-mers derived by applying our method was chosen as the reference against which the other lists were compared. 8-mers in each list that are identical to the ones in the reference list are marked with a superscript number, which shows its rank in the reference column (a). The average GC content of each list is shown in the bottom row of the table, followed by the percentage of k-mers each method shares with the Ak-1 method.
(DOCX)

Table S2 **Comparison of the top 20 8-mers calculated using six different methods as to their matches with transcription factor (TF) binding motifs in Yeastract.** 8-mers derived from the region 500 bases upstream of all yeast ORFs were ranked by their relative abundance as calculated by several background models. The top 20 8-mers ranked by decreasing fold enrichment scores from (a) our novel background model, the “Ak-1 method” were compared to those from the previously described C0/C1 method (b), from Markov models of order 4 sorted by Z-scores (c), from Markov models of order 4 sorted by Binomial probabilities (d), from Markov models of order 3 ranked by Z-scores (e) and, Markov models of order 3 ranked by binomial probabilities (f). The list of 20 8-mers derived by applying our method was chosen as the reference against which the other lists were compared. 8-mers in each list that are identical to the ones in the reference list are marked with a superscript number which shows its rank in the reference column (a), while repeat-rich elements are shown in red. The nine 8-mers identified uniquely by our method are highlighted in bold (a). The actual TF-binding motif matching the 8-mer along with the name of the TF from Yeastract are shown right next to each column of 8-mers. Both ‘Yeastract binding site inside of the inserted motif’ as well as ‘Inserted motif inside Yeastract binding site’ hits were considered.
(XLSX)

Table S3 **Comparison of the top 20 9-mers calculated using two different methods as to their matches with transcription factor (TF) binding motifs in Yeastract.** 9-mers derived from the region 500 bases upstream of all yeast ORFs were ranked by their relative abundance as calculated by different background models. The top 20 9-mers ranked by decreasing fold enrichment scores from (a) our novel background model, the “Ak-1 method” were compared to those from the previously described C0/C1 method (b). The list of 20 9-mers derived by applying our method was chosen as the reference against which the other list was compared. 9-mers in each list that are identical to the ones in the reference list are marked with a superscript number which shows its rank in the reference column (a), while repeat-rich elements are shown in red. The 9-mers identified uniquely by our method are highlighted in bold (a). The actual TF-binding motif matching the 9-mer and the name of the TF from Yeastract are shown next to each column of 9-mers. Both ‘Yeastract binding site inside of the inserted motif’ as well as ‘Inserted motif inside Yeastract binding site’ hits were considered.
(XLSX)

Table S4 **Comparison of the top 20 10-mers calculated using two different methods as to their matches with transcription factor (TF) binding motifs in Yeastract.** 10-mers derived from the region 500 bases upstream of all yeast ORFs were ranked by their relative abundance as calculated by different background models. The top 20 10-mers ranked by decreasing fold enrichment scores from (a) our novel background model, the “Ak-1 method” were compared to those from the previously described C0/C1 method (b). Repeat-rich elements are shown in red. The actual TF-binding motif matching the 10-mer and the name of the TF from Yeastract are shown next to each column of 10-mers. Both ‘Yeastract binding site inside of the inserted motif’ as well as ‘Inserted motif inside Yeastract binding site’ hits were considered.
(XLSX)

Table S5 Overrepresented k-mers (b) enriched in the 500 bp upstream regions of ORFs in the *S. cerevisiae* genome. The k-mer lengths (a) their presence in the overrepresented lists of the *S.bayanus*, *S.paradoxus* and *S.mikatae* genomes (c) along with their matches to known motifs from Yeastract (d).
(DOC)
