# Supplementary figures and images for: Comparative Analysis of DNA Word Abundances in Four Yeast Genomes Using a Novel Statistical Background Model
Source: PLoS One. 2013 Mar 5;8(3):e58038. doi: 10.1371/journal.pone.0058038 (PMC3589456; doi:10.1371/journal.pone.0058038)

## Slide 1
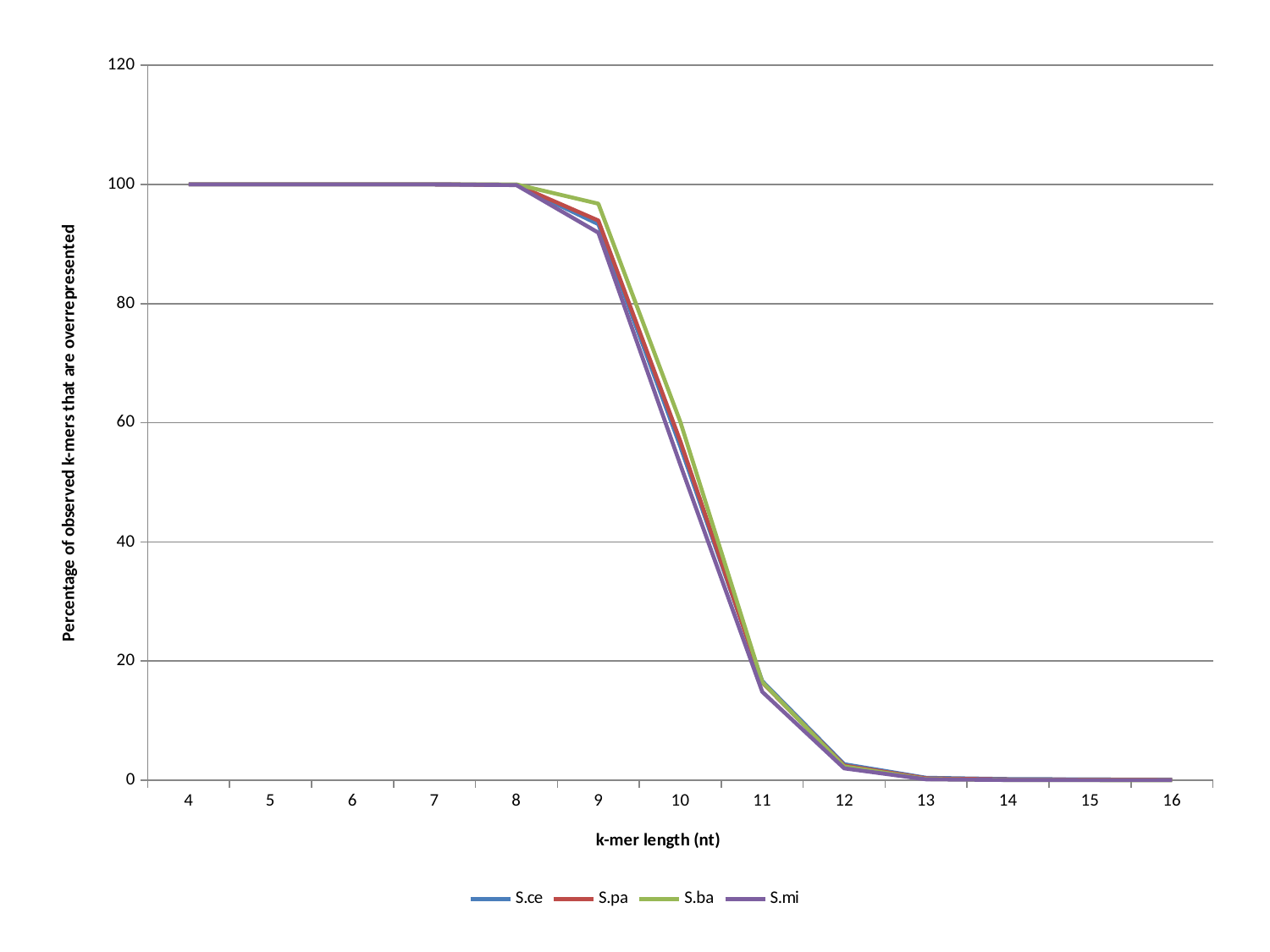

### Chart
| Category | | | | |
|---|---|---|---|---|
| 4 | 100.0 | 100.0 | 100.0 | 100.0 |
| 5 | 100.0 | 100.0 | 100.0 | 100.0 |
| 6 | 100.0 | 100.0 | 100.0 | 100.0 |
| 7 | 100.0 | 100.0 | 100.0 | 100.0 |
| 8 | 99.9267578125 | 99.9298095703125 | 100.0 | 99.88861083984375 |
| 9 | 93.32646677347982 | 93.94297747022607 | 96.76284790039062 | 91.90426416620254 |
| 10 | 56.0273546663391 | 56.91761757241218 | 60.16607579070858 | 52.97789466996036 |
| 11 | 16.652946965085818 | 16.404674889964518 | 16.484585507636133 | 14.84648317372261 |
| 12 | 2.6949938222841245 | 2.4717405341486374 | 2.3212004791427874 | 1.9971676303443626 |
| 13 | 0.41429579618580276 | 0.3238295313306431 | 0.20513327868051318 | 0.15312234755916035 |
| 14 | 0.20890933020008265 | 0.1351453019881763 | 0.045559980910503534 | 0.049054006915715445 |
| 15 | 0.1461427529512956 | 0.08672889762087738 | 0.01931431660692554 | 0.031631011039232826 |
| 16 | 0.11324566078573786 | 0.06635348155569862 | 0.01231621919050905 | 0.026562461557850692 |

Supplement: Figure S1 — The percentage of overrepresented genomic k-mers (4≤k≤16) in each of the four related yeast species. Fold enrichment scores and Z-scores for calculating overrepresentation statistics were determined using the Ak-1 method. For each value of k, the appropriate Z-score cut-off for p<0.05 was determined after applying the Bonferroni correction for multiple testing. (PPTX) [file pone.0058038.s001.pptx]

## Slide 1
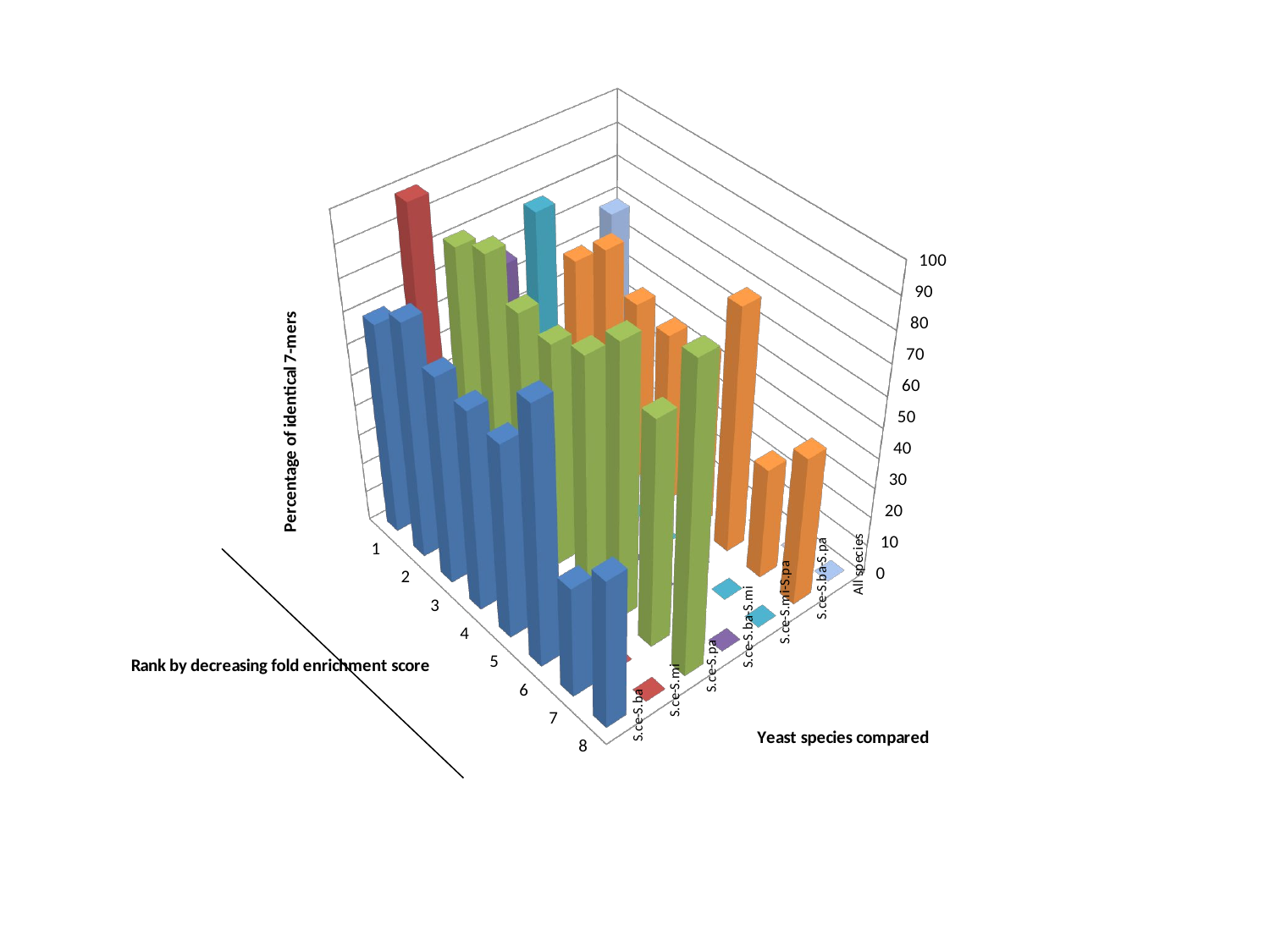

[unsupported chart]

Supplement: Figure S2 — Distributions of the percentage of identical 7-mers between each bin from S.cerevisiae and the corresponding bins from other, related yeast species. The 7-mers for each species were derived from the complete genome and the fold enrichment scores were calculated based on the C0/C1 method. (PPTX) [file pone.0058038.s002.pptx]

## Slide 1
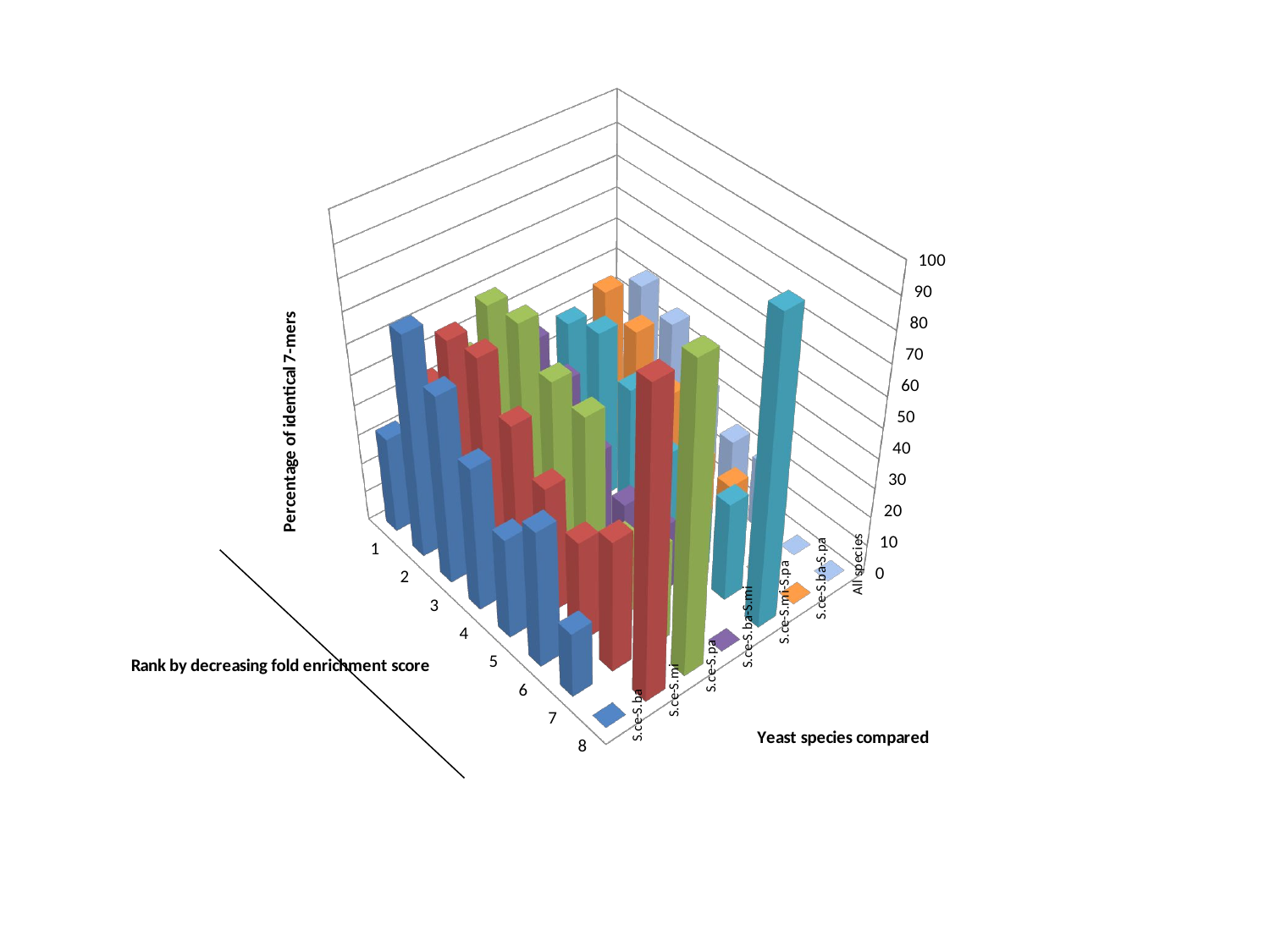

[unsupported chart]

Supplement: Figure S3 — Distributions of the percentage of identical 7-mers between each bin from S.cerevisiae and the corresponding bins from other, related yeast species. The 7-mers for each species were derived from the regions 1 kb upstream of annotated ORFs and the fold enrichment scores were calculated based on the C0/C1 method. (PPTX) [file pone.0058038.s003.pptx]

## Slide 1
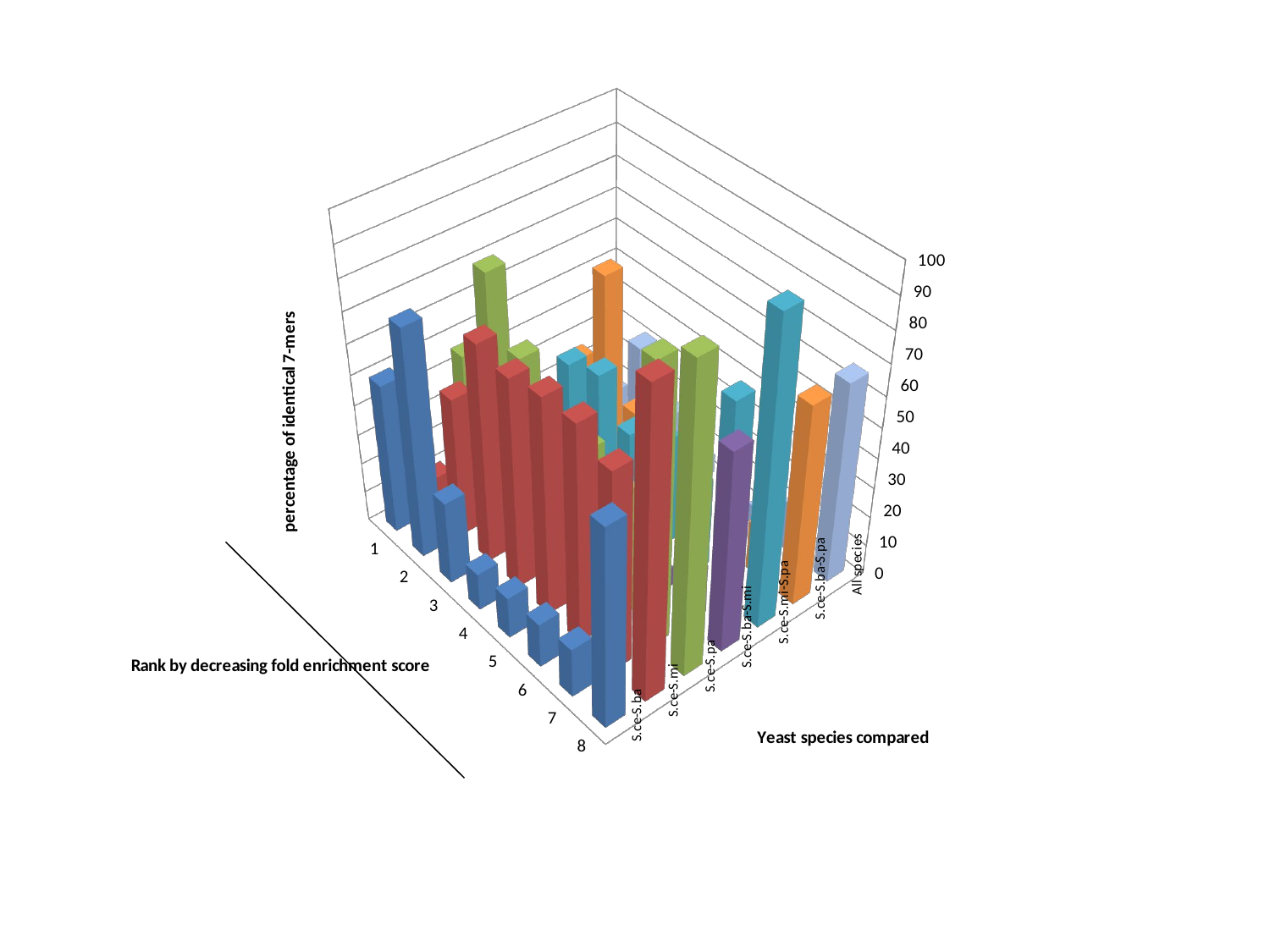

[unsupported chart]

Supplement: Figure S4 — Distributions of the percentage of identical 7-mers between each bin from S.cerevisiae and the corresponding bins from other, related yeast species. The 7-mers for each species were derived from the regions 1 kb downstream of annotated ORFs and the fold enrichment scores were calculated based on the C0/C1 method. (PPTX) [file pone.0058038.s004.pptx]

## Slide 1
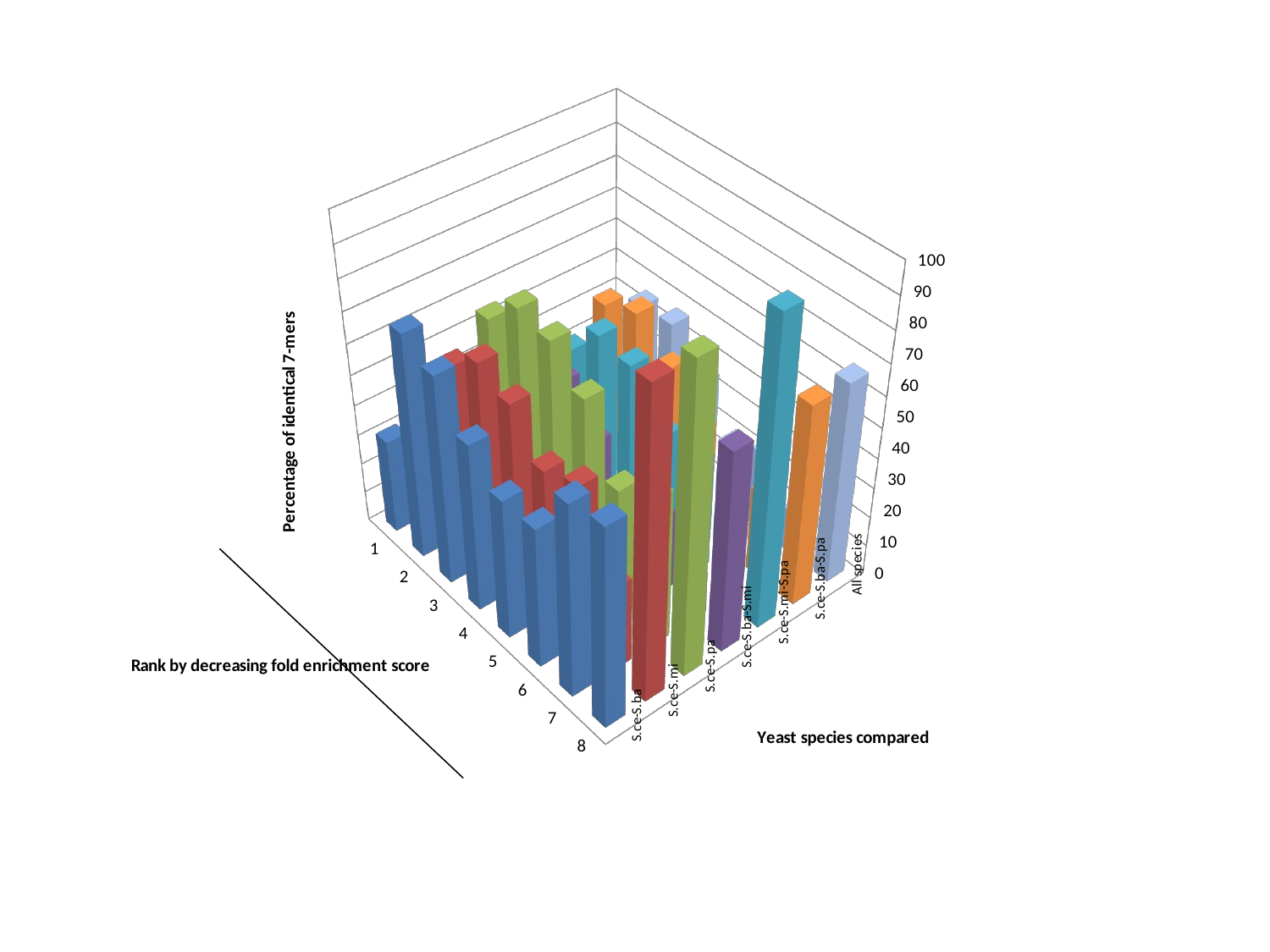

[unsupported chart]

Supplement: Figure S5 — Distributions of the percentage of identical 7-mers between each bin from S.cerevisiae and the corresponding bins from other, related yeast species. The 7-mers for each species were derived from the unannotated intergenic regions and the fold enrichment scores were calculated based on the C0/C1 method. (PPTX) [file pone.0058038.s005.pptx]

## Slide 1
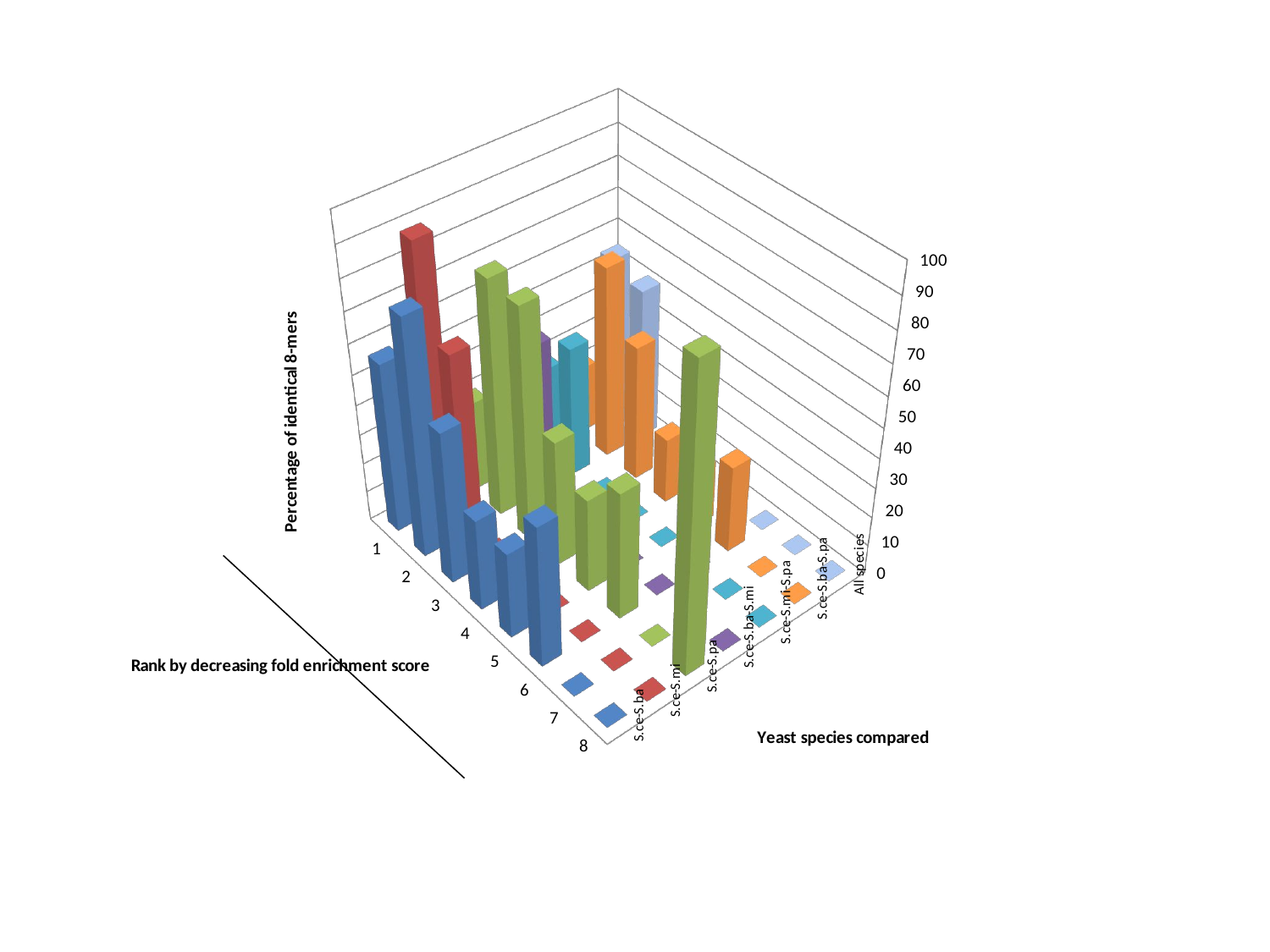

[unsupported chart]

Supplement: Figure S6 — Distributions of the percentage of identical 8-mers between each bin from S.cerevisiae and the corresponding bins from other, related yeast species. The 8-mers for each species were derived from the complete genome and the fold enrichment scores were calculated based on the Ak-1 method (PPTX) [file pone.0058038.s006.pptx]

## Slide 1
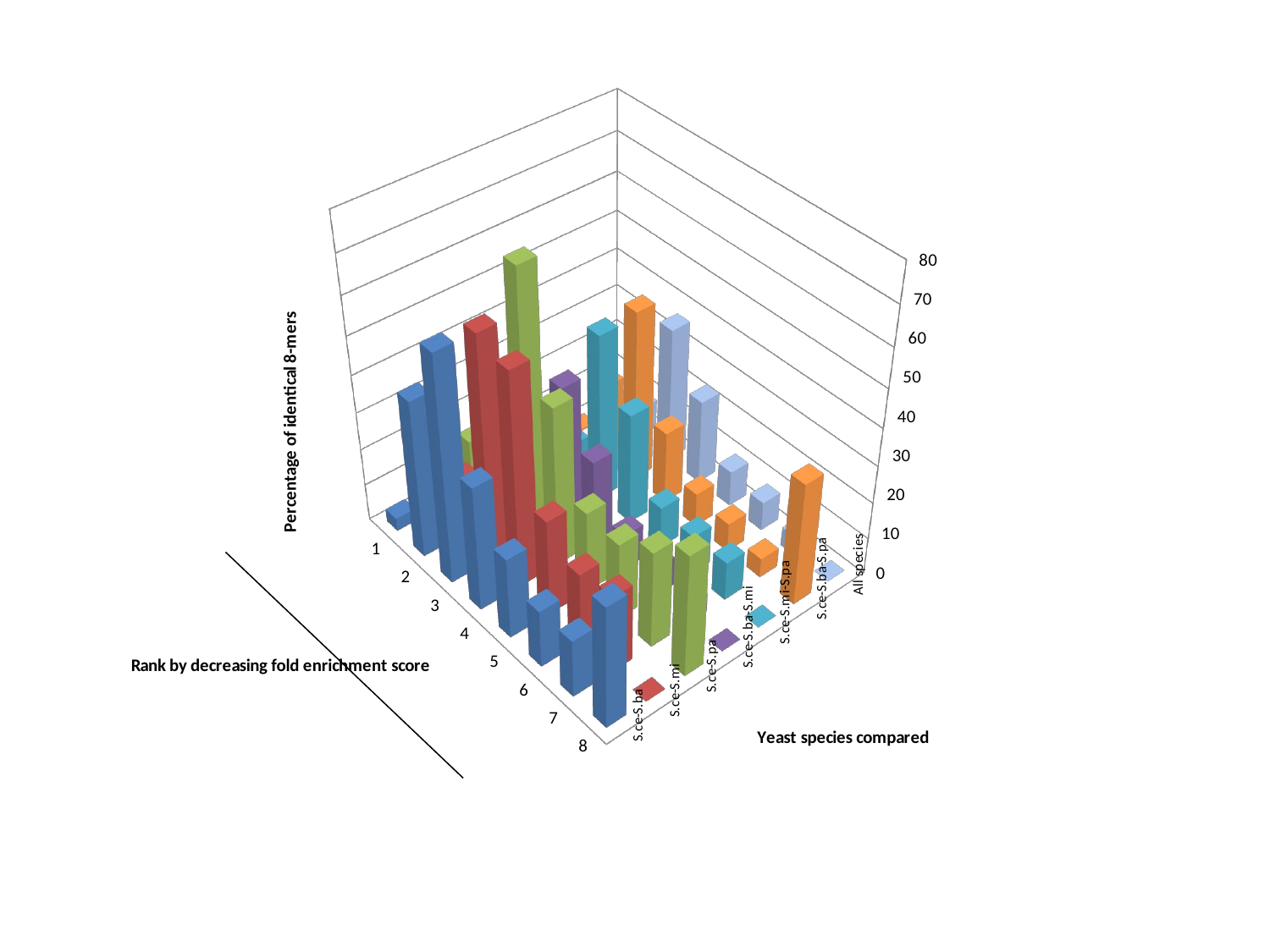

[unsupported chart]

Supplement: Figure S7 — Distributions of the percentage of identical 8-mers between each bin from S.cerevisiae and the corresponding bins from other, related yeast species. The 8-mers for each species were derived from the regions 1 kb upstream of annotated ORFs and the fold enrichment scores were calculated based on the Ak-1 method. (PPTX) [file pone.0058038.s007.pptx]

## Slide 1
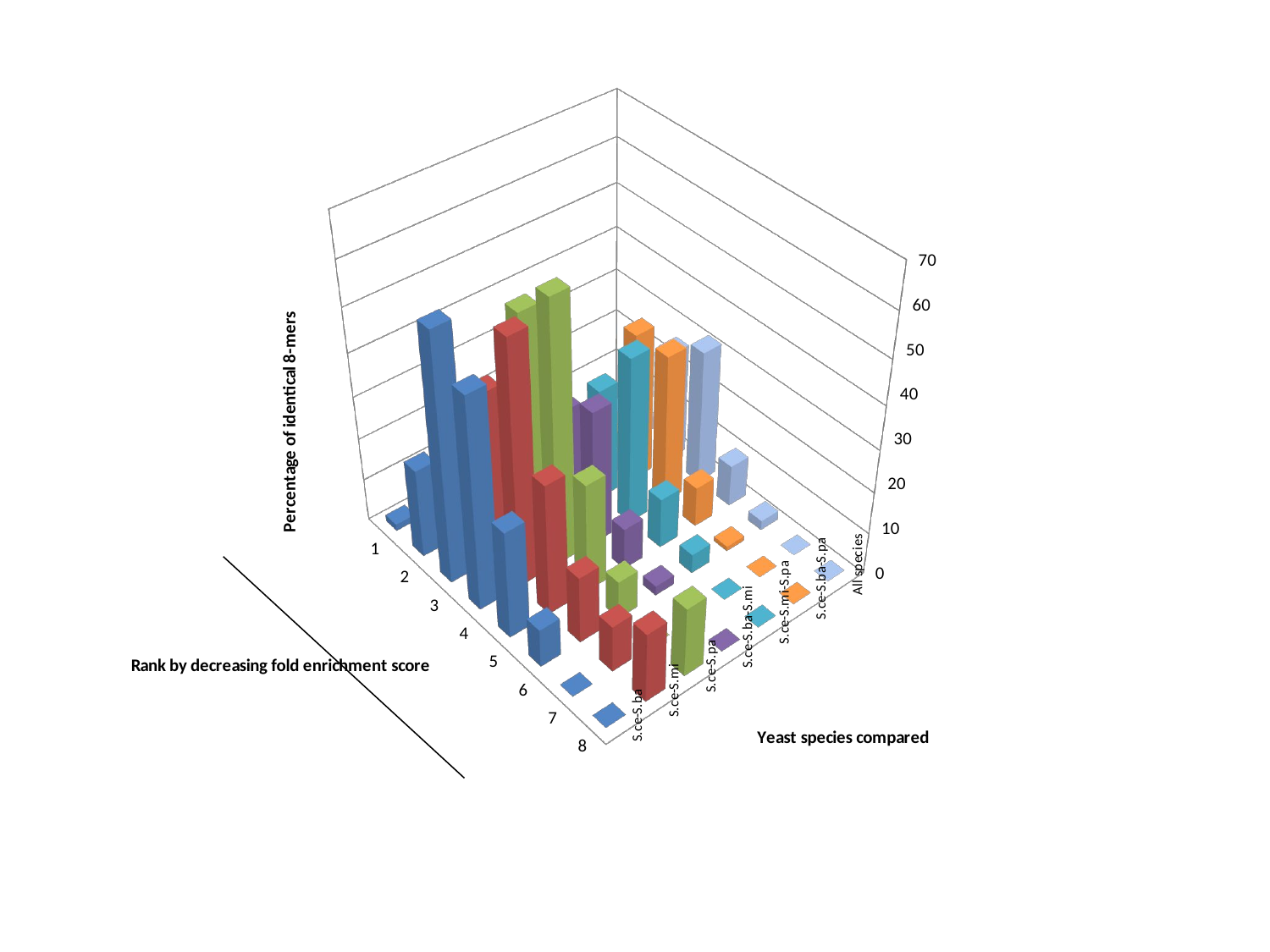

[unsupported chart]

Supplement: Figure S8 — Distributions of the percentage of identical 8-mers between each bin from S.cerevisiae and the corresponding bins from other, related yeast species. The 8-mers for each species were derived from the regions 1 kb downstream of annotated ORFs and the fold enrichment scores were calculated based on the Ak-1 method. (PPTX) [file pone.0058038.s008.pptx]

## Slide 1
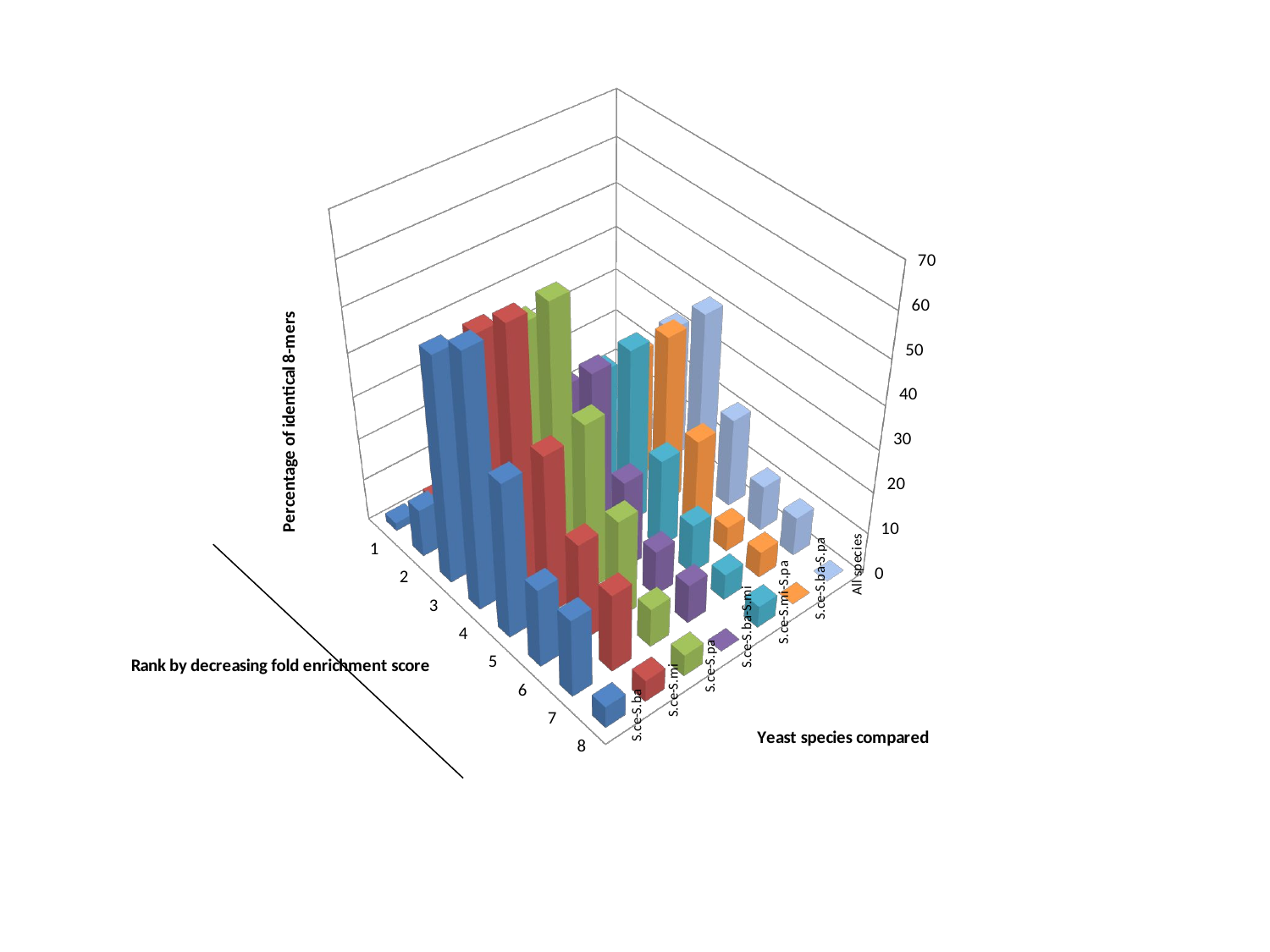

[unsupported chart]

Supplement: Figure S9 — Distributions of the percentage of identical 8-mers between each bin from S.cerevisiae and the corresponding bins from other, related yeast species. The 8-mers for each species were derived from the unannotated intergenic regions and the fold enrichment scores were calculated based on the Ak-1 method. (PPTX) [file pone.0058038.s009.pptx]

## Slide 1
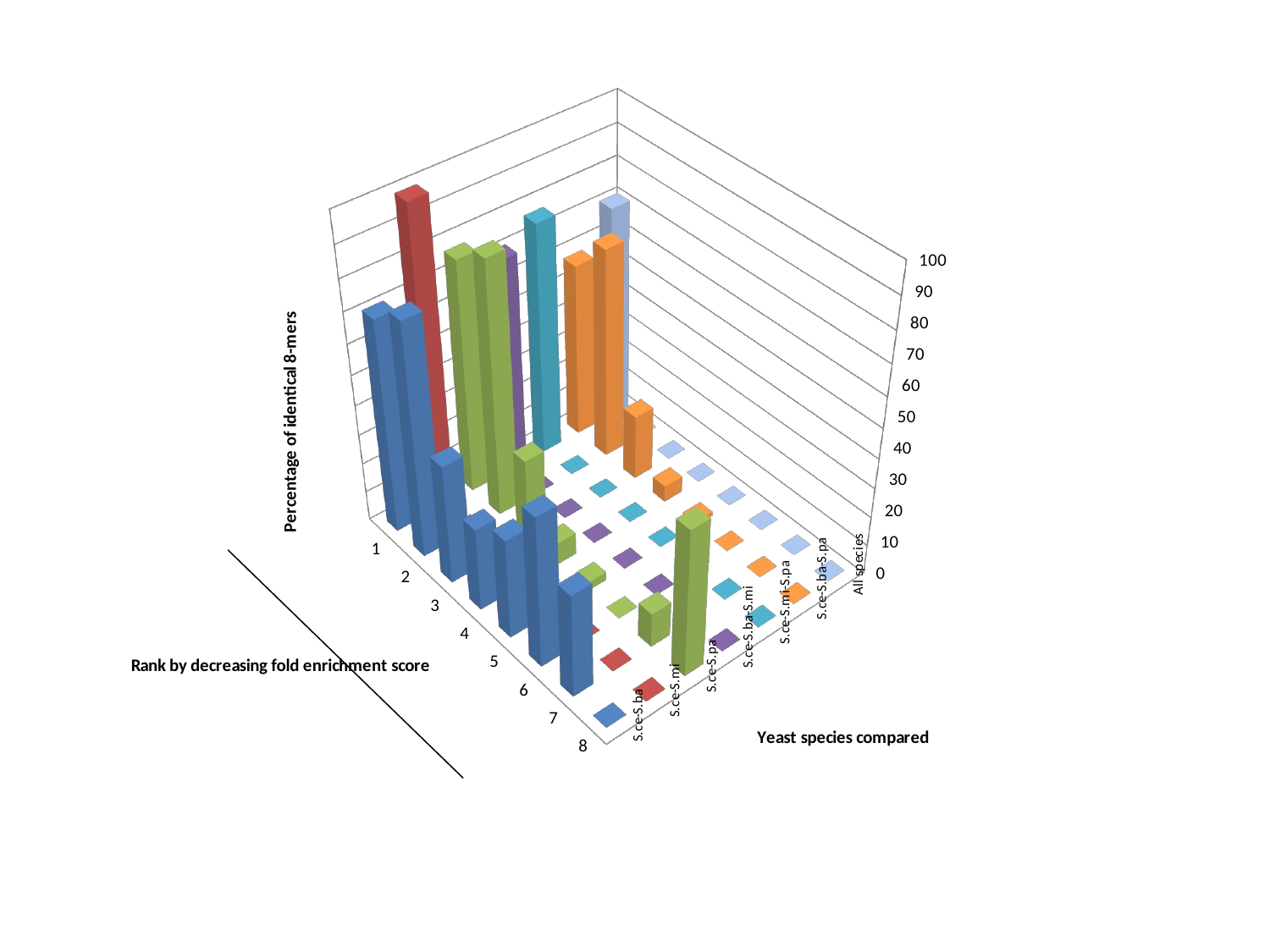

[unsupported chart]

Supplement: Figure S10 — Distributions of the percentage of identical 8-mers between each bin from S.cerevisiae and the corresponding bins from other, related yeast species. The 8-mers for each species were derived from the complete genome and the fold enrichment scores were calculated based on the C0/C1 method. (PPTX) [file pone.0058038.s010.pptx]

## Slide 1
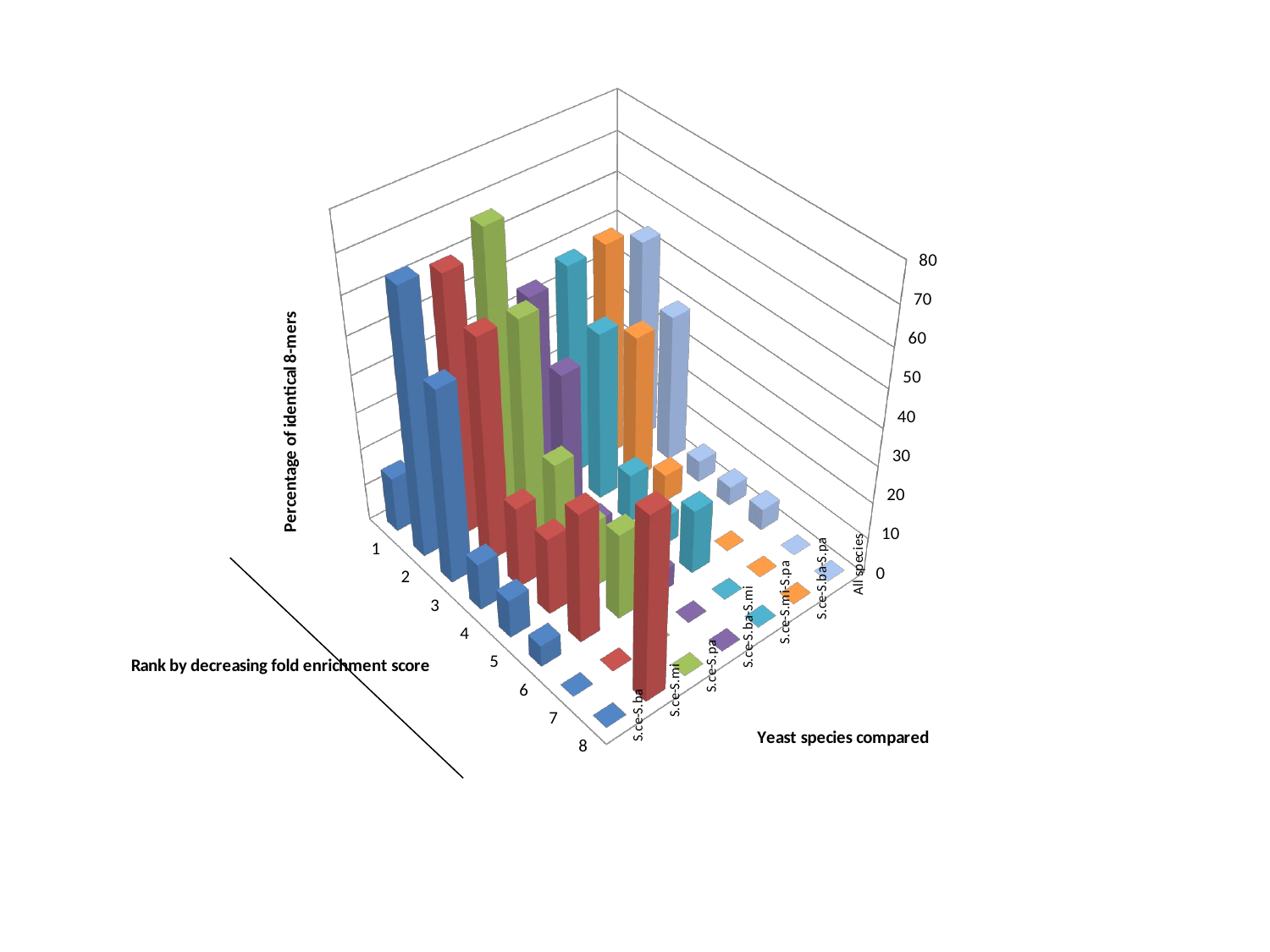

[unsupported chart]

Supplement: Figure S11 — Distributions of the percentage of identical 8-mers between each bin from S.cerevisiae and the corresponding bins from other, related yeast species. The 8-mers for each species were derived from the regions 1 kb upstream of annotated ORFs and the fold enrichment scores were calculated based on the C0/C1 method. (PPTX) [file pone.0058038.s011.pptx]

## Slide 1
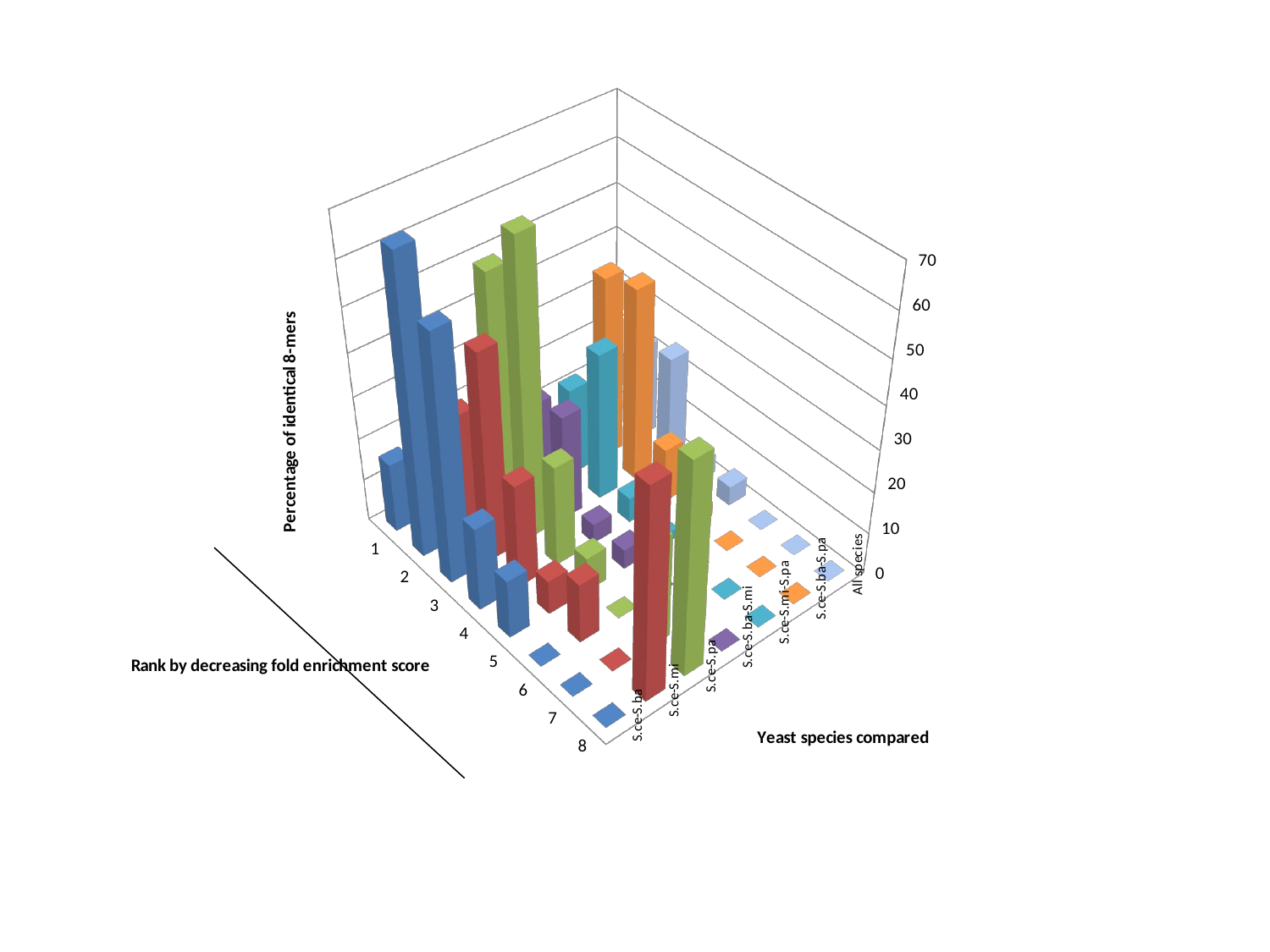

[unsupported chart]

Supplement: Figure S12 — Distributions of the percentage of identical 8-mers between each bin from S.cerevisiae and the corresponding bins from other, related yeast species. The 8-mers for each species were derived from the regions 1 kb downstream of annotated ORFs and the fold enrichment scores were calculated based on the C0/C1 method. (PPTX) [file pone.0058038.s012.pptx]

## Slide 1
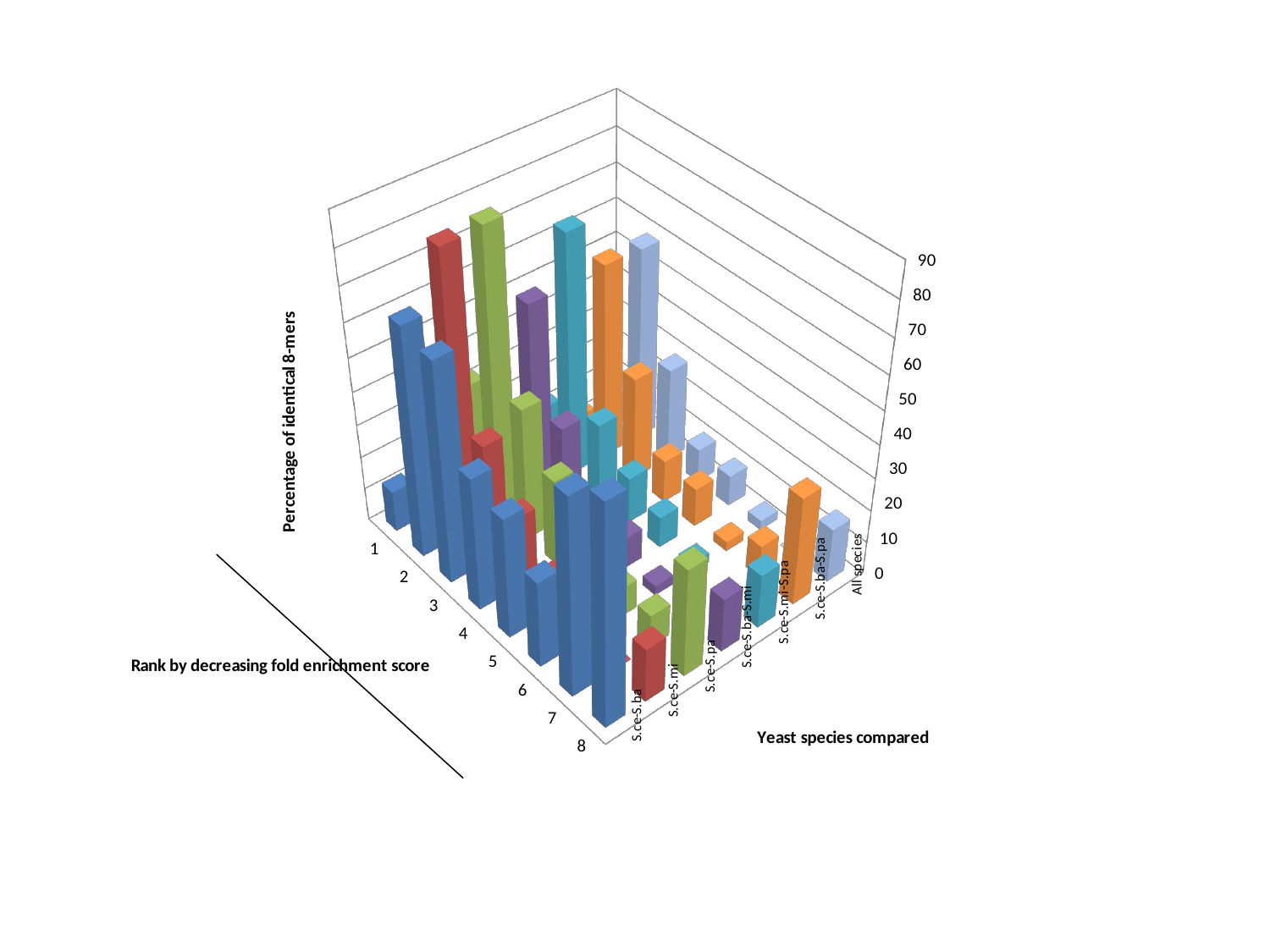

[unsupported chart]

Supplement: Figure S13 — Distributions of the percentage of identical 8-mers between each bin from S.cerevisiae and the corresponding bins from other, related yeast species. The 8-mers for each species were derived from the unannotated intergenic regions and the fold enrichment scores were calculated based on the C0/C1 method. (PPTX) [file pone.0058038.s013.pptx]

## Slide 1
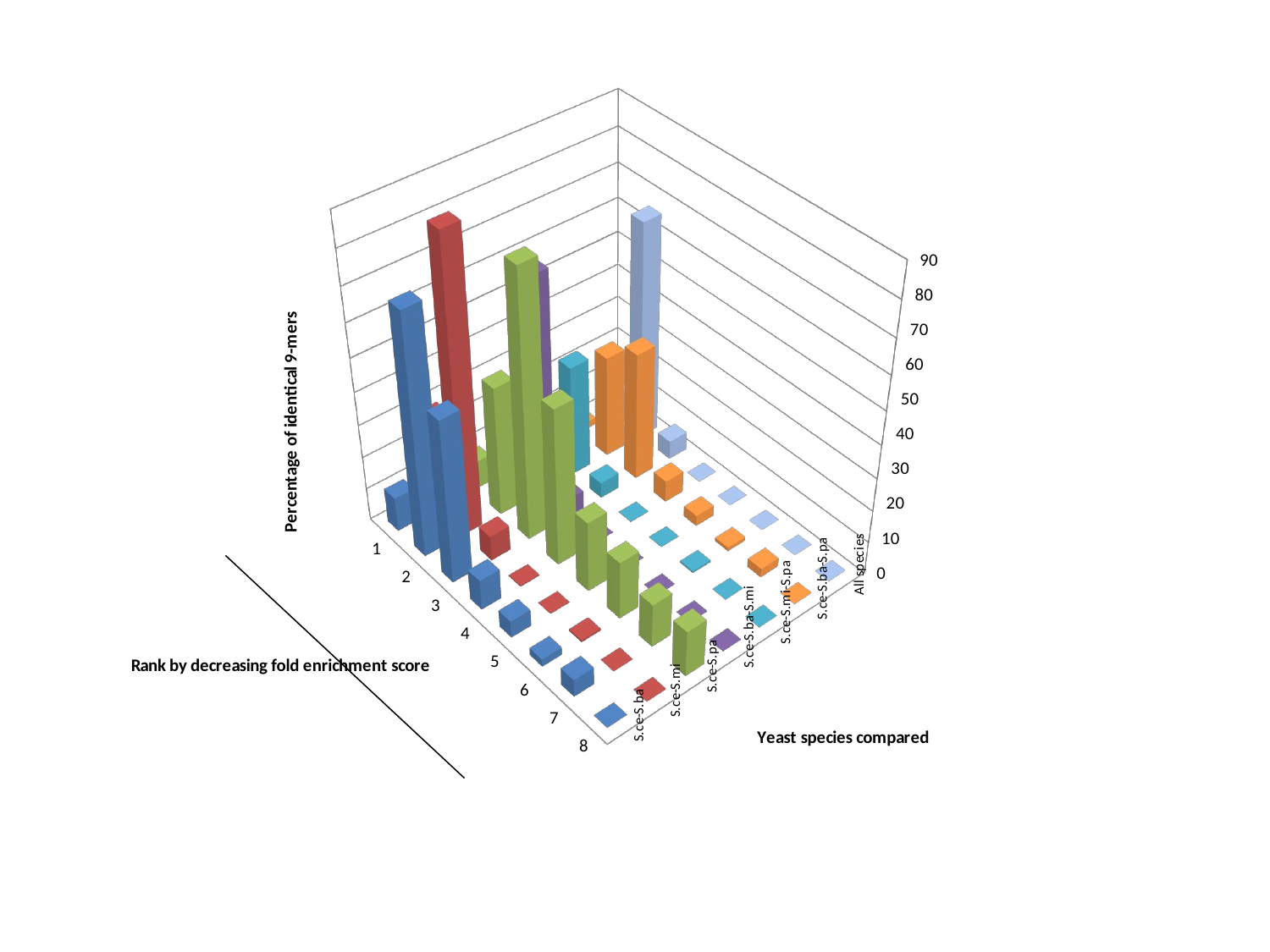

[unsupported chart]

Supplement: Figure S14 — Distributions of the percentage of identical 9-mers between each bin from S.cerevisiae and the corresponding bins from other, related yeast species. The 9-mers for each species were derived from the complete genome and the fold enrichment scores were calculated based on the Ak-1 method. (PPTX) [file pone.0058038.s014.pptx]

## Slide 1
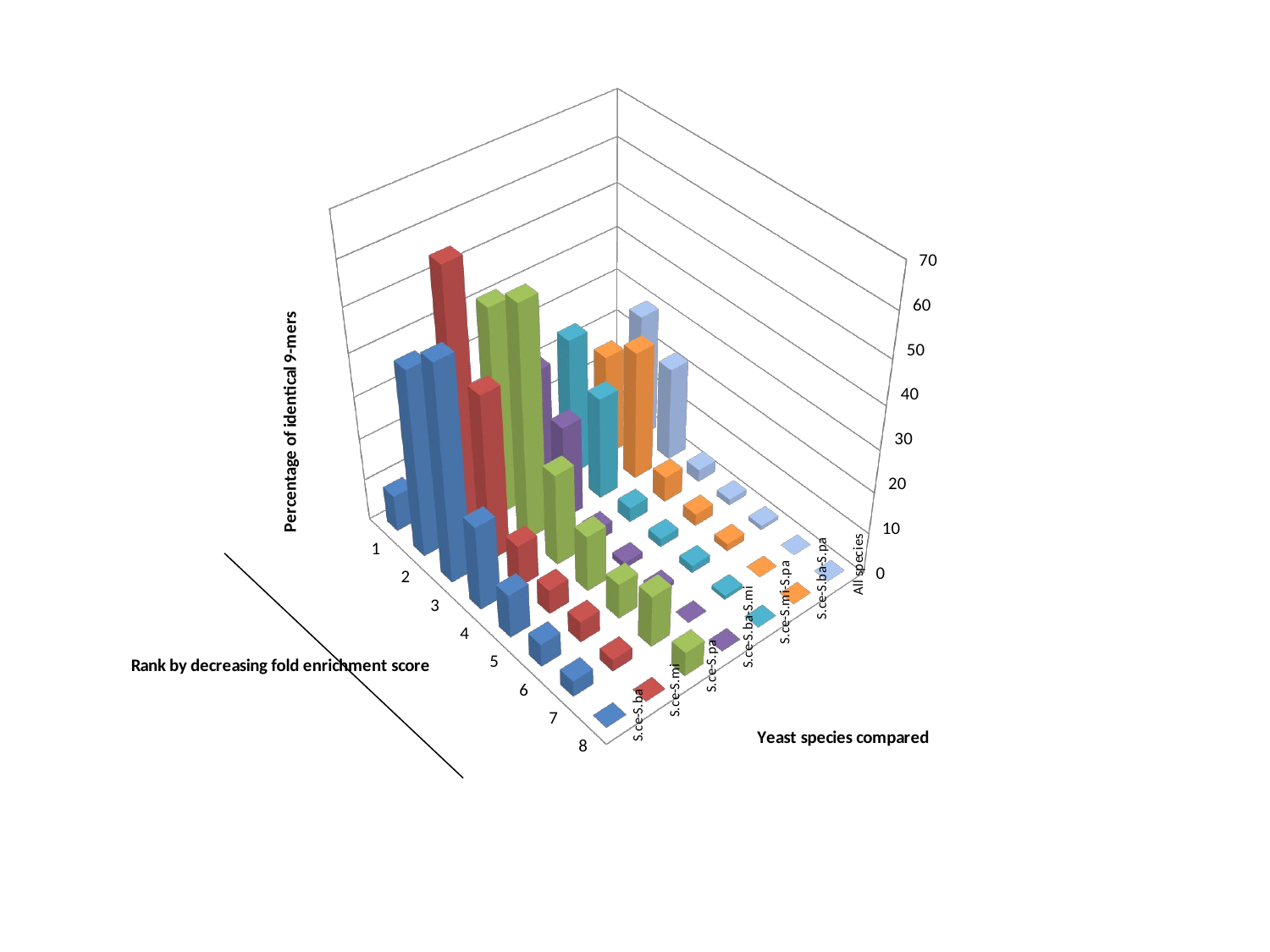

[unsupported chart]

Supplement: Figure S15 — Distributions of the percentage of identical 9-mers between each bin from S.cerevisiae and the corresponding bins from other, related yeast species. The 9-mers for each species were derived from the regions 1 kb upstream of annotated ORFs and the fold enrichment scores were calculated based on the Ak-1 method. (PPTX) [file pone.0058038.s015.pptx]

## Slide 1
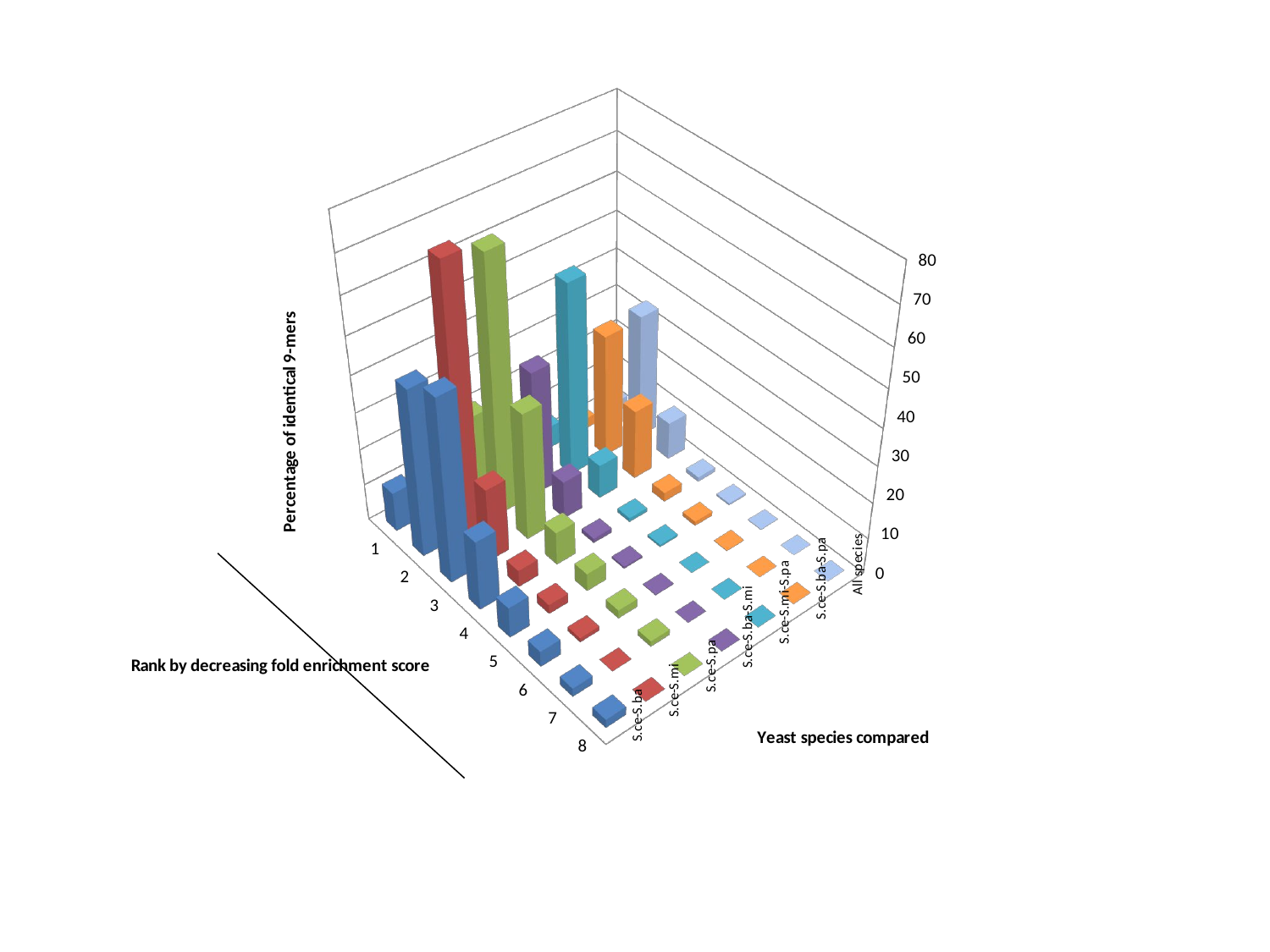

[unsupported chart]

Supplement: Figure S16 — Distributions of the percentage of identical 9-mers between each bin from S.cerevisiae and the corresponding bins from other, related yeast species. The 9-mers for each species were derived from the regions 1 kb downstream of annotated ORFs and the fold enrichment scores were calculated based on the Ak-1 method. (PPTX) [file pone.0058038.s016.pptx]

## Slide 1
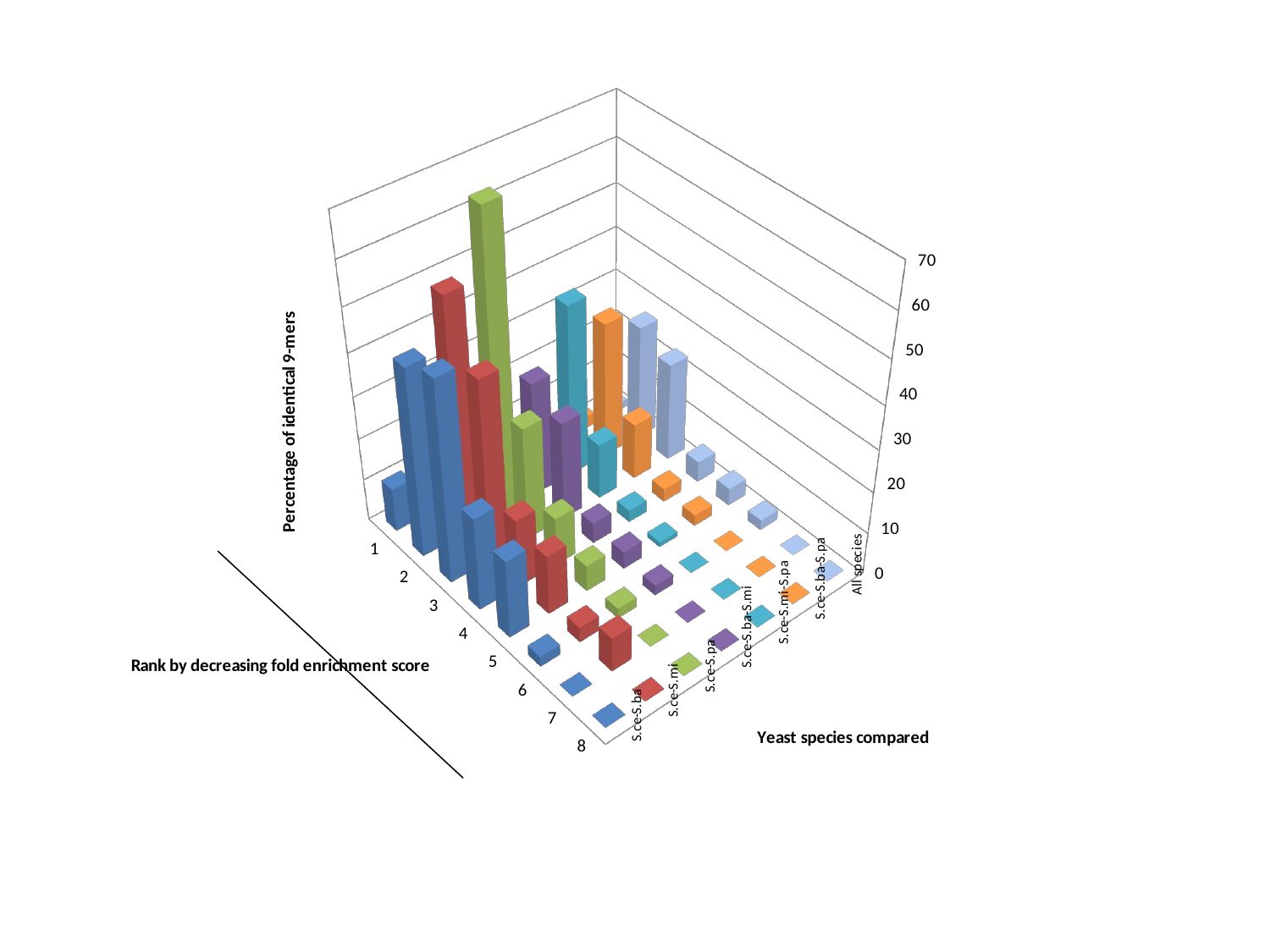

[unsupported chart]

Supplement: Figure S17 — Distributions of the percentage of identical 9-mers between each bin from S.cerevisiae and the corresponding bins from other, related yeast species. The 9-mers for each species were derived from the unannotated intergenic regions and the fold enrichment scores were calculated based on the Ak-1 method. (PPTX) [file pone.0058038.s017.pptx]

## Slide 1
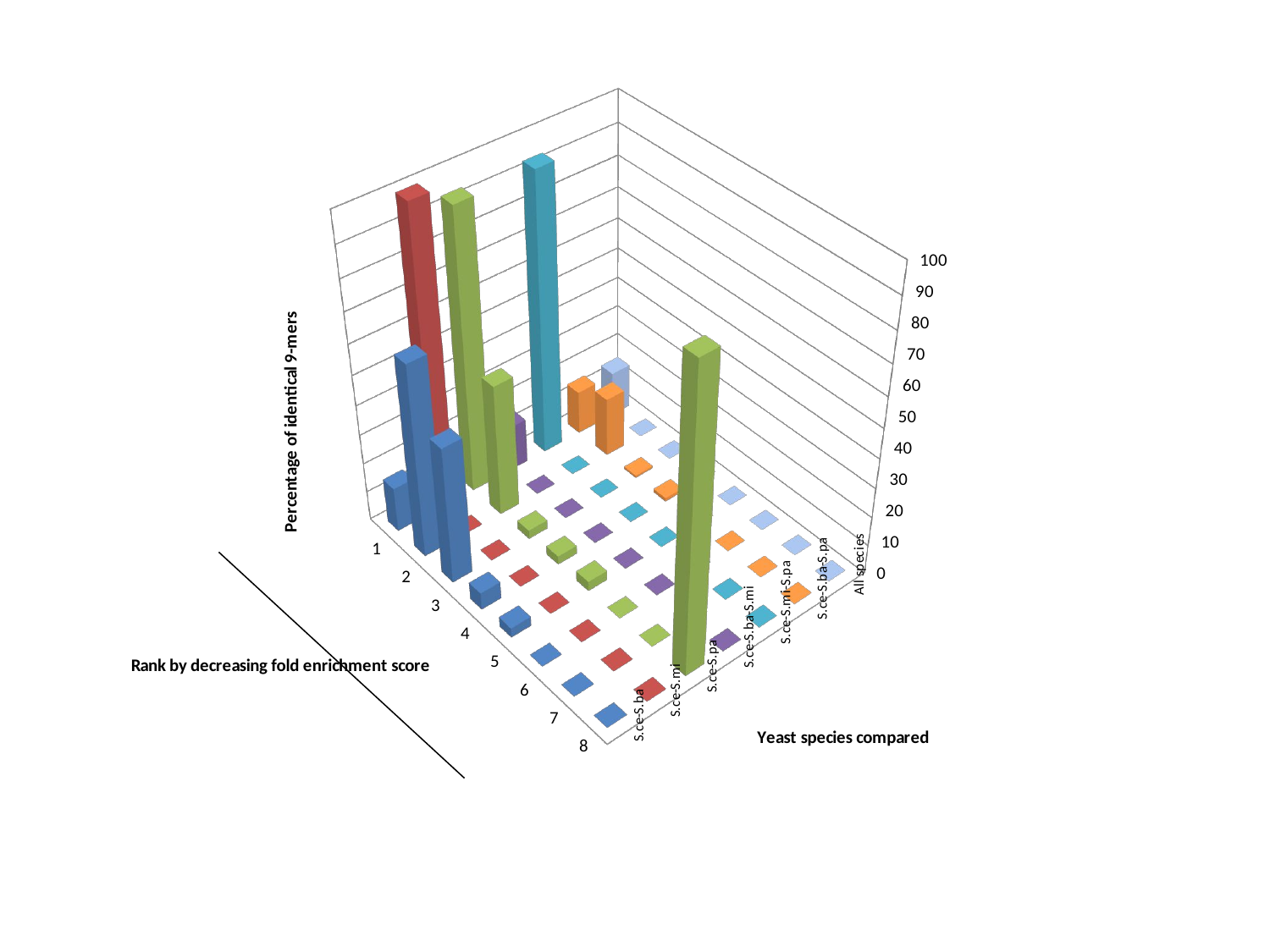

[unsupported chart]

Supplement: Figure S18 — Distributions of the percentage of identical 9-mers between each bin from S.cerevisiae and the corresponding bins from other, related yeast species. The 9-mers for each species were derived from the complete genome and the fold enrichment scores were calculated based on the C0/C1 method. (PPTX) [file pone.0058038.s018.pptx]

## Slide 1
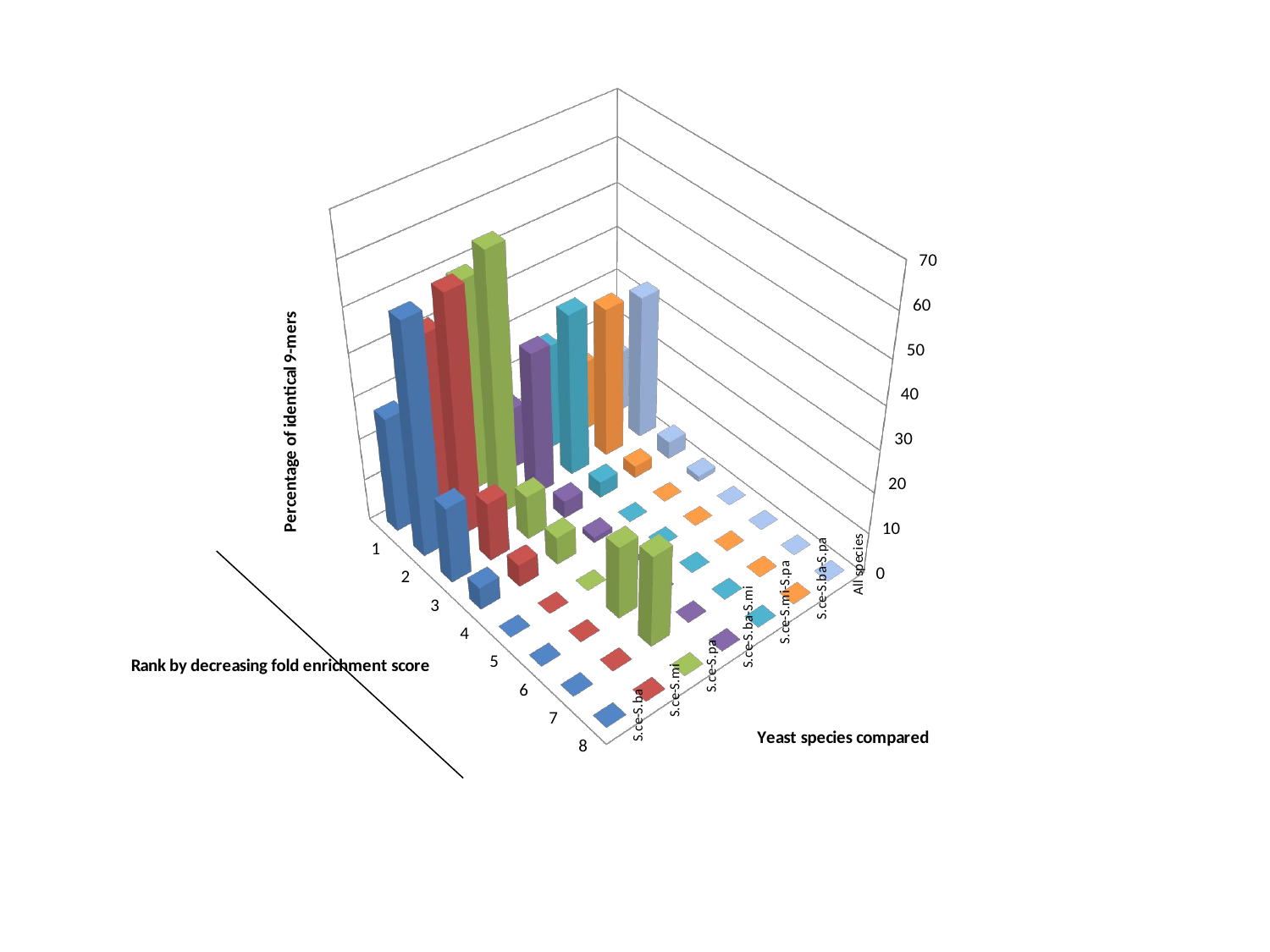

[unsupported chart]

Supplement: Figure S19 — Distributions of the percentage of identical 9-mers between each bin from S.cerevisiae and the corresponding bins from other, related yeast species. The 9-mers for each species were derived from the regions 1 kb upstream of annotated ORFs and the fold enrichment scores were calculated based on the C0/C1 method. (PPTX) [file pone.0058038.s019.pptx]

## Slide 1
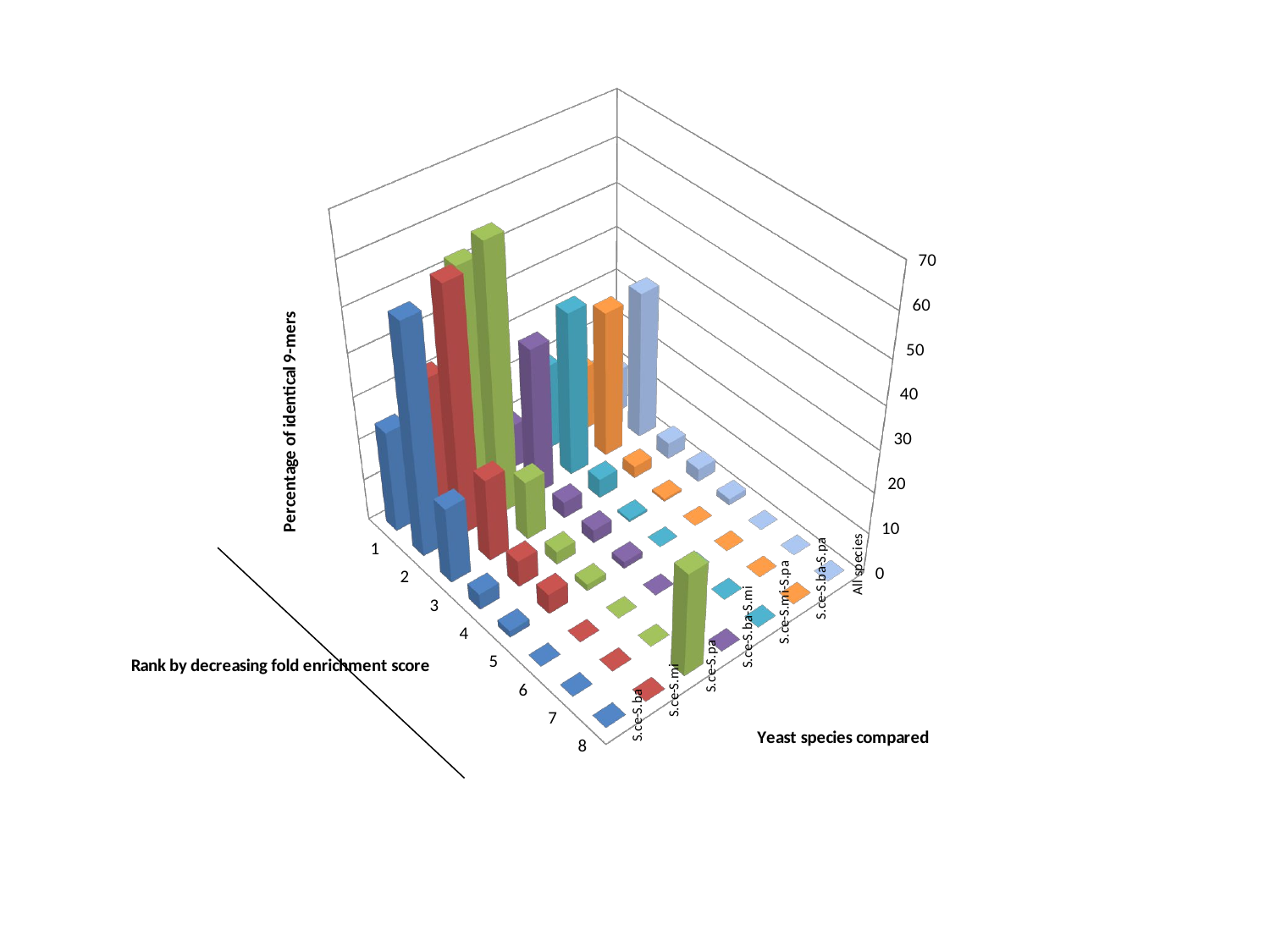

[unsupported chart]

Supplement: Figure S20 — Distributions of the percentage of identical 9-mers between each bin from S.cerevisiae and the corresponding bins from other, related yeast species. The 9-mers for each species were derived from the regions 1 kb downstream of annotated ORFs and the fold enrichment scores were calculated based on the C0/C1 method. (PPTX) [file pone.0058038.s020.pptx]

## Slide 1
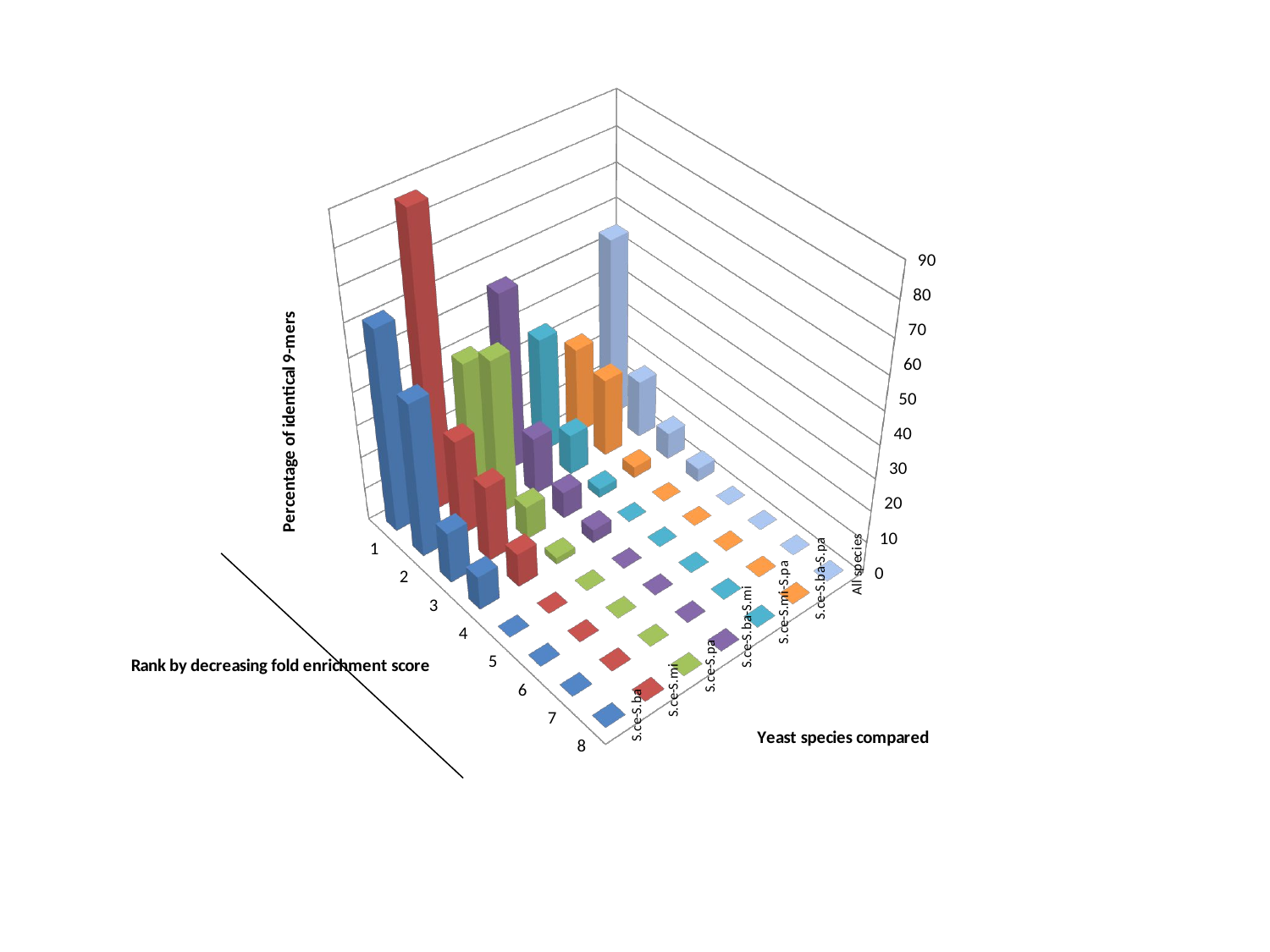

[unsupported chart]

Supplement: Figure S21 — Distributions of the percentage of identical 9-mers between each bin from S.cerevisiae and the corresponding bins from other, related yeast species. The 9-mers for each species were derived from the unannotated intergenic regions and the fold enrichment scores were calculated based on the C0/C1 method. (PPTX) [file pone.0058038.s021.pptx]
