# Supplementary material for: Comparative Analysis of DNA Word Abundances in Four Yeast Genomes Using a Novel Statistical Background Model
Source: PLoS One. 2013 Mar 5;8(3):e58038. doi: 10.1371/journal.pone.0058038 (PMC3589456; doi:10.1371/journal.pone.0058038)
Supplement: Table S1 — Comparison of the top 500 8-mers calculated using six different methods. 8-mers derived from the region 500 bases upstream of all yeast ORFs were ranked by their relative abundance as calculated by several background models. The top 20 8-mers ranked by decreasing fold enrichment scores from (a) our novel background model, the “Ak-1 method” were compared to those from the previously described C0/C1 method (b), from Markov models of order 4 sorted by Z-scores (c), from Markov models of order 4 sorted by Binomial probabilities (d), from Markov models of order 3 ranked by Z-scores (e) and, Markov models of order 3 ranked by binomial probabilities (f). The list of 500 8-mers derived by applying our method was chosen as the reference against which the other lists were compared. 8-mers in each list that are identical to the ones in the reference list are marked with a superscript number, which shows its rank in the reference column (a). The average GC content of each list is shown in the bottom row of the table, followed by the percentage of k-mers each method shares with the Ak-1 method. (DOCX) [file pone.0058038.s022.docx]

**Additional file S1**

| **No** | **Ak-1 method (a)** | **C0/C1 method**  **(b)** |  | **MM3_binomial**  **(c)** |  | **MM3_zscore**  **(d)** |  | **MM4_binomial**  **(e)** |  | **MM4_zscore**  **(f)** |  |
| --- | --- | --- | --- | --- | --- | --- | --- | --- | --- | --- | --- |
| 1 | CCTCGAGG | CCTCGAGG | ^1^ | TATATATA | ^250^ | TATATATA | ^250^ | TAATATTA |  | GCGATGAG | ^2^ |
| 2 | GCGATGAG | GCGATGAG | ^2^ | ATATATAT | ^376^ | GCGATGAG | ^2^ | GCGATGAG | ^2^ | TAATATTA |  |
| 3 | CCCAGCGC | TAGCCGCC | ^14^ | GCGATGAG | ^2^ | ATATATAT | ^376^ | AAAAGAAA | ^249^ | CCTCGAGG | ^1^ |
| 4 | CCGAGTGG | CTCGAGGA | ^8^ | TTTTTTTC | ^30^ | CCTCGAGG | ^1^ | CGGTGTTA | ^25^ | CGGTGTTA | ^25^ |
| 5 | GGAAGCTG | CGGTGTTA | ^25^ | GAAAAAAA | ^36^ | GTTACCCG | ^94^ | CCTCGAGG | ^1^ | GTTACCCG | ^94^ |
| 6 | TCCTCGAG | TCCTCGAG | ^6^ | AAAAGAAA | ^249^ | CTCGAGGA | ^8^ | GGAAGCTG | ^5^ | GGAAGCTG | ^5^ |
| 7 | CGCGTCGC | TACGGTGT | ^82^ | CCTCGAGG | ^1^ | CCGGGTAA | ^99^ | GTTACCCG | ^94^ | CTCGAGGA | ^8^ |
| 8 | CTCGAGGA | GGCGGCTA | ^23^ | CTCGAGGA | ^8^ | CTCATCGC | ^22^ | CTCGAGGA | ^8^ | CGGGTAAC | ^98^ |
| 9 | GATGAGCT | CCGGGTAA | ^99^ | GTTACCCG | ^94^ | CGGGTAAC | ^98^ | CTAGTATA | ^177^ | CCGGGTAA | ^99^ |
| 10 | GATGACGC | GGAAGCTG | ^5^ | CTCATCGC | ^22^ | TCCTCGAG | ^6^ | TTTCTTTT | ^323^ | CTAGTATA | ^177^ |
| 11 | ATACGGTG | GTTACCCG | ^94^ | TTTCTTTT | ^323^ | TTACCCGG | ^111^ | ACTTCTAG | ^122^ | ACTTCTAG | ^122^ |
| 12 | GCGCGCGC | ATACGGTG | ^11^ | CATATATA |  | CGATGAGC | ^102^ | CTCATCGC | ^22^ | CTCATCGC | ^22^ |
| 13 | GCGCCCGC | TTACCCGG | ^111^ | CCGGGTAA | ^99^ | TTTTTTTC | ^30^ | CCGGGTAA | ^99^ | TCCTCGAG | ^6^ |
| 14 | TAGCCGCC | TTTTTTTC | ^30^ | ATATGTAT |  | GAAAAAAA | ^36^ | CGGGTAAC | ^98^ | GATTCCTA | ^209^ |
| 15 | GCGACGCG | TCCGGGTA | ^452^ | ATACATAT |  | GATGAGCT | ^9^ | TGATAATG | ^363^ | AAAAGAAA | ^249^ |
| 16 | CACGTGAC | CGGGTAAC | ^98^ | TCCTCGAG | ^6^ | GGAAGCTG | ^5^ | GAAGCTGA | ^76^ | GAAGCTGA | ^76^ |
| 17 | GGATTCCT | CGATGAGC | ^102^ | TATACATA |  | ATATGTAT |  | TCCTCGAG | ^6^ | GGATTCCT | ^17^ |
| 18 | GCCCCCGG | GAAAAAAA | ^36^ | CGGGTAAC | ^98^ | ATTACCCG | ^449^ | AGGAGAAC | ^101^ | TACGGTGT | ^82^ |
| 19 | GGCGCGTC | CTCATCGC | ^22^ | TTACCCGG | ^111^ | CGGTGTTA | ^25^ | GATTCCTA | ^209^ | TTACCCGG | ^111^ |
| 20 | ACGCAAGG | ATTACCCG | ^449^ | TATGTATA |  | AAAAGAAA | ^249^ | GGATTCCT | ^17^ | AGGAGAAC | ^101^ |
| 21 | CTCGGCGG | GGATTCCT | ^17^ | TTTTCTTT |  | ATACATAT |  | TACGGTGT | ^82^ | TGATAATG | ^363^ |
| 22 | CTCATCGC | ACGCAAGG | ^20^ | GATGAGCT | ^9^ | TATACATA |  | TTACCCGG | ^111^ | ATTACCCG | ^449^ |
| 23 | GGCGGCTA | TCGAGGAG | ^47^ | GGAAGCTG | ^5^ | CATATATA |  | GCAAGGAT | ^67^ | TAGCCGCC | ^14^ |
| 24 | CGCGCGCC | GCAAGGAT | ^67^ | CGATGAGC | ^102^ | TCGAGGAG | ^47^ | GGATTGAT | ^95^ | GGATTGAT | ^95^ |
| 25 | CGGTGTTA | TATATATA | ^250^ | TAATAATA |  | TATGTATA |  | ATTACCCG | ^449^ | GCAAGGAT | ^67^ |
| 26 | CGCGACGC | ATATATAT | ^376^ | CTAGTATA | ^177^ | CGGGTAAT | ^214^ | GGAGAACT | ^59^ | TCGAGGAG | ^47^ |
| 27 | GTCACGTG | GATGAGCT | ^9^ | TACATATA |  | TAGCCGCC | ^14^ | AAACAAAA |  | TTTCTTTT | ^323^ |
| 28 | CTGCGCCG | CACGTGAC | ^16^ | ATTACCCG | ^449^ | TTTCTTTT | ^323^ | TCGAGGAG | ^47^ | ACGCAAGG | ^20^ |
| 29 | GCGTCGCG | CGGGTAAT | ^214^ | CGGTGTTA | ^25^ | CTAGTATA | ^177^ | TTTTCTTT |  | GGAGAACT | ^59^ |
| 30 | TTTTTTTC | GTCACGTG | ^27^ | TAATATTA |  | TCCGGGTA | ^452^ | GTTGGAAT |  | CGAGGAGA | ^57^ |
| 31 | GCGATGGC | CTAGTATA | ^177^ | CTTTTTTT | ^110^ | TACGGTGT | ^82^ | GATGAGCT | ^9^ | CGATGAGC | ^102^ |
| 32 | GTAATAGG | TACCCGGA | ^481^ | AAAGAAAA |  | GGATTCCT | ^17^ | TAGCCGCC | ^14^ | CGGGTAAT | ^214^ |
| 33 | CGCCCCCC | GATTCCTA | ^209^ | TGATAATG | ^363^ | TGATAATG | ^363^ | TACCTAAT | ^211^ | GATGAGCT | ^9^ |
| 34 | CACCTCCG | CTTTTTTT | ^110^ | TATTATCA |  | TACATATA |  | ACTAGTAT |  | GTTGGAAT |  |
| 35 | CACTCGGC | GGAGAACT | ^59^ | AAACAAAA |  | GAAGCTGA | ^76^ | ACGCAAGG | ^20^ | TACCTAAT | ^211^ |
| 36 | GAAAAAAA | ATCCTCGA | ^227^ | TATATATG |  | CGAGGAGA | ^57^ | CGGGTAAT | ^214^ | ATACGGTG | ^11^ |
| 37 | TCGGCGGC | CGAGGAGA | ^57^ | TCGAGGAG | ^47^ | ATCCTCGA | ^227^ | CGAGGAGA | ^57^ | ATCCTCGA | ^227^ |
| 38 | GGTACCGG | GGATTGAT | ^95^ | CGGGTAAT | ^214^ | AGGAGAAC | ^101^ | CGATGAGC | ^102^ | ACTAGTAT |  |
| 39 | GGTGTTAG | TGAAACGC | ^116^ | GGATTCCT | ^17^ | GGATTGAT | ^95^ | CTAATATT |  | TCCGGGTA | ^452^ |
| 40 | CCGACTAC | CGCAAGGA | ^105^ | ACATATAT |  | GATTCCTA | ^209^ | ATACGGTG | ^11^ | ACGGTGTT | ^163^ |
| 41 | GACGCGCC | ACGGTGTT | ^163^ | TGTATATA |  | ATACGGTG | ^11^ | CTTCTAGT | ^70^ | ATACCTAA | ^330^ |
| 42 | GGGGGGAG | ACTTCTAG | ^122^ | GAAGCTGA | ^76^ | TTTTCTTT |  | ATCCTCGA | ^227^ | TAGGATCA |  |
| 43 | AGCTCATC | TCACGTGA |  | GATGATGA | ^199^ | TAATAATA |  | ATACCTAA | ^330^ | AGGATTGA | ^212^ |
| 44 | ACCCGACC | GAAGCTGA | ^76^ | TACGGTGT | ^82^ | TAATATTA |  | AGGATTGA | ^212^ | CTTCTAGT | ^70^ |
| 45 | GGGGTTCG | AGGAGAAC | ^101^ | AGGAGAAC | ^101^ | TATTATCA |  | AAGCTGAA | ^156^ | GTAATAGG | ^32^ |
| 46 | CTCGGATC | ATTTTTTT |  | GGATTGAT | ^95^ | GATGATGA | ^199^ | GATAATGT |  | CTGAAACG | ^53^ |
| 47 | TCGAGGAG | TCGGCGGC | ^37^ | ATATATGT |  | ACTTCTAG | ^122^ | GTAATAGG | ^32^ | ATGAGATG | ^113^ |
| 48 | CGCGCGCG | CTGAAACG | ^53^ | ACTTCTAG | ^122^ | TATATATG |  | ACGGTGTT | ^163^ | GATAATGT |  |
| 49 | CCCGTCGC | AAAAGAAA | ^249^ | GATTCCTA | ^209^ | GATGAGAT | ^280^ | AAAGAAAA |  | AAGCTGAA | ^156^ |
| 50 | GCAGCAGC | AAAAAAAG | ^160^ | TAGCCGCC | ^14^ | ACACACAC | ^201^ | TATTTATA |  | AAACAAAA |  |
| 51 | CCCCCGGG | AAAAAAAT |  | ATCCTCGA | ^227^ | CACACACA | ^93^ | TATTATCA |  | TCACGTGA |  |
| 52 | CCGCTCGC | TTTCTTTT | ^323^ | TATTTATT |  | TACCTAAT | ^211^ | CTGAAACG | ^53^ | CTAATATT |  |
| 53 | CTGAAACG | GATGACGC | ^10^ | ATACGGTG | ^11^ | ACGCAAGG | ^20^ | ATGAGATG | ^113^ | AACGCAAG | ^56^ |
| 54 | CCCGGCTG | AGGATTGA | ^212^ | ATATATAA |  | AAACAAAA |  | TAGGATCA |  | TTATAGCC | ^200^ |
| 55 | GGGGCGGG | GTAATAGG | ^32^ | GATGAGAT | ^280^ | ACATATAT |  | GAGGAGAA | ^64^ | GTCACGTG | ^27^ |
| 56 | AACGCAAG | CACGTGAT | ^290^ | TCCGGGTA | ^452^ | TGTATATA |  | TCCGGGTA | ^452^ | TTTTCTTT |  |
| 57 | CGAGGAGA | GTGGAAGC | ^78^ | CGAGGAGA | ^57^ | GGAGAACT | ^59^ | AATATTAT |  | GAGGAGAA | ^64^ |
| 58 | GGGGGTGG | AAAATTTT |  | GTATATAT |  | GCAAGGAT | ^67^ | TGAAAAAT |  | CGCAAGGA | ^105^ |
| 59 | GGAGAACT | AGCCGCCC | ^288^ | TTATTTAT |  | AAAGAAAA |  | GATGAGAT | ^280^ | GATGAGAT | ^280^ |
| 60 | CCGGGTTA | GAGAACTT | ^121^ | TACCTAAT | ^211^ | CTTTTTTT | ^110^ | GAGAACTT | ^121^ | CACGTGAC | ^16^ |
| 61 | GCCGCCCA | GCTCATCG | ^479^ | TATATGTA |  | GTCACGTG | ^27^ | AACGCAAG | ^56^ | TGAAACGC | ^116^ |
| 62 | CCCCCCGC | TTTTCTTT |  | CATACATA |  | ATCTCATC | ^170^ | TAATAATA |  | GAGAACTT | ^121^ |
| 63 | TTAGCCGC | TTCTTTTT | ^414^ | ATCTCATC | ^170^ | CATACATA |  | TTATAGCC | ^200^ | TATTATCA |  |
| 64 | GAGGAGAA | GTTGGAAT |  | TATATACA |  | ATATATGT |  | TCACGTGA |  | AATAGGAT |  |
| 65 | GCGTCCCC | AGAACTTC | ^128^ | GCAAGGAT | ^67^ | TACCCGGA | ^481^ | TGAAACGC | ^116^ | TATACCTA |  |
| 66 | GCGGGCTG | TGAAAAAA |  | CACACACA | ^93^ | GCTCATCG | ^479^ | AATAGGAT |  | TAGAATTG |  |
| 67 | GCAAGGAT | TATACGGT | ^337^ | ATATATAC |  | CATATACG | ^398^ | TAGAATTG |  | GGCGGCTA | ^23^ |
| 68 | GCCGCACC | TTTTTCTT |  | GGAGAACT | ^59^ | CACGTGAC | ^16^ | ATATGTAT |  | TATTTATA |  |
| 69 | GCGACGGG | TGAAAAAT |  | ATATACAT |  | TATATGTA |  | ATCTCATC | ^170^ | ATAGGATC | ^273^ |
| 70 | CTTCTAGT | AAAAAGAA | ^389^ | ACACACAC | ^201^ | CTCATCTC | ^120^ | TATACCTA |  | GGTGTTAG | ^39^ |
| 71 | GACCTAGG | CTTCTAGT | ^70^ | AAAAAAAG | ^160^ | TATTTATT |  | CGCAAGGA | ^105^ | GTGGAAGC | ^78^ |
| 72 | CCGCGCGG | AACGCAAG | ^56^ | TTCTTTTT | ^414^ | GTATATAT |  | TCTAGTAT |  | ATCTCATC | ^170^ |
| 73 | GATCTCGC | CCCAGCGC | ^3^ | AAGCTGAA | ^156^ | AGGATTGA | ^212^ | CATATACG | ^398^ | AATATTAT |  |
| 74 | GCGGCTAA | ATATACGG | ^112^ | CATATACG | ^398^ | GTAATAGG | ^32^ | AAATTTCA |  | TCTAGTAT |  |
| 75 | GCCGCCGA | GGTGTTAG | ^39^ | ACGCAAGG | ^20^ | AAGCTGAA | ^156^ | GATTGATA |  | AAAGAAAA |  |
| 76 | GAAGCTGA | AAGGATTG | ^85^ | TATATAAA |  | CTGAAACG | ^53^ | TATACATA |  | CATATACG | ^398^ |
| 77 | GGCGTGTG | AAGAAAAA |  | ATGTATAT |  | TTATTTAT |  | GTGGAAGC | ^78^ | GTATACCT | ^264^ |
| 78 | GTGGAAGC | CAAGGATT | ^245^ | AAAAAGAA | ^389^ | AACGCAAG | ^56^ | ATCAACTA |  | TGAAAAAT |  |
| 79 | CACTCCGG | TAATATTA |  | GAGGAGAA | ^64^ | GAGATGAG | ^81^ | ATATTATA |  | GATCAATG | ^210^ |
| 80 | CCCCCCAC | GGGCGGCT |  | AGGATTGA | ^212^ | ATATATAA |  | CCTAATAT | ^188^ | TAATAATA |  |
| 81 | GAGATGAG | TTTTTTCT |  | CTCATCTC | ^120^ | TCACGTGA |  | ATAGGATC | ^273^ | ATATGTAT |  |
| 82 | TACGGTGT | AAAAATTT |  | TAGTATAT |  | TATATACA |  | GTCACGTG | ^27^ | GATTGATA |  |
| 83 | GTGACCCG | CGGCGGCT |  | TACATACA |  | GAGGAGAA | ^64^ | AACTTCTA |  | GAGATGAG | ^81^ |
| 84 | CGTTACCC | TATTATCA |  | GTAATAGG | ^32^ | ATATACAT |  | CACGTGAC | ^16^ | CCTAATAT | ^188^ |
| 85 | AAGGATTG | AAAGAAAA |  | TTCCCTTT | ^243^ | TACATACA |  | GTATACCT | ^264^ | CACGTGAT | ^290^ |
| 86 | TAACCCGG | GAAAAATT |  | AACGCAAG | ^56^ | ATACCTAA | ^330^ | GATCAATG | ^210^ | ATCAACTA |  |
| 87 | CACCCACA | CATATACG | ^398^ | ACTAGTAT |  | GGCGGCTA | ^23^ | TTGGAATA | ^425^ | AACTTCTA |  |
| 88 | CCGGGTGA | AAGCTGAA | ^156^ | CTGAAACG | ^53^ | ATATATAC |  | AAAATTTT |  | TACCCGGA | ^481^ |
| 89 | GCAGCGGC | CTCATCTC | ^120^ | ATACCTAA | ^330^ | ACGGTGTT | ^163^ | GAGATGAG | ^81^ | TATAGCCT |  |
| 90 | CGGCACCC | GCCGCCGA | ^75^ | GAGATGAG | ^81^ | TCGGGTAA |  | GGTGTTAG | ^39^ | TATACATA |  |
| 91 | CCGCCCGG | AGCTCATC | ^43^ | AAGAAAAA |  | ACTAGTAT |  | TAATGTAA |  | CTCATCTC | ^120^ |
| 92 | TGGGCGGC | ATCTCATC | ^170^ | GCTCATCG | ^479^ | ATGTATAT |  | GGCGGCTA | ^23^ | AAATTTCA |  |
| 93 | CACACACA | TGATAATG | ^363^ | GTTGGAAT |  | GTTGGAAT |  | TATAAATA |  | ATAGCCTT | ^208^ |
| 94 | GTTACCCG | GATGATGA | ^199^ | TGAAAAAT |  | AGCTCATC | ^43^ | AATGTAAT |  | TTGGAATA | ^425^ |
| 95 | GGATTGAT | AGAAAAAA |  | TACCCGGA | ^481^ | TGCGATGA |  | ATACATAT |  | TAATGTAA |  |
| 96 | CGGCCTCC | ATTTTTCA |  | TATTTATA |  | TAGTATAT |  | ATAGCCTT | ^208^ | CGCAAATG | ^151^ |
| 97 | GAGCCCCC | TCTTTTTT |  | GTCACGTG | ^27^ | CTTCTAGT | ^70^ | CTCATCTC | ^120^ | CGTTACCC | ^84^ |
| 98 | CGGGTAAC | AGCCTTTA |  | ATAAATAA |  | CGTTACCC | ^84^ | TATAGCCT |  | ATATTATA |  |
| 99 | CCGGGTAA | TTTTTTTT | ^186^ | ACGGTGTT | ^163^ | TTACCCGC | ^385^ | AGAACTTC | ^128^ | AGTGGAAG | ^152^ |
| 100 | GCAGATTC | TTATAGCC | ^200^ | CTTCTAGT | ^70^ | TGAAACGC | ^116^ | CACGTGAT | ^290^ | ACCTAATA | ^373^ |
| 101 | AGGAGAAC | GAGATGAG | ^81^ | GATAATGT |  | TAGGATCA |  | AGTGGAAG | ^152^ | CAAGGATT | ^245^ |
| 102 | CGATGAGC | CGCGCGCC | ^24^ | TCACGTGA |  | TTCCCTTT | ^243^ | TAGTATAT |  | AATGTAAT |  |
| 103 | CGGCGCGG | TAGGATCA |  | TTTTTCTT |  | CGATGAGA |  | ACCTAATA | ^373^ | AGAACTTC | ^128^ |
| 104 | GCCGGACC | TATTACCC | ^387^ | AGCTCATC | ^43^ | GATAATGT |  | CAAGGATT | ^245^ | CATCTCAT | ^181^ |
| 105 | CGCAAGGA | ATGAGATG | ^113^ | ACATATAA |  | TATATAAA |  | CATCTCAT | ^181^ | TATACGGT | ^337^ |
| 106 | GCCGGGCC | TGTTGGAA | ^286^ | ACCTAATA | ^373^ | ACCTAATA | ^373^ | AAACGCAA | ^444^ | GCTCATCG | ^479^ |
| 107 | GCGGTACC | TGCGATGA |  | CACGTGAC | ^16^ | TTCTTTTT | ^414^ | GAAAAATT |  | CTAACTAG | ^139^ |
| 108 | GCCGCGCC | CATATATA |  | TATTATTA |  | AAAAAAAG | ^160^ | TATATATA | ^250^ | AAACGCAA | ^444^ |
| 109 | GGCGCGCG | GAGGAGAA | ^64^ | TGAAACGC | ^116^ | AAAAAGAA | ^389^ | TTGATAAT |  | TCGGGTAA |  |
| 110 | CTTTTTTT | TTTTTTCA |  | AACAAACA |  | CACGTGAT | ^290^ | TATGTATA |  | TGTTACCC | ^267^ |
| 111 | TTACCCGG | CTGTATAC | ^312^ | TTTATTTA |  | TGTTACCC | ^267^ | AAGGATTG | ^85^ | CTGTATAC | ^312^ |
| 112 | ATATACGG | TTACCCGC | ^385^ | TGCGATGA |  | TTACCCGA |  | CTATTGTT |  | AAGGATTG | ^85^ |
| 113 | ATGAGATG | ATATATAA |  | TTATTATT |  | ATGAGATG | ^113^ | TTCTAGTA | ^231^ | ATACATAT |  |
| 114 | CCCCCCCC | TGACGCAA | ^285^ | ATTATCAT |  | ATGATGAG |  | TACCCGGA | ^481^ | AGCCTTTA |  |
| 115 | CAGGCCGG | CATCTCAT | ^181^ | TCGGGTAA |  | ATATACGG | ^112^ | TACTAGTA |  | AGCTCATC | ^43^ |
| 116 | TGAAACGC | CCTAATAT | ^188^ | CTATTGTT |  | GATTGATA |  | CGCAAATG | ^151^ | TACTAGTA |  |
| 117 | ACCCACAC | ATCACGTG |  | TTTCCTTT |  | CGCAAGGA | ^105^ | AGCCTTTA |  | TATAAATA |  |
| 118 | GTGGGGGG | TTGAAAAA |  | CGATGAGA |  | GAGAACTT | ^121^ | TATACGGT | ^337^ | AAAATTTT |  |
| 119 | GGCTAAGC | TGTTACCC | ^267^ | TAGGATCA |  | TATTTATA |  | TGTTGGAA | ^286^ | TTCTAGTA | ^231^ |
| 120 | CTCATCTC | ATGACGCA | ^328^ | TTGATAAT |  | ATAAATAA |  | CTGTATAC | ^312^ | GAAACGCA | ^384^ |
| 121 | GAGAACTT | AGTGGAAG | ^152^ | CTTTTCTT |  | GATCAATG | ^210^ | AACAAAAC |  | CTATTGTT |  |
| 122 | ACTTCTAG | ATAGCCTT | ^208^ | GATTGATA |  | TGAAAAAT |  | TATTATAG |  | TAGTATAT |  |
| 123 | CCACGACG | CTCGGCGG | ^21^ | ATGATGAG |  | AACAAACA |  | ATTATCAT |  | TGTTGGAA | ^286^ |
| 124 | TACCCTAC | GGATCAAT | ^415^ | GAGAACTT | ^121^ | CTATTGTT |  | AGCTGAAA |  | GATTCCAT | ^491^ |
| 125 | GCTAAGCG | GATCAATG | ^210^ | TAAATAAA |  | AATAGGAT |  | AGCTCATC | ^43^ | TTACCCGA |  |
| 126 | GGGGGCTC | GAACTTCT |  | GGCGGCTA | ^23^ | ACATATAA |  | GAAACGCA | ^384^ | GGATCAAT | ^415^ |
| 127 | CCACCCCC | GCGCGCGC | ^12^ | ATGAGATG | ^113^ | AAGAAAAA |  | TTTTGTTT |  | TTGATAAT |  |
| 128 | AGAACTTC | GCCGCCCA | ^61^ | TAAACAAA |  | GAAACGCA | ^384^ | TGTTACCC | ^267^ | TATGTATA |  |
| 129 | GCGACACC | CGTTACCC | ^84^ | ATATACGG | ^112^ | ATCAACTA |  | CGTTACCC | ^84^ | AGCTGAAA |  |
| 130 | GCGGCACT | TAGTATAT |  | AACAACAA | ^408^ | GTGGAAGC | ^78^ | GCTCATCG | ^479^ | AGGATCAA | ^233^ |
| 131 | CCTAGGTC | AAATTTTT |  | AATAGGAT |  | ATTATCAT |  | AAAACAAA |  | TATTATAG |  |
| 132 | CGTGCGCG | ATCCGGGT |  | ATCAACTA |  | TATTATTA |  | CTTTTCTT |  | GTGTTAGA | ^348^ |
| 133 | GCGCCGGG | TATATAAA |  | TTACCCGC | ^385^ | AAACGCAA | ^444^ | TTTTATTT |  | AACAAAAC |  |
| 134 | GACGCAAA | GATTGATA |  | TATAAATA |  | TCTAGTAT |  | TCGGGTAA |  | ACGGAATG | ^137^ |
| 135 | AAGGGCCC | ACTAGTAT |  | AAACGCAA | ^444^ | TTGATAAT |  | GATTCCAT | ^491^ | GAAAAATT |  |
| 136 | GGGTCCCC | TTTTTCAA |  | TGTTACCC | ^267^ | TTTATTTA |  | CTAACTAG | ^139^ | ATTATCAT |  |
| 137 | ACGGAATG | AAAAAAAA | ^184^ | GATCAATG | ^210^ | GATGACGC | ^10^ | AGGATCAA | ^233^ | GCTGAAAC | ^370^ |
| 138 | GCACGGGC | GCTGAAAC | ^370^ | TCTAGTAT |  | TTATTATT |  | GGATCAAT | ^415^ | ATTATAGC |  |
| 139 | CTAACTAG | TACCTAAT | ^211^ | CGTTACCC | ^84^ | TATACGGT | ^337^ | TATTCTGT |  | GCAGATTC | ^100^ |
| 140 | GCCGCTGC | ATGATGAG |  | TTTATTTT |  | TTTTTCTT |  | TTTTTTTC | ^30^ | TGGATTCC | ^500^ |
| 141 | CACCCCGC | ATAGGATC | ^273^ | AATAATAA |  | TCGGCGGC | ^37^ | ATTATAGC |  | TATTCTGT |  |
| 142 | GTCGGGTA | AATTTTTC |  | GAAACGCA | ^384^ | AACAACAA | ^408^ | TTCCCTTT | ^243^ | TCCGCGGA |  |
| 143 | GTTAGAGG | GCGATGGC | ^31^ | AAGAACAA |  | TAAACAAA |  | GCTGAAAC | ^370^ | TATATATA | ^250^ |
| 144 | CCTCGACT | AAAAAAGA |  | CACGTGAT | ^290^ | TATACCTA |  | GATGATGA | ^199^ | TCGGCGGC | ^37^ |
| 145 | CGCACGGC | AAAAAATT |  | CGCAAGGA | ^105^ | ATAGGATC | ^273^ | TTACCCGA |  | ACCCATAC | ^314^ |
| 146 | CCCACACA | TAATAATA |  | ATAATAAT |  | TATGTATG |  | ATTCTGTA |  | GATGACGC | ^10^ |
| 147 | CGCCCGGC | TTGGAATA | ^425^ | TTACCCGA |  | GCAGATTC | ^100^ | ATATTATC |  | CACCCACA | ^87^ |
| 148 | CCACACCC | TCGGGTAA |  | AAGAAAAG |  | TTTCCTTT |  | GTGTTAGA | ^348^ | ATTCTGTA |  |
| 149 | GCGCGGTG | TATAGCCT |  | GTGGAAGC | ^78^ | TAAATAAA |  | ATATACGG | ^112^ | ATATACGG | ^112^ |
| 150 | GCCCGGGC | GGGGCGGG | ^55^ | TACTAGTA |  | ACACATAC |  | TAATAGGA |  | TTTTGTTT |  |
| 151 | CGCAAATG | GATGAGAT | ^280^ | AAAACAAA |  | TACTAGTA |  | ACAAAACA |  | TAATAGGA |  |
| 152 | AGTGGAAG | TGGAAGCT |  | TATACCTA |  | CTTTTCTT |  | CCATTTTG |  | GATGATGA | ^199^ |
| 153 | GGCGGACC | GCACGTGA |  | AATGATGA |  | TGTGTGTG | ^252^ | GCAGATTC | ^100^ | CCATTTTG |  |
| 154 | TGGTCGGG | GGTGGCAA | ^183^ | TATACGGT | ^337^ | AGCCTTTA |  | ATAATGTA |  | AAAACAAA |  |
| 155 | CGGGTCAC | TCCGCGGA |  | AGCCTTTA |  | ATGCGATG | ^311^ | GAAAAAAA | ^36^ | CTTTTCTT |  |
| 156 | AAGCTGAA | CTAACTAG | ^139^ | TATGTATG |  | AGTATGTA |  | GAACTTCT |  | GTGGCAAA | ^448^ |
| 157 | GCCGAGTG | GAAACGCA | ^384^ | CATCTCAT | ^181^ | AGTGGAAG | ^152^ | ACGGAATG | ^137^ | TTCCCTTT | ^243^ |
| 158 | ACCTCGGC | AGCTGAAA |  | AGTGGAAG | ^152^ | TATAAATA |  | ATTTTTCA |  | CAGATTCC | ^396^ |
| 159 | GCCGAGGG | TTGATAAT |  | TAATGTAA |  | CCTTTATC | ^434^ | TAGCCTTT |  | CACTATCG | ^193^ |
| 160 | AAAAAAAG | ACCTAATA | ^373^ | CCTTTATC | ^434^ | CATCTCAT | ^181^ | GTGGCAAA | ^448^ | TTACCCGC | ^385^ |
| 161 | TTCCGCGG | GGGGTTCG | ^45^ | ACACATAC |  | CGCAAATG | ^151^ | TGGATTCC | ^500^ | GACGCAAA | ^134^ |
| 162 | GGCCGGCC | CGCGCGCG | ^48^ | AGTATGTA |  | ATCCGGGT |  | CATATAAA |  | TTTTATTT |  |
| 163 | ACGGTGTT | TGGGCGGC | ^92^ | AGCTGAAA |  | AAGAACAA |  | TCAATGAA |  | GAACTTCT |  |
| 164 | CAATGCGG | CGCAAATG | ^151^ | ATAGGATC | ^273^ | GGATCAAT | ^415^ | TGAAATTT |  | ACAAAACA |  |
| 165 | ATAGCCGC | GTATACCT | ^264^ | GAAAAATT |  | AATGATGA |  | ATGAATAT |  | TAGCCTTT |  |
| 166 | CGAAGCGG | TAGCCTTT |  | AAGGATTG | ^85^ | GGTGTTAG | ^39^ | GATGACGC | ^10^ | TAATCGTA |  |
| 167 | CCGCTCCC | TTAGCCGC | ^63^ | GATGACGC | ^10^ | AATAATAA |  | CAAAACAA |  | ATATTATC |  |
| 168 | ACCCTACC | AATGATGA |  | GCAGATTC | ^100^ | AAGGATTG | ^85^ | CACCCACA | ^87^ | ATAATGTA |  |
| 169 | CACACCCA | AGTATATT |  | ATAGCCTT | ^208^ | ATAGCCTT | ^208^ | ACCCATAC | ^314^ | GTTAAGAT |  |
| 170 | ATCTCATC | AGTCATCT |  | ACATACAT |  | GCGGGTAA |  | GACGCAAA | ^134^ | TATCCTCG |  |
| 171 | GCGTAGGT | CCGAGTGG | ^4^ | CATATAAA |  | ACATACAT |  | TCATATAC |  | TCAATGAA |  |
| 172 | GCCCGTGC | TTACCCGA |  | TAGAATTG |  | ACCCACAC | ^117^ | ATAATATT |  | CTAAATCC |  |
| 173 | CATCGCGA | GGGTAACA |  | AAAATAAA |  | TAATGTAA |  | TTTATTTT |  | TTTTTTTC | ^30^ |
| 174 | GAGAGCGG | ATCAACTA |  | ATATAGAT |  | TATTACCC | ^387^ | TAATCGTA |  | TTGTGTAG | ^353^ |
| 175 | GCGATGCC | ATATAAAA |  | TTTTATTT |  | GTATGTAG |  | ATTTATAG |  | ATCACGTG |  |
| 176 | GCCCCTGC | ACGGAATG | ^137^ | TATTACCC | ^387^ | TAGAATTG |  | CCTTTATC | ^434^ | CCTAAATC |  |
| 177 | CTAGTATA | AAATTTCA |  | ATAATGTA |  | AGCTGAAA |  | TTACCCGC | ^385^ | ATGAATAT |  |
| 178 | GAGGATTC | TGTATACC |  | AATGTAAT |  | ATAATAAT |  | TATCATAT |  | TCATATAC |  |
| 179 | CGCTACCA | GCAGATTC | ^100^ | ATGATGAT |  | TTTATTTT |  | CAGATTCC | ^396^ | CCTTTATC | ^434^ |
| 180 | GCCCGACG | GTGGCAAA | ^448^ | GGATCAAT | ^415^ | CTGTATAC | ^312^ | GTTAAGAT |  | CAAAACAA |  |
| 181 | CATCTCAT | ACCACACC | ^190^ | TGTGTGTG | ^252^ | CCCACACA | ^146^ | AAGAAAAG |  | CATATAAA |  |
| 182 | CCACGGGA | TCAATGAA |  | TGTTGGAA | ^286^ | CCTAAATC |  | TTGTGTAG | ^353^ | GTTAGAGG | ^143^ |
| 183 | GGTGGCAA | TATATTAT |  | TCCCTTTT |  | GCTGAAAC | ^370^ | AAATAAAA |  | TGAAATTT |  |
| 184 | AAAAAAAA | AATAATAT |  | CTGTATAC | ^312^ | AAGAAAAG |  | TGTAATAG |  | ATTTATAG |  |
| 185 | TGCCGTCC | AACTTCTA |  | TAGCCTTT |  | AAAACAAA |  | CACTATCG | ^193^ | CGATGAGA |  |
| 186 | TTTTTTTT | ATATTATA |  | CGCAAATG | ^151^ | CTAACTAG | ^139^ | TTGTTTTG |  | ATTTTTCA |  |
| 187 | CGGACCGC | TTTTTCAC |  | ATGCGATG | ^311^ | CTAAATCC |  | TATTACCC | ^387^ | TATTACCC | ^387^ |
| 188 | CCTAATAT | ACCCTACC | ^168^ | CAAGGATT | ^245^ | TCATCTAC | ^468^ | AATTAGTG |  | AATTAGTG |  |
| 189 | CGCCTCGG | TATATATG |  | AATAAATA |  | TAGCCTTT |  | TCGGCGGC | ^37^ | TGTAATAG |  |
| 190 | ACCACACC | CACCTCCG | ^34^ | GCTGAAAC | ^370^ | CAAGGATT | ^245^ | TTTCACTT |  | GTTAGAAG | ^345^ |
| 191 | CCGGGGGC | AGGATCAA | ^233^ | TCATCTAC | ^468^ | CTACATAC |  | TATCCTCG |  | ACCCACAC | ^117^ |
| 192 | CGTAGGTC | GTCGGGTA | ^142^ | AGAACTTC | ^128^ | TGTTGGAA | ^286^ | AAATGAAA |  | TGGAAGCT |  |
| 193 | CACTATCG | AATATTAT |  | ATACATAA |  | ATGATGAT |  | CTAAATCC |  | CACACCCA | ^169^ |
| 194 | CGGTACCC | GCCGGGTA |  | TAAATAAT |  | ATATAGAT |  | TATAAAAC |  | ACCCTACC | ^168^ |
| 195 | GAATGAGG | TACCCTAC | ^124^ | GGTGTTAG | ^39^ | GCCGCCGA | ^75^ | CGATGAGA |  | TGACGCAA | ^285^ |
| 196 | GAACGCGC | AATTTTTT |  | TTCTAGTA | ^231^ | GACGCAAA | ^134^ | TCCGCGGA |  | GAAAAAAA | ^36^ |
| 197 | GGAATGAG | TCATATAC |  | CCTAATAT | ^188^ | ATAATGTA |  | GTTAGAAG | ^345^ | TATCATAT |  |
| 198 | GGATCCGC | GATAATGT |  | ATTATTTA |  | AATGTAAT |  | ATGATGAG |  | ATGGATTC |  |
| 199 | GATGATGA | CGCGACGC | ^26^ | GTATGTAG |  | TCATCGCA |  | TGGAAGCT |  | CTATATCC |  |
| 200 | TTATAGCC | AAATGATG |  | ATTATCAA |  | CAGATTCC | ^396^ | TTTCCTTT |  | TTAGCCGC | ^63^ |
| 201 | ACACACAC | GTATATAT |  | AGGATCAA | ^233^ | CACACCCA | ^169^ | AAAAAGAA | ^389^ | ATAATATT |  |
| 202 | CACCCCCC | AGATGACG |  | TAATAGGA |  | AGGATCAA | ^233^ | CCTAAATC |  | GGGTAACA |  |
| 203 | GACGACGA | GATTCCAT | ^491^ | CAAACAAA |  | CATATAAA |  | TAAATTAG |  | TACCCTAC | ^124^ |
| 204 | GCGGCCGC | GACGCAAA | ^134^ | CTACATAC |  | TGGATTCC | ^500^ | TTTCATTT |  | CCCAGCGC | ^3^ |
| 205 | GTAGGTCC | AGCCGCCG |  | GCGGGTAA |  | ATGGATTC |  | ATCACGTG |  | TGTGTAGA |  |
| 206 | GGCTGAGC | AATAGGAT |  | TCGGCGGC | ^37^ | AGAACTTC | ^128^ | CATTTTGA |  | AGCCGCCC | ^288^ |
| 207 | CCTCGGCG | CGATGAGA |  | GACGCAAA | ^134^ | CCTAATAT | ^188^ | AAAGTGAA |  | CCTATATC |  |
| 208 | ATAGCCTT | GGGTAATA |  | CCTAAATC |  | GGGTAACA |  | ATGGATTC |  | ATGATGAG |  |
| 209 | GATTCCTA | ACATATAT |  | TCATCGCA |  | GAAAAATT |  | TGACGCAA | ^285^ | GGTGGCAA | ^183^ |
| 210 | GATCAATG | GCAGCAGC | ^50^ | ACCCACAC | ^117^ | TCCCTTTT |  | CTATATCC |  | AACGGAAT |  |
| 211 | TACCTAAT | TACCCGCA |  | TTTTGTTT |  | ATCACGTG |  | AATGAAAA |  | TGTATACC |  |
| 212 | AGGATTGA | TTCTAGTA | ^231^ | AACTTCTA |  | TTCTAGTA | ^231^ | TGTGTAGA |  | TTGTTTTG |  |
| 213 | GACGAGGA | CACACCCA | ^169^ | CTAAATCC |  | TAATAGGA |  | TGTATACC |  | GCCGCCGA | ^75^ |
| 214 | CGGGTAAT | GGCGCGTC | ^19^ | AAACAAAC |  | GATTCCAT | ^491^ | GTTAGAGG | ^143^ | TAAATTAG |  |
| 215 | CCGCGGGG | CATATAAA |  | TTCCTTTT |  | ATACATAA |  | CAATGAAT |  | CGGAATGA | ^292^ |
| 216 | AGATCGGG | AATATATA |  | CCCACACA | ^146^ | TCGCTATC |  | TTCTTTTT | ^414^ | TTTATTTT |  |
| 217 | GTGCGGGT | GTGTTAGA | ^348^ | AAATTTCA |  | TCATCGCT |  | ATCAATGA |  | TTTCACTT |  |
| 218 | GCCGTGGC | CGGAATGA | ^292^ | AAAGTGAA |  | GTATACCT | ^264^ | AACGGAAT |  | TATAAAAC |  |
| 219 | GGCGCTTG | TCATCTCA |  | CTAACTAG | ^139^ | AAAATAAA |  | CCTATATC |  | GTTACCCT |  |
| 220 | CGCGCCGG | GGCGATGA |  | AAAGGAAA |  | GGCGATGA |  | GGGTAACA |  | ACACCCAC |  |
| 221 | CCGCTACC | TAATAGGA |  | TGATGAGA |  | GCACGTGA |  | GGTGGCAA | ^183^ | ATCCGGGT |  |
| 222 | CACCGCAC | TCTAGTAT |  | ATGGATTC |  | ATACGTAC |  | CACACCCA | ^169^ | GCACGTGA |  |
| 223 | GTAACTCC | AAGGGCCC | ^135^ | GATTCCAT | ^491^ | TTTTATTT |  | AAAATAAA |  | AAGAAAAG |  |
| 224 | GGAATGCC | CCCGGCTG | ^54^ | TAAATTAG |  | AGCCGCCC | ^288^ | ACCCACAC | ^117^ | ATGCGATG | ^311^ |
| 225 | GGCGCCCC | ATAATATA |  | ATTTATTT |  | GTTACCCT |  | TTTAATTT |  | CATTTTGA |  |
| 226 | CGATCCCC | GCGGGTAA |  | ATTGATAA |  | GCCGGGTA |  | GGAATAAA |  | CAATGAAT |  |
| 227 | ATCCTCGA | TTTGAAAA |  | ATCCGGGT |  | CGGCGGCT |  | ACATATAA |  | AAATGAAA |  |
| 228 | CGTCTATC | TTGTGTAG | ^353^ | GTATACCT | ^264^ | ACCCATAC | ^314^ | TACCCTAC | ^124^ | AAATAAAA |  |
| 229 | AGCGTAGG | ATGATGAT |  | ACAAACAA |  | TGATGAGA |  | TTATCATA |  | CATTACCC |  |
| 230 | CGACGTCC | TTTTCAAA |  | TCATCGCT |  | TATCCTCG |  | GTTACCCT |  | ATCAATGA |  |
| 231 | TTCTAGTA | TGATGAGA |  | GGGTAACA |  | TAAATAAT |  | TGTTTTAT |  | AAAGTGAA |  |
| 232 | CATCGCTA | ATATATAC |  | CAGATTCC | ^396^ | AACTTCTA |  | TTAGCCGC | ^63^ | TTACGTTA |  |
| 233 | AGGATCAA | TCATCGCA |  | TGGATTCC | ^500^ | ATTATCAA |  | TTACGTTA |  | GTCGGGTA | ^142^ |
| 234 | GCGCAGGC | ATACCTAA | ^330^ | ATAAACAA |  | AATAAATA |  | TGTAAATA |  | CTACCATC | ^319^ |
| 235 | TACCCGAC | ATATTATC |  | ATATTATC |  | GTCATCTA |  | AAATTAAA |  | GCGGGTAA |  |
| 236 | TCGCGGCG | CTACTAAC | ^247^ | CACACCCA | ^169^ | TAAATTAG |  | ATGCGATG | ^311^ | TTTCCTTT |  |
| 237 | CTCCCCCC | AATGTAAT |  | ATCAATGA |  | TCTCATCG |  | CGGAATGA | ^292^ | TGCGATGA |  |
| 238 | CCCCGTGG | GTTACCCT |  | TCAATGAA |  | ATTATTTA |  | ACCCTACC | ^168^ | GGGGCGGG | ^55^ |
| 239 | GCCACCGG | GTGAAAAA |  | GGAATAAA |  | CAAACAAA |  | CATTACCC |  | TTTCATTT |  |
| 240 | TCGGTAGC | GGCGCGCG | ^109^ | TTTCACTT |  | AAACAAAC |  | TGCGATGA |  | AGCGTAGG | ^229^ |
| 241 | GGGGGCCC | TCACGTGC |  | TTGGAATA | ^425^ | GTCGGGTA | ^142^ | ATTGATAA |  | GCTATTGT |  |
| 242 | TGTGCGTG | GCGGCTAA | ^74^ | GTGGCAAA | ^448^ | GTGGCAAA | ^448^ | GCTATTGT |  | ATGACGCA | ^328^ |
| 243 | TTCCCTTT | CTAATATT |  | AACATATA |  | AAAGTGAA |  | ATTCCATT |  | GGAATAAA |  |
| 244 | AGGGGAGG | TAGAATTG |  | ATCACGTG |  | TATGTGTA |  | TTTATAGA |  | AGATGACG |  |
| 245 | CAAGGATT | CATTACCC |  | AAAAAAAT |  | TGTATGTG |  | ACACCCAC |  | AATGAAAA |  |
| 246 | CGCCGGAG | ATATCCTC | ^411^ | TACATAAT |  | TTAGCCGC | ^63^ | ATTTTGAG |  | AAAAAGAA | ^389^ |
| 247 | CTACTAAC | CACTATCG | ^193^ | GTCATCTA |  | ATCAATGA |  | TTATTTAT |  | TTATCATA |  |
| 248 | GCAGCGCC | CCCACACA | ^146^ | ATACGTAC |  | ATTGATAA |  | CTACCATC | ^319^ | CTACTAAC | ^247^ |
| 249 | AAAAGAAA | GAGGATTC | ^178^ | TCTCATCG |  | TACACATA |  | TTCCATTT |  | TCGCTATC |  |
| 250 | TATATATA | TGTGTAGA |  | TACACATA |  | ACAAACAA |  | GCACGTGA |  | AGATTCCA |  |
| 251 | CTATCGTC | TTTATATA |  | GTTACCCT |  | AAATTTCA |  | AAGTGAAA |  | TCGGTAGC | ^240^ |
| 252 | TGTGTGTG | TGAGGAAT |  | AAGTGAAA |  | TGACGCAA | ^285^ | AGCCGCCC | ^288^ | TGTTTTAT |  |
| 253 | GGGCGGGG | TATATATT |  | TATGTATT |  | TATACGTA |  | ATGACGCA | ^328^ | GCAGCAGC | ^50^ |
| 254 | CGCGAACG | CGCTACCA | ^179^ | GGCGATGA |  | CACCCACA | ^87^ | AGATTCCA |  | ACATATAA |  |
| 255 | CGACTCCC | TTATTTTT |  | TATGTGTA |  | AGCGATGA |  | CTAGTATT |  | TACCCGAC | ^235^ |
| 256 | CCGTCTGG | TGTAATAG |  | TATCCTCG |  | TTTTGTTT |  | AGATGACG |  | TTTAATTT |  |
| 257 | CGTCCACC | TGAAATTT |  | TCTTTTTC |  | TCAATGAA |  | AAAGGAAA |  | TGTAAATA |  |
| 258 | GCGGGTCG | CCGACTAC | ^40^ | TCGCTATC |  | TACGTATA |  | GCGGGTAA |  | ATTCCATT |  |
| 259 | AAGGCACC | GCGCCCGC | ^13^ | TATACGTA |  | TTCCTTTT |  | ATCCGGGT |  | CTATCGTC | ^251^ |
| 260 | CCGGAGTG | AGGATTCC |  | AACAATAG |  | GACGAAGA | ^351^ | CTACTAAC | ^247^ | AATCCTCG |  |
| 261 | GCAGTAGC | CCACACCC | ^148^ | ACCCATAC | ^314^ | TACATAAT |  | AAAATGAA |  | ATTTTGAG |  |
| 262 | CGTTGGCG | TTTTGAAA |  | TACGTATA |  | AAAGGAAA |  | TTTGTTTT |  | CTAGTATT |  |
| 263 | GGGTTCGA | TATCCTCG |  | AAGGGAAA |  | ATATTATC |  | TATTATTA |  | GCCGGGTA |  |
| 264 | GTATACCT | AAATTTTC |  | TGTAAATA |  | GGAATAAA |  | TTTTCACT |  | TTCTTTTT | ^414^ |
| 265 | CGCCGACG | TTTTTTTA |  | TCATCTAA |  | TTGGAATA | ^425^ | ATATTTAT |  | AGGGTCCA |  |
| 266 | GCGGGCAA | CGTTACTA |  | ATTTTTCA |  | ATTTATTT |  | TATTTATT |  | AAAATAAA |  |
| 267 | TGTTACCC | CACCCACA | ^87^ | AAATAAAA |  | ATAAACAA |  | TCCCTTTT |  | TTTATAGA |  |
| 268 | CATCTACT | TACCCGGC |  | CAATGAAT |  | CATCGCTA | ^232^ | GCAGCAGC | ^50^ | ATTGATAA |  |
| 269 | CTGAAGTG | ATTATCAT |  | TCTACATA |  | TACACACA |  | AATAATAT |  | CCCATACA |  |
| 270 | CAACTATC | CGGCACCC | ^90^ | GACGAAGA | ^351^ | TATGTATT |  | GCCGCCGA | ^75^ | AAATTAAA |  |
| 271 | GGTTAAGG | ATCAATGA |  | TGTATGTG |  | CATTACCC |  | CCCAGCGC | ^3^ | GGGGTTCG | ^45^ |
| 272 | CGCATACG | ATAATAAT |  | TCATATAC |  | TATAGCCT |  | AGTATATT |  | ACTAACTA |  |
| 273 | ATAGGATC | TTATATAT |  | TGACGCAA | ^285^ | TTTCACTT |  | TATAGATT |  | CTCGGCGG | ^21^ |
| 274 | GTAGTCGG | TTCCCTTT | ^243^ | AGCGATGA |  | TCATCTAA |  | ACTAACTA |  | TAACTAGT |  |
| 275 | CCCCGCCG | TTTTTTTG |  | TATAGCCT |  | AGTCATCT |  | GCAAATGA |  | CGTTACTA |  |
| 276 | CTCGGCCG | GAAAATTT |  | AGTCATCT |  | TCTACATA |  | ATATTCTG |  | CGGAACCG | ^343^ |
| 277 | CGCCCCGC | TGTATATA |  | GCACGTGA |  | CGTTACTA |  | TCGCTATC |  | CCGAGTGG | ^4^ |
| 278 | GGCCGGTG | ACCCACAC | ^117^ | ATACACAT |  | GCAGCAGC | ^50^ | AAACGGAA | ^395^ | TTCCATTT |  |
| 279 | GGGTCCTG | CACACACA | ^93^ | TCATCTCA |  | AGATGACG |  | GTTTTGTT |  | ACACACAC | ^201^ |
| 280 | GATGAGAT | ACATATAA |  | TATTCTGT |  | AACAATAG |  | AATCAACT |  | ATCCGTAC |  |
| 281 | GCGGGCGC | AGGGTCCA |  | GCCGCCGA | ^75^ | GTCATCGA |  | TAACTAGT |  | GCAAATGA |  |
| 282 | GCCCGAAG | TGGATTCC | ^500^ | TTATTTTT |  | CAATGAAT |  | ATAAAACG |  | GAAGCTGT |  |
| 283 | GGAGCTGG | CGGTTCGA |  | TACACACA |  | ATGACGCA | ^328^ | AAAATTAA |  | AGGATTCC |  |
| 284 | CCGCCGGC | CTTTTCTT |  | TTATGTAT |  | CTACTAAC | ^247^ | TGGAATAA |  | AAGTGAAA |  |
| 285 | TGACGCAA | GTTAGAAG | ^345^ | TGTAATAG |  | AACATATA |  | AATCCTCG |  | CGCTACCA | ^179^ |
| 286 | TGTTGGAA | TAGTCATC |  | TGGAAGCT |  | TGGAAGCT |  | ACACACAC | ^201^ | TTATTTAT |  |
| 287 | CGACGGGG | AATAAAAA |  | CACCCACA | ^87^ | AAGTGAAA |  | AATGATGA |  | CGGCGGCT |  |
| 288 | AGCCGCCC | GGCTAAGC | ^119^ | TGTATGTA |  | TGTATGTA |  | CGTTACTA |  | AAACGGAA | ^395^ |
| 289 | CGCCCCTT | TCGCTATC |  | AAAGGGAA |  | ATACACAT |  | GAAGCTGT |  | AATCAACT |  |
| 290 | CACGTGAT | GCCTTTAT |  | AGCAAACA |  | ACGGAATG | ^137^ | ATAAAACA |  | CCCACACA | ^146^ |
| 291 | CAGGGCGG | ATATAATA |  | CATTACCC |  | TCATCTCA |  | ATGTAATA |  | ACTATCGT |  |
| 292 | CGGAATGA | TAATGTAA |  | TGATAATA |  | CACTATCG | ^193^ | CATACATA |  | TATAGATT |  |
| 293 | TGAACCGC | GCTAAGCG | ^125^ | CGTTACTA |  | TGTAAATA |  | CCCATACA |  | CCGAAATC |  |
| 294 | CCGGTACC | ATTATAGC |  | AGTGAAAA |  | ATGTATGT |  | GTCGGGTA | ^142^ | ATATTCTG |  |
| 295 | GCCTATGG | CAAAAAAA |  | AAAAGGAA |  | AAGGGAAA |  | CTATCGTC | ^251^ | AGGCTAAG |  |
| 296 | GTACCCTC | ATATATGT |  | TTAGCCGC | ^63^ | TGTGTAGA |  | TATAGAAT |  | ATAAAACG |  |
| 297 | CAGTGGCC | GTGCACCA | ^365^ | CTTTATCA |  | TGTAATAG |  | TTCATGTT |  | GCGATGGC | ^31^ |
| 298 | GGAATCCC | GTATATTC |  | AGATGACG |  | TCATATAC |  | TCAACTAT |  | TCACGTGC |  |
| 299 | TCACCCGG | AAACAAAA |  | TTCTTTTC |  | GGGTAACG |  | CCCTTTTA |  | TTTTCACT |  |
| 300 | CGGCCCGC | AATCCTCG |  | GCAGCAGC | ^50^ | TTATGTAT |  | AGCGTAGG | ^229^ | TCCCTTTT |  |
| 301 | GCTCCTGC | TCGGTAGC | ^240^ | GAACTTCT |  | TATTCTGT |  | TTCACTTT |  | GTTTTGTT |  |
| 302 | GGAGCGCT | AAGAAAAG |  | TTTATGTA |  | TAGTCATC |  | CAAATGAT |  | ACGCGAAA |  |
| 303 | CACTGCGC | TATTTTTT |  | CATCGCTA | ^232^ | AGCAAACA |  | ATGATGAT |  | AAAGGAAA |  |
| 304 | CCGCCCCC | ATAATATT |  | CTACTAAC | ^247^ | TACCCTAC | ^124^ | ATCAAAAC |  | TGGAATAA |  |
| 305 | CGGCTGAG | GAGAAGCT |  | GCCGGGTA |  | GGTGGCAA | ^183^ | AATTTCAA |  | AAAATGAA |  |
| 306 | CGGTTCCG | AGGAAGCT |  | CGGCGGCT |  | TCTTTTTC |  | ATAGTCAT |  | TTCATGTT |  |
| 307 | GGCGGAGC | TCTTTTTC |  | ATGTATGT |  | ATACATAC |  | AGGATTCC |  | AGTATATT |  |
| 308 | CACCATGG | GTACCCTC | ^296^ | AGCCGCCC | ^288^ | GCGATGGC | ^31^ | TCGGTAGC | ^240^ | TATTATTA |  |
| 309 | GAAGACGA | TCATCGCT |  | AATAAAAA |  | ATCTACTA |  | ACTATCGT |  | TATCGTCT |  |
| 310 | CCGGTTCG | ATCTGCTC |  | AACAAAAC |  | CCTCATCG |  | TATAATAT |  | CCACACCC | ^148^ |
| 311 | ATGCGATG | GTTAGAGG | ^143^ | TGTGTAGA |  | AATCCTCG |  | AGTGAAAA |  | CATACATA |  |
| 312 | CTGTATAC | CATCTACT | ^268^ | ATATTTAT |  | TATGTACG |  | AACAACAA | ^408^ | ATATTTAT |  |
| 313 | GCCCGGCG | AAAATTTC |  | ATGACGCA | ^328^ | GAACTTCT |  | CCCACACA | ^146^ | TTTGTTTT |  |
| 314 | ACCCATAC | ATGCGATG | ^311^ | ATACATAC |  | TCACGTGC |  | AGGGTCCA |  | TCAACTAT |  |
| 315 | GACATGGC | ATTGAAAA |  | GTCATCGA |  | AAAAAAAT |  | TGAGGAAT |  | TAAAACGG |  |
| 316 | CCCTAACC | ATGAAAAA |  | CTAATATT |  | CTTTATCA |  | CACACACA | ^93^ | CGGTACCC | ^194^ |
| 317 | GGCCCCCG | CTGTCATC | ^326^ | ATGTAATA |  | ATTTTTCA |  | TATCGTCT |  | GCTAAGCG | ^125^ |
| 318 | TCGACTCC | TACTAGTA |  | ATCTACTA |  | GGTTACCC |  | TAAAACGG |  | TATTTATT |  |
| 319 | CTACCATC | GCGACGCG | ^15^ | TTTTTTCT |  | TTTATGTA |  | ATATAGAT |  | AATAATAT |  |
| 320 | CGGCGCCG | GGCGCTTG | ^219^ | TAGTCATC |  | AAAGGGAA |  | ATAATAAT |  | GGCTAAGC | ^119^ |
| 321 | TCTCCCGC | CTGAAAAA |  | AAATAAAT |  | ATCATCTA |  | AATCGTAA |  | ATAGTCAT |  |
| 322 | GTCGCCGC | CCGGGTGA | ^88^ | ATCATCTA |  | AAATAAAA |  | GCCGGGTA |  | ATGTAATA |  |
| 323 | TTTCTTTT | CAATGAAT |  | ACAATAGA |  | GGGGCGGG | ^55^ | AATGAATA |  | CCCTTTTA |  |
| 324 | GGGTGACC | AGATGATG |  | ACGGAATG | ^137^ | GTGTTAGA | ^348^ | AAAAGGAA |  | AATGATGA |  |
| 325 | GGAACACC | GTATGTAG |  | TTTTCACT |  | TGATAATA |  | TCTTTTCT |  | AATCGTAA |  |
| 326 | CTGTCATC | AACATATA |  | ATATGTGT |  | TACCCGAC | ^235^ | TACCCGAC | ^235^ | TATAGAAT |  |
| 327 | CCGTCCGC | GAAGAAAA |  | GTCGGGTA | ^142^ | ATCCGTAC |  | ACGCGAAA |  | GGGTAACG |  |
| 328 | ATGACGCA | ATTATCAA |  | GGTGGCAA | ^183^ | AGCCGCCG |  | TACATATA |  | CACACACA | ^93^ |
| 329 | GGGCGGAC | CAAATGAT |  | AATTAGTG |  | TTATAGCC | ^200^ | AAATCAAC |  | CAAATGAT |  |
| 330 | ATACCTAA | AGGGGCGG |  | ATATAAAT |  | GACGACGA | ^203^ | ATCCGTAC |  | ATCAAAAC |  |
| 331 | CACATCGC | CCTTTATC | ^434^ | TTCCATTT |  | ATATGTGT |  | TCATCTCA |  | TGAGGAAT |  |
| 332 | GCTGCTGC | TACAGCGG |  | CACTATCG | ^193^ | AATTAGTG |  | ATATCCTC | ^411^ | ATAAAACA |  |
| 333 | CCGTCTAG | ATACATAT |  | TTATAGCC | ^200^ | ACCCTACC | ^168^ | GCCTTTAT |  | AAAATTAA |  |
| 334 | GCCGTCCA | GCGTCGCG | ^29^ | AATCAACT |  | CCCATACA |  | CGCTACCA | ^179^ | GTATGGGT |  |
| 335 | CGACGAGG | TTGCGATG |  | GCAAATGA |  | AGTGAAAA |  | TAGGATAA |  | TCGTCAGA |  |
| 336 | ACGGGTCC | TTATATAA |  | AGATGATG |  | GTGTGTGT |  | TCATCTAC | ^468^ | TAGGATAA |  |
| 337 | TATACGGT | TTTTTTCC |  | TTATCAAC |  | CCCAGCGC | ^3^ | ATTCCTAA |  | GCGGCTAA | ^74^ |
| 338 | GACCGGGG | TCATCATC |  | ATCTAAAT |  | ATGTAATA |  | ACAATAGA |  | CACCTCCG | ^34^ |
| 339 | GGAGCGAC | CTGAAGTG | ^269^ | AACAATAA |  | ACAATAGA |  | CCGAAATC |  | ATGATGAT |  |
| 340 | GCCGGGCG | GCCCCCGG | ^18^ | TATATAAG |  | AACAAAAC |  | AATATTAA |  | GGTTACCC |  |
| 341 | GCGGCCGG | AATTAGTG |  | ATGATGAC |  | GCAAATGA |  | GGGGCGGG | ^55^ | TTCACTTT |  |
| 342 | TCGACAGG | AAACGCAA | ^444^ | ATAAATAG |  | ATGATGAC |  | AATTGCAG |  | ATATCCTC | ^411^ |
| 343 | CGGAACCG | GGCGTGTG | ^77^ | AATCCTCG |  | CGTCTATC | ^228^ | AAATGATG |  | ACCACACC | ^190^ |
| 344 | CCACGGCC | TAAAAAAA |  | TACCCTAC | ^124^ | CTAATATT |  | GTGTAGAA |  | ATTCCTAA |  |
| 345 | GTTAGAAG | GAATGAGG | ^195^ | TATGTACG |  | CACATACA |  | AGGCTAAG |  | CGCTATCC | ^480^ |
| 346 | GTGGGGAG | TGAGAAGC |  | GATGAGAA |  | CACACATA |  | TCACGTGC |  | ACCCGACC | ^44^ |
| 347 | TGGCCGGG | ACACACAC | ^201^ | GTGTTAGA | ^348^ | CCACACAC |  | GCGATGGC | ^31^ | TCATCTAC | ^468^ |
| 348 | GTGTTAGA | CTACCATC | ^319^ | GAAGAAGA |  | TACCCGGC |  | CGGCGGCT |  | GAATGAGG | ^195^ |
| 349 | CCGTGGAG | TACCCGAC | ^235^ | CACATATA |  | ACACCCAC |  | AAGAACAA |  | GTGTAGAA |  |
| 350 | CCGTATCG | TCAACTAT |  | CCTCATCG |  | TAGATAGA |  | ATATAAAA |  | GCCTTTAT |  |
| 351 | GACGAAGA | ATAGCCGC | ^165^ | TTTCCCTT |  | ACCACACC | ^190^ | TAAAATAT |  | GGGTTCGA | ^263^ |
| 352 | GCGCGCCA | ATAATGTA |  | TATATTAT |  | AGATGATG |  | AAGGGAAA |  | GGAATGAG | ^197^ |
| 353 | TTGTGTAG | CAACTATC | ^270^ | CACACATA |  | CGCTATCC | ^480^ | TTAATTTT |  | TAGATTCC |  |
| 354 | CTCCCAGC | CCTTTTTT |  | TAGATAGA |  | TTATTTTT |  | ATTATCAA |  | TCATCTCA |  |
| 355 | GCAACAGC | GCGCGCCA | ^352^ | CACATACA |  | AATCAACT |  | TGATGAGA |  | AATTTCAA |  |
| 356 | GCTGTTGC | TTCCTAAA |  | GCCTTTAT |  | CTATCGTC | ^251^ | GAAAATTT |  | CCGACTAC | ^40^ |
| 357 | GGGGCAGC | CCGGGTAG |  | TAAATAGA |  | ATCTAAAT |  | TAGAAGAT |  | TACGTTAC |  |
| 358 | GCTGCCGG | TTCCGCGG | ^161^ | TTTTCCTT |  | AAAAGGAA |  | TTCCTTTT |  | AAATCAAC |  |
| 359 | CCCGCGAG | AAAAAATA |  | CAAATGAT |  | ACTAACTA |  | GAATGAGG | ^195^ | AATTGCAG |  |
| 360 | CATCGTGC | ATGGATTC |  | TTTATATA |  | TTCTTTTC |  | TAGATTCC |  | ACAATAGA |  |
| 361 | GCCTCCGC | ATTGCAGA |  | ACTAACTA |  | GTCTATCA |  | ACTACTAC | ^406^ | TTCCGCGG | ^161^ |
| 362 | CGCGCTTC | TTTTCTTC |  | ATTTACAT |  | TTATCAAC |  | TTCTTTTC |  | ATATAGAT |  |
| 363 | TGATAATG | AATTGCAG |  | ATTATTAT |  | GTATGTAT |  | GAAGACGA | ^309^ | AGTGAAAA |  |
| 364 | GGCACACC | GCGTCACA |  | AATAATAT |  | CATCTACT | ^268^ | TCGTCAGA |  | CGTCTATC | ^228^ |
| 365 | GTGCACCA | TCATCTAC | ^468^ | CATCTACT | ^268^ | ATATTTAT |  | CCACACCC | ^148^ | AATGAATA |  |
| 366 | ACTCGCGC | ATTAGTGG |  | GTATGTAT |  | TACCCGCA |  | TAACAATA |  | AACAACAA | ^408^ |
| 367 | GAGCTCGA | GCGATGCC | ^175^ | AAGCAAAC |  | GGGCGGCT |  | GGAATGAG | ^197^ | GAAGACGA | ^309^ |
| 368 | AAGTCGCC | ATATTCTG |  | GGGTAACG |  | GTTAGAGG | ^143^ | AGGAAAAG |  | ACTACTAC | ^406^ |
| 369 | CTGCGCAG | ACACCCAC |  | TGGAATAA |  | GCCTTTAT |  | AAAATTTC |  | GTTACTAG |  |
| 370 | GCTGAAAC | TAATATAT |  | CCCATACA |  | CTCGGCGG | ^21^ | ACCACACC | ^190^ | TATAATAT |  |
| 371 | GCTTACGG | GCCGCACC | ^68^ | TCTTTTTT |  | TGTATACC |  | CTCGGCGG | ^21^ | CGGTTCGA |  |
| 372 | CGGTGCGG | AAAAATAA |  | AAATCAAC |  | TAGATTCC |  | GTATGGGT |  | GGGGGGAG | ^42^ |
| 373 | ACCTAATA | ACCCATAC | ^314^ | TCATCATC |  | GATGAGAA |  | TACGTTAC |  | AGCCGCCG |  |
| 374 | CACGTCGC | TTTTCACT |  | TGTATACC |  | TTCCATTT |  | GCGGCTAA | ^74^ | TACATATA |  |
| 375 | GCCTCCTC | TCTCATCG |  | AGGGAAAA |  | TTTTCACT |  | GGGTAACG |  | GGGCGGCT |  |
| 376 | ATATATAT | ATATGTAT |  | ATGATAAT |  | AAATAAAT |  | ATTGCAGA |  | AAATGATG |  |
| 377 | CGTCTGGA | AGAGGAAG |  | ATTCTGTA |  | AATAAAAA |  | TGTTTTGT |  | ATAATAAT |  |
| 378 | GCGGTCGC | TATATAAG |  | GCGATGGC | ^31^ | ACTACTAC | ^406^ | TTGGTCTT |  | TGATGAGA |  |
| 379 | GGTACCCT | AGTGAAAA |  | ACTACTAC | ^406^ | GTATGGGT |  | GTTACTAG |  | TCTTTTCT |  |
| 380 | CCCTCGCC | TTTATTTT |  | ATACGTAT |  | ATAAATAG |  | TCTTTTTC |  | AAAAGGAA |  |
| 381 | GGTAAGGG | CTAAATCC |  | GTATATAA |  | CACATATA |  | GGGGTTCG | ^45^ | TAGAAGAT |  |
| 382 | GCCTCGGC | CGAAGCGG | ^166^ | TTCACTTT |  | AAGCAAAC |  | GTATATTC |  | GGTGTTAA |  |
| 383 | CCGCCGAA | AGGCTGCG |  | AATGAAAA |  | CAAATGAT |  | CGGAACCG | ^343^ | AGCCCTAT |  |
| 384 | GAAACGCA | TTTCCTTT |  | TCACGTGC |  | ATACGTAT |  | TATATAAA |  | AATATTAA |  |
| 385 | TTACCCGC | CTCCGGGT |  | TATAGATT |  | ACATACAC |  | GGTTACCC |  | ATTGCAGA |  |
| 386 | GGTGTGCC | ACTACTAC | ^406^ | GTGTGTGT |  | ATATAAAT |  | GGCTAAGC | ^119^ | CTCGGATC | ^46^ |
| 387 | TATTACCC | TATATACA |  | ACCACACC | ^190^ | TAAATAGA |  | GCTAAGCG | ^125^ | CTGAAGTG | ^269^ |
| 388 | ACGCGCGC | TGACGCGT |  | AGAAAAAA |  | TACGTACA |  | TCAAAACA |  | TTGGTCTT |  |
| 389 | AAAAAGAA | GATGAGAA |  | AGAGGAAG |  | TTTTTTCT |  | CCGAGTGG | ^4^ | TCGTCTAT |  |
| 390 | CTGCAGCG | TCGTCAGA |  | GGTTACCC |  | GCGGCTAA | ^74^ | AACAAACA |  | GTGTTAAG |  |
| 391 | CCCGGGAG | ACTCCCAG |  | TTACGTTA |  | TATATAAG |  | CAACAGCA |  | AAGGGAAA |  |
| 392 | CCGGGGTT | TTCCTTTT |  | ACACCCAC |  | TTGTGTAG | ^353^ | GGTGTTAA |  | CTGGTTCT |  |
| 393 | CACGGAGC | TTTTTCAT |  | GTCTATCA |  | TTACGTTA |  | AATTTTCA |  | CAACTATC | ^270^ |
| 394 | GTCGCACC | ATTCTGTA |  | TTGTGTAG | ^353^ | AACAATAA |  | CAACTATC | ^270^ | CCTTCCCC |  |
| 395 | AAACGGAA | CGCGTCGC | ^7^ | ATCCGTAC |  | ATTTACAT |  | TGATAATA |  | ATTATCAA |  |
| 396 | CAGATTCC | ATTGATAA |  | TCTTTCTT |  | TTTCCCTT |  | AACAATAG |  | GTATGTAG |  |
| 397 | GATGACAT | CATCGCTA | ^232^ | CAAATCTA |  | CCACACCC | ^148^ | GACGAAGA | ^351^ | TGTTTTGT |  |
| 398 | CATATACG | TCATCTAA |  | CATATGTA |  | CAAATCTA |  | CGCTATCC | ^480^ | AAGAACAA |  |
| 399 | CACGAGCG | TTTCAAAA |  | TAGATTCC |  | GAAGACGA | ^309^ | AAATAGTC |  | TAAAATAT |  |
| 400 | GCGACCCG | ATGTAATA |  | ATTCCATT |  | ACGCAAAT |  | CTGGTTCT |  | TAACAATA |  |
| 401 | TGGCCGAG | TTCTTGGC |  | CCACACAC |  | TCATCATC |  | ATGATAAT |  | ATCGTCTA |  |
| 402 | GGCTCGCC | GGAATGAG | ^197^ | GAAGACGA | ^309^ | GCCGCCCA | ^61^ | CGTCTATC | ^228^ | CGAAGCGG | ^166^ |
| 403 | CCCGCTCG | TATTTATT |  | TCTTCCTC |  | ACCCGGAC |  | TCGTCTAT |  | CAACAGCA |  |
| 404 | GGGAGCGG | CTACATAC |  | CCATTGTT |  | ATTCTGTA |  | CTGAAGTG | ^269^ | TACCCGCA |  |
| 405 | CCACCGCC | CCTAAACG |  | TCTTTTCT |  | GTATACGT |  | CGGTACCC | ^194^ | GATCTCGC | ^73^ |
| 406 | ACTACTAC | GCTGTCAT |  | ACGCAAAT |  | GAAGAAGA |  | GTATGTAG |  | TTAATTTT |  |
| 407 | CAGGCGCG | AAAAAAGG |  | AATACATA |  | TGGAATAA |  | CACCTCCG | ^34^ | AGGAAAAG |  |
| 408 | AACAACAA | TATACCTA |  | ACATACAC |  | AAATCAAC |  | TACATACA |  | GACGAAGA | ^351^ |
| 409 | CGTCGAGG | GCCACCGG | ^239^ | GACGACGA | ^203^ | CATATGTA |  | AAAATTCA |  | GTATATTC |  |
| 410 | GGCCCGGC | AGCGATGA |  | CTATCGTC | ^251^ | CTATCATC |  | CATCTACT | ^268^ | GAAAATTT |  |
| 411 | ATATCCTC | GCCCGGCG | ^313^ | ACCCTACC | ^168^ | CAACTATC | ^270^ | AAATTTTC |  | ATATAAAA |  |
| 412 | GCGACAGC | GTCATCGA |  | GTTAGAGG | ^143^ | GCTTATCT |  | GTGTTAAG |  | CATCTACT | ^268^ |
| 413 | GCGGCTGA | CAGGCCGG | ^115^ | CTATCATC |  | TATAGATT |  | ATATACAT |  | AGATTCCC |  |
| 414 | TTCTTTTT | GAAGAAGA |  | CGTCTATC | ^228^ | AGAGGAAG |  | GGGTTCGA | ^263^ | TTCCTTTT |  |
| 415 | GGATCAAT | TATTATAG |  | AAGGAAAA |  | ATGATAAT |  | AGATGATG |  | AAAATTTC |  |
| 416 | GATCGGGC | TGGACCCT |  | AATTGAAA |  | TCGGTAGC | ^240^ | TTATAGAA |  | TCAAAACA |  |
| 417 | CCCACCGC | CACTCCGG | ^79^ | GCTATTGT |  | TACGTTAC |  | TTTCAGTT |  | GTATACGT |  |
| 418 | TCGAGCCC | TGGAATAA |  | CAACTATC | ^270^ | ATACACAC |  | TTTTTCAC |  | AAATAGTC |  |
| 419 | CATCGGAA | TCCCTTTT |  | AATGAATA |  | CCATTGTT |  | AAATCCAC |  | TTCTTTTC |  |
| 420 | CCGCGCCC | TTTTCAAG |  | TACCCGCA |  | GCTATTGT |  | CATATATA |  | CAATACCA |  |
| 421 | CACTGGGG | AATTGAAA |  | TACGTACA |  | GTTACTAG |  | ATCTACTA |  | GCGACGCG | ^15^ |
| 422 | GCATGCGA | CTGGCCTA |  | TGAAATTT |  | TCGTCTAT |  | AGGGAAAA |  | AAATCCAC |  |
| 423 | AGGGGTCC | TGCCGTCC | ^185^ | ATTTACGT |  | TATATTAT |  | CAATACCA |  | GCTTATCT |  |
| 424 | GTCCCCGG | CAGATTCC | ^396^ | GTATACGT |  | ATTATTAT |  | GTTTTATA |  | CGGCACCC | ^90^ |
| 425 | TTGGAATA | CCATTTTG |  | ATAGATTC |  | AGGGAAAA |  | TTCCGCGG | ^161^ | TATGGGTG |  |
| 426 | CCCCGGGA | GATGACAT | ^397^ | TAACAATA |  | TAACTAGT |  | AGTATGTA |  | TACCCGGC |  |
| 427 | CATCCGTA | ACCCCTCG |  | TAACTAGT |  | CCTAAACG |  | AGCCCTAT |  | CTAGACTT |  |
| 428 | GGCCCCGC | TATTATTA |  | GCTTATCT |  | TCTTCCTC |  | TTCATTTT |  | AACAAACA |  |
| 429 | GGCACCTC | TCTCGAGC |  | ATACACAC |  | ATAGATTC |  | ATCGTCTA |  | AACAATAG |  |
| 430 | CCGTTACC | ATATATTA |  | TACCCGAC | ^235^ | ATTTACGT |  | TTATTTTA |  | ATCTACTA |  |
| 431 | GAGACCGG | TGATAATA |  | AGGAAAAG |  | TTCACTTT |  | GTATACGT |  | TACATACA |  |
| 432 | CCGGGTCA | TATTCTGT |  | GATGACAT | ^397^ | TTTTCCTT |  | GCTTATCT |  | GCAACAGC | ^355^ |
| 433 | GCACCTGC | GTCATCTA |  | GTTAGAAG | ^345^ | ATTCCATT |  | ATTCCTAT |  | TAGTCATC |  |
| 434 | CCTTTATC | AAGGGTTT |  | TTTCATTT |  | CACATACG |  | TAGTCATC |  | AGATGATG |  |
| 435 | AGACCGGG | GTTACTAG |  | TATCAACA |  | AGGGTCCA |  | CCTTCCCC |  | GGTACCCT | ^379^ |
| 436 | GCTGCCTC | TATATAAT |  | AGCCGCCG |  | AATAATAT |  | TATCAACA |  | TAGACTTA |  |
| 437 | CGTGGCGC | TATAATAT |  | CGCTATCC | ^480^ | TTTATATA |  | ACGCAAAT |  | ACGCAAAT |  |
| 438 | GCGGGCCG | CTATATCC |  | TACCCGGC |  | GATGACAT | ^397^ | TATAAAAG |  | AGTATGTA |  |
| 439 | GGTGGCGA | TTTTCCTT |  | GAAGAAAA |  | GTATATAA |  | TACCCGCA |  | GGTTAAGG | ^271^ |
| 440 | CCGCCTCC | CGGCCTCC | ^96^ | CAAAACAA |  | CTGTCATC | ^326^ | CCGACTAC | ^40^ | CAAGTGCA |  |
| 441 | GCACACGC | TTTTCAAT |  | GTATGGGT |  | CGCTACCA | ^179^ | AGATTCCC |  | GCAGTAGC | ^261^ |
| 442 | CCCGGTCG | AAAAGGAA |  | TACTACTA |  | GTTAGAAG | ^345^ | GAATAAAA |  | ATTCCTAT |  |
| 443 | GGTGACCC | TATATTCT |  | TAGTATGT |  | TAGTATGT |  | GGGCGGCT |  | ATGATAAT |  |
| 444 | AAACGCAA | AATTTTCA |  | GTTACTAG |  | AATACATA |  | TCTCATCG |  | TCTTTTTC |  |
| 445 | CTCCCCAC | AGAAGAAA |  | AATTGCAG |  | CGCGCGCG | ^48^ | TCATCGCA |  | GGAATCCC | ^298^ |
| 446 | GGGCGAGC | CTCGGATC | ^46^ | TACGTTAC |  | AGGATTCC |  | GAGAAGCT |  | TGATAATA |  |
| 447 | GCGGGGTA | AAGGAAAA |  | TCGTCTAT |  | CGCATACG | ^272^ | TAGTGGAA |  | CCTAAACG |  |
| 448 | GTGGCAAA | TCGAGCCC | ^418^ | CCCAGCGC | ^3^ | GAATGAGG | ^195^ | CGGTTCGA |  | ACGTTACT |  |
| 449 | ATTACCCG | TATTTATA |  | CTGTCATC | ^326^ | TCGATGAG |  | TAATTAAT |  | AATTTTCA |  |
| 450 | CGAGCCCC | TTTTATTT |  | GCGGCTAA | ^74^ | CGGAATGA | ^292^ | AGCCGCCG |  | TCTCATCG |  |
| 451 | GAAGCGGC | AAAAAGGA |  | TATGTAGA |  | TTGCGATG |  | TATACGTA |  | GAGAAGCT |  |
| 452 | TCCGGGTA | AAAATAAA |  | TTATATAT |  | AATGAAAA |  | GCAACAGC | ^355^ | TCATCGCA |  |
| 453 | GGAGCCCT | TTATCAAC |  | TTGTTCTT |  | TATGTAGA |  | TGCAGATT |  | ATTAGTGG |  |
| 454 | TTCTCCTC | AAGATGAC |  | GATGATAA |  | TACTACTA |  | GATAATGA |  | GCTGTCAT |  |
| 455 | GCCCGTAA | GATCTCGC | ^73^ | TTACTATT |  | CATACGTA |  | AAGTTATG |  | TATATAAA |  |
| 456 | GGGGCCGG | TATCATAT |  | ATTTTTTT |  | GCGCGCGC | ^12^ | CAAGTGCA |  | CGCGTCGC | ^7^ |
| 457 | GCGTGCGC | TTTTGAGG |  | ACTATCAT |  | TGCAGATT |  | ATTAGTGG |  | TTTCAGTT |  |
| 458 | GGAGGAGG | TTAGAAGA |  | TGCAGATT |  | AATTGCAG |  | TATATGTA |  | TTATAGAA |  |
| 459 | GCCGCGTC | CTATTGTT |  | ACTAATAG |  | CGGGTAAG |  | CTTTATCA |  | AAGTTATG |  |
| 460 | GCTGGTCG | GTGTAGAA |  | GGGCGGCT |  | TTACCCTA |  | TCATCTAA |  | TGCAGATT |  |
| 461 | CACCGTAT | TTCTTTTC |  | CCACACCC | ^148^ | TCTTTTTT |  | ACGTTACT |  | GCCGCCCA | ^61^ |
| 462 | CTGGTCGC | TCTTCCTC |  | GAATGAGG | ^195^ | TCCGCGGA |  | TATTTTAT |  | AAAATTCA |  |
| 463 | GAGCCCGC | GCTGCTGC | ^332^ | ATAGTCAT |  | TCTTTCTT |  | TTTTACTT |  | GTTTTATA |  |
| 464 | CTGCGCGG | AGCGTAGG | ^229^ | TTTCAGTT |  | AATGAATA |  | TCTTTCTT |  | GATATCAG |  |
| 465 | TGACCCGC | CGCTATCC | ^480^ | TTGCGATG |  | TGTCATCG |  | GCTGTCAT |  | GCGGTACC | ^107^ |
| 466 | AGGACGCC | AATCAACT |  | AGGATTCC |  | ACTAATAG |  | TTCTGTAT |  | TTACCCTA |  |
| 467 | GGAGAGGG | TACGTTAC |  | TGTACATA |  | GAGGATTC | ^178^ | TATGGGTG |  | TAGTGGAA |  |
| 468 | TCATCTAC | CTTTATCA |  | TCTCATCT |  | CGCGTCGC | ^7^ | AAACCAAA |  | ATCGTAAT |  |
| 469 | GAGACTAG | CGCGCTTC | ^362^ | ACACATAT |  | TATCAACA |  | ATTTTATT |  | TATACGTA |  |
| 470 | CGTGGCCC | GCAGCGAT |  | TTTGTTTA |  | TAACAATA |  | ATCGTAAT |  | AGGGAAAA |  |
| 471 | CTACTCGG | GCCTTCTT |  | GTTAAGAT |  | GGAATGAG | ^197^ | TTACCCTA |  | GCGCAGGC | ^234^ |
| 472 | AAGCCGGG | CCTAAATC |  | TTACCCTA |  | TCTTTTCT |  | TAGACTTA |  | CGCATACG | ^272^ |
| 473 | GCGCTACC | GGGTTCGA | ^263^ | TCGATGAG |  | AGCGTAGG | ^229^ | TAAGTTAT |  | TATCAACA |  |
| 474 | GGCGTCGG | CCCATACA |  | AAAGAAAG |  | ACTATCAT |  | GTCATCTA |  | ATATACAT |  |
| 475 | GCGCAAGC | TACCCTCG |  | AACGGAAT |  | AATTGAAA |  | TTGAAATT |  | AAATTTTC |  |
| 476 | GGGTTACC | AAGAAGAA |  | GGGGCGGG | ^55^ | AACGGAAT |  | GATATCAG |  | TTGGCGTT |  |
| 477 | CCTTGAGG | GCGGCTAT | ^495^ | AACAAGAA |  | GTTAAGAT |  | ACCCGACC | ^44^ | GTACCCTC | ^296^ |
| 478 | CAGCCGCT | GCGGTACC | ^107^ | AAACGGAA | ^395^ | TGAAATTT |  | TTCCTAAA |  | CGAAGACG |  |
| 479 | GCTCATCG | GAGGAATA |  | TCCTCATC | ^484^ | GCGACGCG | ^15^ | TAGTTTTG |  | GTCATCTA |  |
| 480 | CGCTATCC | ACCCGGAC |  | CTAAATAA |  | AGGAAAAG |  | GCAGTAGC | ^261^ | TCCGTACA |  |
| 481 | TACCCGGA | CAACTTCT |  | CACATACG |  | GCTGCTGC | ^332^ | CTAGACTT |  | AGCTAGAG |  |
| 482 | CCTGCGCG | GGAATAAA |  | CGGAATGA | ^292^ | AGAAAAAA |  | CGAAGCGG | ^166^ | GATAATGA |  |
| 483 | CCATCAGC | TACATATA |  | AAATGAAA |  | ATAGTCAT |  | TATATATG |  | TGTGTGTG | ^252^ |
| 484 | TCCTCATC | TAGTGGAA |  | CATACGTA |  | GATGATAA |  | AAAGGGAA |  | ACGAAGAC |  |
| 485 | GCGTAGCG | CCCTTTTT |  | GTATTTAC |  | TCCTCATC | ^484^ | TTGGCGTT |  | TTTTTCAC |  |
| 486 | CGGCTCCC | AGCTCTTT |  | TGTCATCG |  | AAGGAAAA |  | TTAGAAGA |  | TCATCTAA |  |
| 487 | ATCCCCGC | GAAGCTGT |  | GTATATTC |  | AGATTCCA |  | AGTCATCT |  | CAGCCCTA |  |
| 488 | GGTTAGGG | ACCCGGAT |  | AGATTCCA |  | TGTACATA |  | TACGTATA |  | CATATATA |  |
| 489 | GGGGACAG | AATGAAAA |  | CCTAAACG |  | CAAAACAA |  | TTTACTTT |  | GTCATCGA |  |
| 490 | CACACGCA | CAGCCGCT | ^478^ | CGCTACCA | ^179^ | TACGTACG |  | GATCTCGC | ^73^ | CACAATAC |  |
| 491 | GATTCCAT | TTCTTCTT |  | CCATTTTG |  | TCTCATCT |  | GGGGGGAG | ^42^ | TTATTTTA |  |
| 492 | CGCCCGCG | GACGCGCC | ^41^ | CTAGTATT |  | CTACCATC | ^319^ | ATTGTGTA |  | TAAGTTAT |  |
| 493 | GCAGGAGC | GGTTACCC |  | AGTATATT |  | GGCTAAGC | ^119^ | GGTTAAGG | ^271^ | TTCATTTT |  |
| 494 | ACGTGAGC | GCCAAGAA |  | CTTTCTTT |  | TTACTATT |  | TGTGTGTG | ^252^ | TAATTAAT |  |
| 495 | GCGGCTAT | TCTCCAAC |  | GAGGATTC | ^178^ | GTGTATGT |  | TATCTATA |  | TATAAAAG |  |
| 496 | GGACCCCA | AGCAGCAG |  | GCCGCCCA | ^61^ | GCTAAGCG | ^125^ | CTCGGATC | ^46^ | AGTCATCT |  |
| 497 | CCCCGCCC | CTTCGAGA |  | TTGCAGAT |  | ACACATAT |  | ACTATCAT |  | GTTATGAG |  |
| 498 | GCGGCGAC | ACCCGGCG |  | CTCGGCGG | ^21^ | TTTCATTT |  | AATATTTA |  | ATCTGCTC |  |
| 499 | CCCCGGTT | TTCTCGAG |  | TCTGTATA |  | GTATTTAC |  | AGAGGAAG |  | CTTTATCA |  |
| 500 | TGGATTCC | TGCGGCAC |  | GGAATGAG | ^197^ | ATCTGCTC |  | TTATCAAC |  | TAGTTTTG |  |
| Avg  GC% | 68.4 | 40.2 |  | 33.7 |  | 35.4 |  | 35.6 |  | 37.4 |  |
| Percentage of common k-mers |  | 42.6 |  | 33.4 |  | 35.4 |  | 36.0 |  | 38.0 |  |
