# Supplementary material for: Comparative Analysis of DNA Word Abundances in Four Yeast Genomes Using a Novel Statistical Background Model
Source: PLoS One. 2013 Mar 5;8(3):e58038. doi: 10.1371/journal.pone.0058038 (PMC3589456; doi:10.1371/journal.pone.0058038)
Supplement: Table S5 — Overrepresented k-mers (b) enriched in the 500 bp upstream regions of ORFs in the S. cerevisiae genome. The k-mer lengths (a) their presence in the overrepresented lists of the S.bayanus, S.paradoxus and S.mikatae genomes (c) along with their matches to known motifs from Yeastract (d). (DOC) [file pone.0058038.s026.doc]

**Supplementary table S26**

| **No** | **k-mer length (nt) (a)** | **Sequence (b)** | **Conservation**  **(c)** | **Yeastract motif (d)** |
| --- | --- | --- | --- | --- |
| 1 | 11 | AATGAAAAATT | Found in all four genomes | ESR2 motif |
| 2 | 11 | AGCGATGAGAT | Found in all four genomes | ESR1 GATGAG motif |
| 3 | 11 | AGTGAAAAATT | Found in all four genomes | ESR2 motif |
| 4 | 12 | ATGAAAAATTTT | Found in all four genomes | ESR2 motif |
| 5 | 8 | GCGATGAG | Found in all four genomes | ESR1 GATGAG motif |
| 6 | 11 | AAAAATTTTCA | Found in three genomes | ESR2 motif |
| 7 | 10 | TCGGCGGCTA | Found in all four genomes | UME6 binding site |
| 8 | 12 | GCTGAAAATTTT | Found in three genomes | ESR2 motif |
| 9 | 10 | TTGCGATGAG | Found in all four genomes | ESR1 GATGAG motif |
